# A fat body transcriptome analysis of the immune responses of *Rhodnius prolixus* to artificial infections with bacteria

**DOI:** 10.1186/s13071-022-05358-9

**Published:** 2022-07-29

**Authors:** Nicolas Salcedo-Porras, Pedro Lagerblad Oliveira, Alessandra Aparecida Guarneri, Carl Lowenberger

**Affiliations:** 1 Centre for Cell Biology, Development and Disease, Department of Biological Sciences, Simon Fraser University, 8888 University Drive, Burnaby, BC V5A 1S6 Canada; 2 Instituto de Bioquímica Médica Leopoldo de Meis, Universidade Federal do Rio de Janeiro, Avenida Carlos Chagas Filho, 373, Bloco D. Prédio do CCS, Ilha do Fundão, Rio de Janeiro, 21941-902 Brazil; 3 Vector Behavior and Pathogen Interaction Group, Centro de Pesquisas René Rachou, Fiocruz, Avenida Augusto de Lima, 1715, Belo Horizonte, MG CEP 30190-009 Brazil

**Keywords:** Transcriptome, Innate immunity, Toll pathway, Immune deficiency pathway, Triatomines, Serine proteases, Serine protease inhibitors

## Abstract

**Background:**

*Rhodnius prolixus* is an important vector of *Trypanosoma cruzi*, the causal agent of Chagas disease in humans. Despite the medical importance of this and other triatomine vectors, the study of their immune responses has been limited to a few molecular pathways and processes. Insect immunity studies were first described for holometabolous insects such as *Drosophila melanogaster*, and it was assumed that their immune responses were conserved in all insects. However, study of the immune responses of triatomines and other hemimetabolous insects has revealed discrepancies between these and the *Drosophila* model.

**Methods:**

To expand our understanding of innate immune responses of triatomines to pathogens, we injected fifth instar nymphs of *R. prolixus* with the Gram-negative (Gr−) bacterium *Enterobacter cloacae*, the Gram-positive (Gr+) bacterium *Staphylococcus aureus*, or phosphate-buffered saline (PBS), and evaluated transcript expression in the fat body 8 and 24 h post-injection (hpi). We analyzed the differential expression of transcripts at each time point, and across time, for each treatment.

**Results:**

At 8 hpi, the Gr− bacteria-injected group had a large number of differentially expressed (DE) transcripts, and most of the changes in transcript expression were maintained at 24 hpi. In the Gr+ bacteria treatment, few DE transcripts were detected at 8 hpi, but a large number of transcripts were DE at 24 hpi. Unexpectedly, the PBS control also had a large number of DE transcripts at 24 hpi. Very few DE transcripts were common to the different treatments and time points, indicating a high specificity of the immune responses of *R. prolixus* to different pathogens. Antimicrobial peptides known to be induced by the immune deficiency pathway were induced upon Gr− bacterial infection. Many transcripts of genes from the Toll pathway that are thought to participate in responses to Gr+ bacteria and fungi were induced by both bacteria and PBS treatment. Pathogen recognition receptors and serine protease cascade transcripts were also overexpressed after Gr− bacteria and PBS injections. Gr- injection also upregulated transcripts involved in the metabolism of tyrosine, a major substrate involved in the melanotic encapsulation response to pathogens.

**Conclusions:**

These results reveal time-dependent pathogen-specific regulation of immune responses in triatomines, and hint at strong interactions between the immune deficiency and Toll pathways.

**Graphical abstract:**

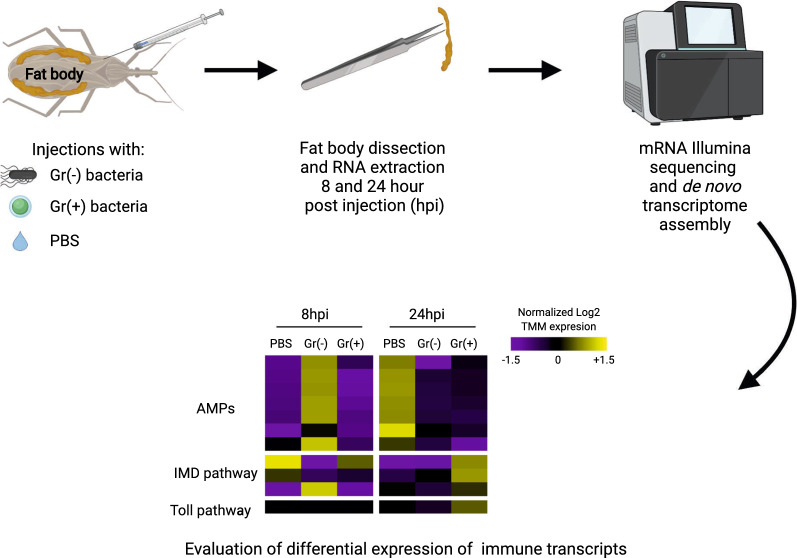

**Supplementary Information:**

The online version contains supplementary material, which is available at 10.1186/s13071-022-05358-9.

## Background

*Rhodnius prolixus* is a hemimetabolous obligate hematophagous insect and a major vector of *Trypanosoma cruzi*, the parasite that causes Chagas disease in humans, which is responsible for ~ 10,000 deaths each year [1]. This insect has served as a model for insect physiological studies for over 80 years [2]. The sequencing and assembly of the *R. prolixus* genome in 2009 allowed the first description of its genome-encoded immune repertoire and opened up new possibilities for the development of strategies to control the spread of Chagas disease [3].

Insects have evolved complex innate immune systems to recognize and eliminate pathogens. Most insects use common receptors and molecular pathways for conserved or similar functions. However, each insect has undergone specific expansions and contractions of immune gene families [4–10]. These adaptations are likely a consequence of selection pressures imposed by pathogens and an insect’s environment [11, 12]. Immune responses are initiated by the recognition of pathogens as non-self. This process is achieved by the binding of pattern recognition receptors (PRRs) of the insect to different pathogen-associated molecular patterns (PAMPs) that characterize distinct groups of microorganisms [13–15]. Damage-associated molecular patterns (DAMPs) are released from injured or stressed host tissues and can activate immune responses, but their receptors have not been well characterized in insects [16]. These PRR-PAMP interactions activate individual or multiple immune signaling pathways that ultimately control immune responses including phagocytosis, melanotic encapsulation, and the production of effector antimicrobial peptides (AMPs) [17–19]. In the pioneering studies on *Drosophila melanogaster* it was found that the immune deficiency (IMD) pathway regulated immune responses to Gram-negative (Gr−) bacteria, the Toll pathway regulated responses to fungi and Gram-positive (Gr+) bacteria, and the Janus kinase-signal transducer and activator of transcription and RNA interference pathways regulated responses to bacteria, viruses, and parasitoids [17–22]. We now recognize that these pathways are not controlled so rigidly; there is cross-talk among and between pathways, some pathways share intermediate molecules, and the expression of some AMPs may be co-regulated by multiple pathways [10, 23–25].

The IMD pathway uses membrane-bound or intracellular peptidoglycan (PGN) recognition proteins (PGRPs) as PRRs to detect meso-diaminopimelic acid-type PGNs that are found on the cell wall of Gr- bacteria and *Bacillus* sp. [18]. Once bound to PGNs, PGRPs form homodimers and recruit the adaptor protein IMD and the fatty acid synthase-associated death domain protein. Fatty acid synthase-associated death domain protein and IMD are cleaved by the caspase Dredd and ubiquitinated by other proteins. These events culminate in the translocation of the NF-κΒ transcription factor Relish into the nucleus, where it induces the transcription of AMPs and initiates other immune responses [19]. In *R. prolixus*, the IMD pathway was originally described as being incomplete or non-functional [3], but most of its “missing” genes were identified in subsequent studies [26–28]. These studies also showed that the IMD pathway in *R. prolixus* regulates responses to Gr- bacteria and potentially Gr+ bacteria [26, 27].

The Toll pathway in insects controls immune responses, but is also involved in wound healing, and development [17]. The activation of these processes relies on PRRs and cascades of serine proteases (SPs) that rapidly amplify the recognition of PAMPs. SP inhibitors (SPI) regulate the activation of these responses by inactivating or modulating SPs [29–31]. The number of proteins involved in these events varies between different species, and these proteins have been best characterized in *D. melanogaster* and the silk moth *Manduca sexta* [32–34]. In these holometabolous insects, the immune responses mediated by the Toll pathway use extracellular PGRPs and Gr− binding proteins (GNBPs) to detectl-lysine-type PGN from Gr+ bacteria and β-1,3-glucans from fungi. These recognition events trigger an extracellular cascade comprised of three to five SPs that eventually cleave and activate the Toll-receptor ligand Spätzle which recruits the adaptor protein Myd88, the protein tubulin epsilon, and the kinase Pelle [5, 32, 34]. This complex of proteins phosphorylates the negative regulator Cactus, triggering its degradation. This permits the translocation of the NF-κΒ transcription factors Dorsal/Dif into the nucleus, which induces the transcription of AMPs and other immune responses [5, 32, 34].

*Rhodnius prolixus* has orthologs encoding all of the intracellular proteins of the Toll pathway described for holometabolous insects [3]. However, genes encoding extracellular PRRs, SPs, and SPI have not been studied in triatomines. In *M. sexta*, these proteins also regulate the prophenoloxidase (PPO) and melanization responses [34]. In *D. melanogaster*, however, a different subset of SPs is activated by wounding and damage signals, which regulate the PPO and melanization processes [32].

Recent transcriptomic studies on *R. prolixus* and other triatomines have allowed for the comparative study of differential responses in different insect groups through the simultaneous analysis of hundreds of molecules [28, 35–47]. However, to date, no transcriptomic study has focused solely on triatomine immune responses in the fat body (FB), which is considered to be the principal immune responsive tissue in insects. In the present study, we generated, assembled, and analyzed FB transcriptomes of fifth instar nymphs of *R. prolixus* at 8 and 24 h after they had been injected with phosphate-buffered saline (PBS), Gr−, or Gr+ bacteria. Although bacterial infections can affect a plethora of biological and metabolic processes, we focused our analysis on the differential expression of gene transcripts known to participate in immune responses. We used these transcriptomes previously to identify and characterize PGRPs involved in the IMD pathway [27]. In this study, we expanded our analysis to all immune pathways and evaluated their patterns of differential expression.

## Methods

### Insect rearing

We used a colony of *R. prolixus* that is maintained in the insectary of the Institute of Medical Biochemistry at the Federal University of Rio de Janeiro (UFRJ). This colony was originally established at UFRJ between 1979 and 1981, from a colony obtained from the Universidade Federal Fluminense, Rio de Janeiro. The size of this colony has been maintained at approximately 100,000 insects since 1985, with an average of 2000 adult females. Insects used for this study were fed on live rabbits at 3-week intervals and maintained at 28 °C and 80–90% relative humidity and under a natural illumination cycle of approximately 13 h light and 11 h dark during the period of sampling. Recently molted (1–2 day old) fifth instar nymphs were used in our experiments, and were maintained under the standard rearing conditions described. Animal care and experimental protocols were established following the guidelines of the institution’s care and use committee (UFRJ Committee for Evaluation of Animal Use for Research), which are based on the National Institutes of Health Guide for the Care and Use of Laboratory Animals (ISBN0-309-05377-3). The protocols were approved by the Committee for Evaluation of Animal Use for Research of the UFRJ under registry number 115/13.

### Experimental design

To assess the immune response in *R. prolixus*, we created a FB transcriptome from fifth instar *R. prolixus* injected with the Gr− bacteria *Enterobacter cloacae*, the Gr+ bacteria *Staphylococcus aureus*, or PBS, as described previously [27]. These bacteria were selected as they have different PGNs in their cell walls that activate different immune responses in insects [5, 18, 32, 34]. Bacteria were grown in Luria–Bertani broth overnight at 200 r.p.m. and 37 °C, as described previously [26]. The liquid cultures were centrifuged, and pellets were washed three times in PBS (137 mM NaCl, 2.7 mM KCl, 10 mM sodium phosphate at pH 7.2). The PBS used in the control injections, and to resuspend the bacteria, was autoclaved and filtered through a 0.2-µm filter. The concentration of bacteria in PBS was calculated using light absorbance at 600 nm on a Shimadzu UV-2550 spectrophotometer. The cuticle around the injection site of each insect was cleaned with 75% ethanol by using a sterile cotton swab. Insects were then injected directly into the hemocoel with 1 µL of PBS containing approximately 1 million bacteria. This was done using a 10-µL Hamilton syringe inserted into the right metapleura. Control insects were injected with 1 µL PBS. All insects were injected with PBS or bacteria in a single session, 4–5 h after sunrise. This approach allowed us to distinguish immune responses caused by the PBS injection from responses elicited by each type of bacteria. We used this methodology previously with the UFRJ *R. prolixus* colony to characterize the transcription of IMD pathway genes in insects injected with bacteria [3, 26, 27].

FB tissues were dissected 8 and 24 hpi to enable the identification of immune genes expressed early or later after injection, as characterized and reported in other studies [3, 10, 26, 27, 46, 47]. Total RNA was extracted from FB tissues using Trizol reagent (Invitrogen, Carlsbad, CA) following the manufacturer’s recommendations. Each of the six treatment groups had three biological replicates, each of which was derived from the FB of five insects. This design allowed us to study differentially expressed (DE) transcripts (i) over time within the same treatment group, and (ii) between treatment groups at the same time points.

### Sequencing and transcriptome assembly

Library construction and Illumina sequencing were done at the Biomedical Research Centre sequencing core at the University of British Columbia. RNA sequencing libraries were constructed using the NEBNext Ultra II RNA Library Prep Kit (New England Biolabs, Canada). Sequencing was performed on the Illumina NextSeq 500 with paired-end [80 base pairs (bp) × 80 bp] reads. Sequence data were demultiplexed using Illumina's bcl2fastq2. The process yielded 560,758,510 total raw reads (∼ 31 million reads per sample). Sample quality processing was done using established protocols [48]. First, sequence quality correction and adaptor trimming were performed with rCorrector [49] and TrimGalore using the parameters –length 70 -q 20 –stringency 1 -e 0.1 (https://www.bioinformatics.babraham.ac.uk/projects/trim_galore/). Ribosomal RNA contamination was removed by mapping the reads to the ribosomal RNA SSUParc and LSUParc from SILVA [50] with bowtie2, and using the very-sensitive-local mapping option [51]. Only paired reads were used in the final assembly. Reads were analyzed with FastQC [52] and MultiQC software [53] throughout the quality processing checking sequence quality, read length distribution, adapter content, and k-mer content. All remaining paired reads were used as inputs for the de novo transcriptome assembly using Trinity with default parameters [54]. The completeness of the assembled transcriptome was assessed by the BUSCO pipeline using the OrthoDB v. 10 dataset for insect genes [55]. The transcript sequences used in this study have been deposited in the Sequence Read Archive (SRA) repository of the National Center for Biotechnology Information (SRA accession number PRJNA755997).

### Differential expression analyses

Detection and quantification of DE transcripts were done using Trinity [54]. Differential expression analyses were done at the transcript level (called gene isoforms in Trinity). This analysis of transcripts maximizes the number of analyzed sequences and discriminates gene isoforms (hereafter referred to as “transcripts”) with different expression patterns. Firstly, transcript abundances were estimated using Trinity align_and_estimate_abundance.pl script, RSEM [56], and bowtie2 [51]. Secondly, transcript count matrices were generated with Trinity abundance_estimates_to_matrix.pl script. Thirdly, DE transcripts were found using Trinity run_DE_analysis.pl, and edgeR [57]. Only transcripts with at least 10 counts per million and present in at least two replicates were included in subsequent analyses. Pairwise comparisons were made among selected treatments and transcript expression values are reported as log twofold changes (log 2FCs). Transcripts that had a false discovery rate* P*-value < 0.05 were considered DE and included in subsequent analyses. Transcript names are based on Trinity standard nomenclature (e.g., DN2173_c1_g1_i9), “DN” represents de novo assembly, “c” indicates contigs, “g” indicates genes within a contig, and “i” indicates transcripts of a gene.

The transcriptome assembly was annotated using the Trinotate v. 3.1.1 pipeline (https://github.com/Trinotate/Trinotate.github.io). First, the most likely longest open reading frame peptide for each transcript was obtained using Transdecoder v. 5.0.2 (https://github.com/TransDecoder/TransDecoder). Secondly, transcript nucleotide and Transdecoder-predicted sequences were compared against the UniProt/Swiss-Prot release 2021_02 database using blastx and blastp algorithms. Search results were filtered using an e-value threshold of 0.0001, and only the best hit in each blast search for each transcript was used for further processing. Third, Transdecoder-predicted peptides were screened for (i) protein domains using HMMER v. 3.1 [58] and the Pfam release 31.0 [59], (ii) transmembrane domains using TmHMM v. 2.0c [60], and (iii) signal peptides using SignalP v. 4.1 [61]. Gene Ontology (GO) enrichment analysis of DE transcripts was performed using Trinity analyze_diff_expr.pl script, GOseq R package, and Fisher’s exact test from the OmicsBox v. 1.4.12 software.

### In silico evaluation of immune responses in insects injected with PBS

To evaluate if the PBS injection stimulated immune responses in the absence of bacteria (see the “Results” and the “Discussion” sections), we compared our transcriptome to the FB transcriptome from Ribeiro et al. [38]. This comparison was done because our samples (including the PBS controls) had high levels of transcripts from immune effector genes (see the “Results” section). The Ribeiro et al. [38] study used FB tissues from insects from the same UFRJ *R. prolixus* colony, but that were not exposed to bacteria. This is the closest available data set to a naïve control group available with which to perform this comparison. The FB transcriptome from Ribeiro et al. [38] was built using a pool of FB tissues dissected from five adult females at each time point 12-, 24-, 48-, and 128 h after feeding, and was sequenced on a 454 GS FLX Titanium sequencer (Roche Life Sciences, Branford, CT) [38]. Due to differences between the two studies, we did an additional quantification of gene expression using the *R. prolixus* genome V4.3 as a reference. Reads from our transcriptome and that of Ribeiro et al. [38] were mapped using STAR [62] and quantified using Featurecounts [63]. The 50 most highly expressed genes in our six treatments and those from Ribeiro et al. [38] were analyzed for GO enrichment of biological terms as 96% of the reads from Ribeiro et al. [38] mapped to this number of genes.

### Phylogenetic analyses of SPs and SPIs

Multiple protein sequence alignments were made for SPs and SPIs identified in the annotation of our transcriptome. To identify SPs and SPIs in *R. prolixus*, we searched the FB transcriptomes for sequences similar to SPs and SPIs from other insects, or sequences annotated with the PFAM ID PF05577 for SP and PF00079 for SPI, or GO terms for SPs and SPIs. Full-length representative sequences from insects with well-characterized SPs and SPIs were selected and included in the alignments (Additional file 1: Table S8). Protein alignments were made using MUSCLE with default parameters in the program MEGA X [64]. Maximum likelihood analyses were done using IQ-TREE v. 2.0 [65]. The best-fit model for each alignment was selected based on the Bayesian information criterion. Branch support was assessed by 1000 ultrafast bootstrap replicates.

## Results

To study the transcriptomic changes in *R. prolixus* following bacterial injection, we injected fifth instar nymphs with Gr− or Gr+ bacteria. Fat body extracts were prepared from tissue sampled at 8 and 24 hpi. The assembled transcriptome contained 52,076 genes and 127,769 unique transcripts (i.e., gene isoforms). The average contig length was 951 bp and the N50 was 2,767 bp (where N50 is the length of the shortest contig for which longer contigs and contigs of the same length comprise at least 50 % of the transcriptome assembly). The estimated BUSCO coverage of the transcriptome for insect genes was 95.7% for complete genes (41.4% as single-copy genes and 54.1% as duplicate genes), 1.2% for fragmented genes, and 3.1% for missing genes, confirming a high-quality assembly. A total of 18,089 transcripts corresponding to 10,234 genes had CPMs > 10 in at least two replicates, and were used for DE analyses. Of these, 622 transcripts for 495 genes were DE in at least in one comparison (Additional file 1: Table S1). We analyzed DE results for transcripts as most DE patterns were similar at the gene and transcript levels. Our analyses at the transcript level permitted us to discriminate gene isoforms with different expression patterns and account for genes with similar sequences which are reported as a single gene with multiple isoforms in our assembly pipeline. These differences among isoforms are not evident in gene-level expression analyses. This is especially relevant for immune genes with similar sequences (e.g., AMPs and PGRPs) that have isoforms with different expression profiles and functions [3, 27, 46].

### Differential expression patterns

**Fig. 1:**
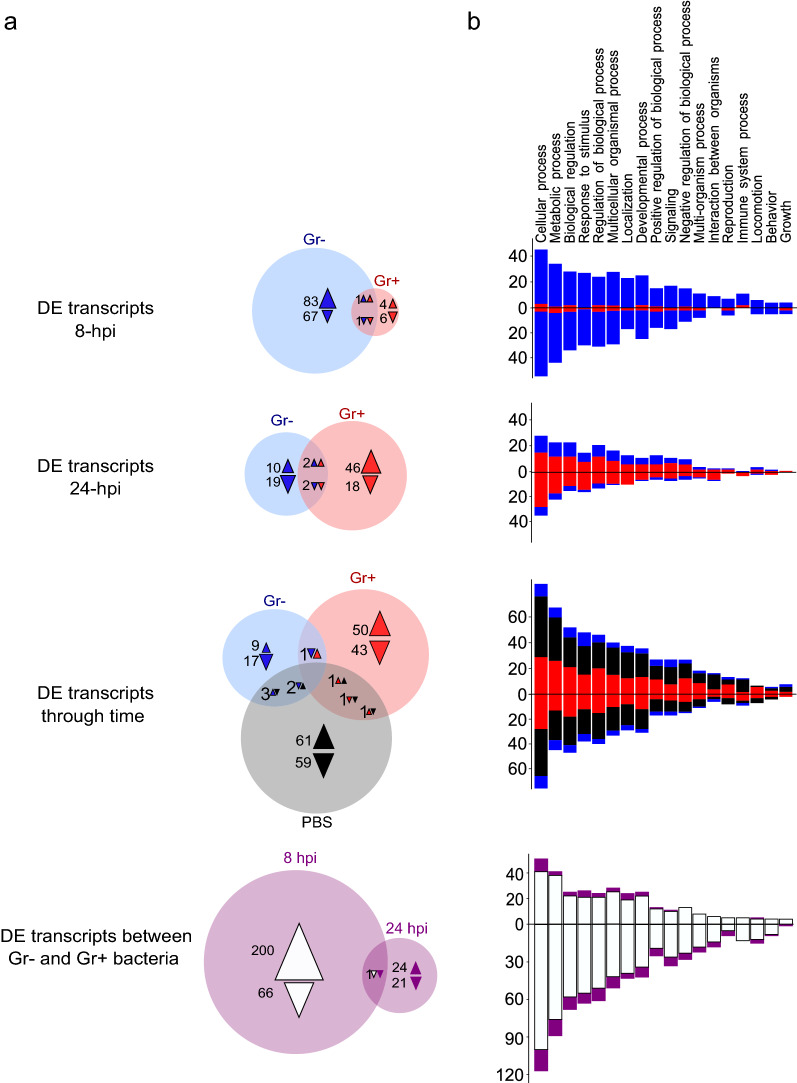
**a**, **b** Differentially expressed (*DE*) transcripts in *Rhodnius prolixus* fat body in response to injections of Gram-negative (Gr-) and Gram-positive (Gr+) bacteria.** a** Venn diagrams of DE transcripts. Numbers within the circles represent the number of DE transcripts with a false discovery rate* P*-value < 0.05; triangles indicate upregulated transcripts, inverted triangles indicate downregulated transcripts. Comparisons of bacteria at 8 (Venn diagram top) and 24 h post-infection (*hpi*) (Venn diagram second from top) use the respective phosphate-buffered saline (*PBS*) time point control as a reference to calculate DE. Comparisons of DE through time (Venn diagram third from top) used 8-hpi time points as a reference for DE estimations. Comparisons between bacterial treatments used the Gr+ bacteria as the reference. **b** Distribution and counts of biological process gene ontology (GO) terms in DE transcripts. Bars are colored according to the respective group in the Venn diagrams, blue for Gr− bacteria, red for Gr+ bacteria, black for PBS, white for comparisons between bacteria 8 hpi, and purple for comparisons between bacteria 24 hpi. Bars above the* x*-axis represent upregulated genes, bars below the *x*-axis represent the number of downregulated genes. Individual transcripts can have multiple GO terms. We display biological process GO terms at level 2 in this figure

A first comparison was done at each time point using the corresponding PBS treatment as a reference. Transcripts with statistically significant differences in expression levels are referred to as DE. Additional file 1: Table S2 contains a list of the immune transcripts included in our analyses; false discovery rate* P*-values for DE immune transcripts are included in Additional file 1: Table S3. Treatments with Gr- bacteria induced more DE transcripts at 8 hpi compared with the PBS control at 8 hpi, while treatments with Gr+ bacteria had more DE transcripts at 24 hpi compared with the PBS control at 24 hpi. In the Gr− bacteria treatment, 152 transcripts were DE at 8 hpi and 33 transcripts were DE at 24 hpi. In the Gr+ bacteria treatment, only 12 transcripts were DE at 8 hpi, while 68 transcripts were DE at 24 hpi (Fig. 1; Additional file 1: Table S1).

A second set of comparisons was done to evaluate changes in transcript expression through time by contrasting the 8-hpi and 24-hpi samples of each treatment. The PBS treatment had the largest number of DE transcripts through time (128 transcripts), and was closely followed by the Gr+ bacteria treatment (97 transcripts). Meanwhile, the Gr- bacteria treatment had very few DE transcripts through time (32 transcripts) (Fig. 1). The high number of DE transcripts in the PBS treatment comparison was unexpected (and was possibly a result of injury due to injection, as discussed in the next sections) (Table 1), potentially masking DE transcripts triggered by the bacteria alone. To account for this possibility, and to highlight specific effects caused by the bacteria, a direct comparison between the Gr− and Gr+ bacteria treatments was done. As our data suggest that the different types of bacteria influence the DE of transcripts at different times, their comparison may allow the identification of more DE transcripts, with the caveat that transcripts affected similarly by both types of bacteria may not be detected. In these analyses, 267 transcripts were DE at 8 hpi and 46 at 24 hpi, and a significantly larger number of DE transcripts were upregulated in the Gr− bacteria treatment compared with the Gr+ bacteria treatment at 8 hpi (Fig. 1). These results further suggest that Gr− bacteria induce changes in immune transcript levels earlier than Gr+ bacteria, and that the changes caused by the injection of Gr− bacteria are maintained for at least 24 hpi.

**Table 1:** Gene ontology (*GO*) term enrichment analysis for biological processes in the fat body (FB) of *Rhodnius prolixus*

Enriched GO term	Fold enrichment per treatment						
	Naïve	PBS (8 hpi)	Gr- (8 hpi)	Gr+ (8 hpi)	PBS (24 hpi)	Gr- (24 hpi)	Gr+ (24 hpi)
Amide biosynthetic process (GO:0043604)	7.71	–	–	–	–	–	–
Biosynthetic process (GO:0009058)	3.56	–	–	–	–	–	–
Cellular amide metabolic process (GO:0043603)	7.13	–	–	–	–	–	–
Cellular biosynthetic process (GO:0044249)	3.81	–	–	–	–	–	–
Cellular nitrogen compound biosynthetic process (GO:0044271)	4.68	–	–	–	–	–	–
Translation (GO:0006412)	8.22	–	–	–	–	–	–
Organic substance biosynthetic process (GO:1901576)	3.69	–	–	–	–	–	–
Organonitrogen compound biosynthetic process (GO:1901566)	5.34	–	–	–	–	–	–
Peptide biosynthetic process (GO:0043043)	8.09	–	–	–	–	–	–
Peptide metabolic process (GO:0006518)	7.77	–	–	–	–	–	–
Defense response (GO:0006952)	50.36	92.33	100.73	89.84	100.73	97.76	94.97
Response to bacteria (GO:0009617, 0042742)	–	174.41	190.26	169.69	190.26	184.67	179.39
Response to stress (GO:0006950)	–	9.07	9.9	8.83	9.9	9.61	9.33
Defense response to other organisms (GO:0098542)	–	87.2	95.13	84.85	95.13	92.33	89.7
Response to biotic stimulus (GO:0009607, 0043207)	–	–	20.39	–	20.39	–	19.22
Response to external stimulus (GO:0009605)	–	–	14.27	–	14.27	–	13.45
Interaction between organisms (GO:0044419)	–	–	17.56	–	17.56	–	16.56

Enriched GO terms were calculated for the six treatments, and data from Ribeiro et al. [38] were used for naïve insect FB tissue. Values represent the fold enrichment of each GO term using as background the *R. prolixus* C3.3 gene set; only values of statistically enriched GO terms are shown (Bonferroni-corrected* P*-values < 0.05). See Table S7 for a complete list of the genes used in this analysis and their corresponding transcripts in the de novo transcriptome presented here

*PBS* Phosphate-buffered saline, *Gr-* Gram negative,* Gr+* Gram positive, *hpi* hours post-infection

### GO terms enrichment analysis of DE transcripts

To better understand the function of these DE transcripts, GO enrichment analyses were done for each of the comparisons described above. Similar proportions of GO terms and categories were found in all DE transcript comparisons (Fig. 1b). This indicates that, globally, the three treatments induced changes in transcript expression levels in similar GO categories. Significantly enriched GO terms were found in transcripts DE by Gr- bacteria at 8 hpi compared with the PBS control (Additional file 1: Table S4). Enriched GO terms from upregulated transcripts included transport of amino acids (arginine and lysine), inosine monophosphate (IMP) metabolism, Toll pathway positive regulation, and organ development [30, 66–70]. Enriched GO terms in downregulated transcripts included IMP metabolism, nucleotide and nucleoside metabolism, Toll pathway negative regulation, melanin metabolism, regulation of immune processes, and phenolic compound metabolism. Several of these transcripts are related to the melanization, Toll, and IMD pathways, and are discussed further below. GO terms were also significantly enriched in the PBS treatment over time. Enriched GO terms in upregulated transcripts at 24 hpi were related to IMP, nucleotide, and nucleoside metabolism. Enriched GO terms in downregulated transcripts at 24 hpi were mostly related to phenolic compound metabolism, regulators of immune responses, Toll pathway negative regulators, and organ development. These results suggest that the PBS treatment induces various immune responses through time and that these responses are similar to the changes caused by Gr− bacteria at 8 hpi. In other comparisons, no enriched GO terms were detected.

### In silico evaluation of immune responses associated with PBS injection

The identified enriched GO terms in the PBS treatments indicate that the wound inflicted by the PBS injection may have activated immune responses. This is further supported by our failure to detect a strong increment in AMP transcript levels typical of bacterial infections in *R. prolixus* (See below). To test this hypothesis, and due to a lack of a control group of unexposed insects, we compared the 50 most highly expressed genes from our treatments with the 50 most highly expressed genes in unstimulated insects (naïve) reported by Ribeiro et al. [38] (Additional file 1: Table S7). To enable comparison of our data with those of Ribeiro et al. [38], we mapped our reads to the *R. prolixus* v. 4.3 genome assembly and measured expression at the gene level. The analysis of highly expressed genes in transcriptomes is commonly used to identify the prevailing function of a tissue or organ. The two main functions of the FB in insects are the metabolism of biomolecules and the control of immune responses. We hypothesized that wounded or infected insects would have a larger proportion of immune genes rated as highly expressed compared with naïve insects. As predicted, in the naïve insects, only four immune genes were among the top 50 most highly expressed genes (Additional file 1: Table S7), while most of the remaining genes are part of metabolic pathways (Table 1; Additional file 1: Table S7). These results are supported by the overrepresentation of GO terms related to metabolic processes but not immune processes in this naïve group (Table 1). In contrast, in the six treatments, we found 12–14 genes with immune functions among the 50 most highly expressed genes. Many of these highly expressed immune genes are effector AMPs, including prolixicin (which was consistently the most expressed gene in all samples), defensins, a lysozyme, and an attacin. All of these highly expressed genes were recovered in our de novo transcriptome (Additional file 1: Tables S4, S7). We found that 36 of these genes have DE transcripts in our transcriptome, mostly with predicted immune function (Additional file 1: Table S7). The purported immune activation in our samples was further supported as only overrepresented immune GO terms were detected (Table 1; Additional file 1: Table S7). It is important to note that the data set from Ribeiro et al. [38] was obtained using adult female insects under different physiological conditions than the insects used in our transcriptome. These confounding factors may also explain the transcriptional and functional differences detected between the present study and Ribeiro et al.’s [38].

### DE immune-related transcripts

To further study the immune responses triggered by bacteria in *R. prolixus*, we analyzed a list of manually curated gene transcripts that were identified in our annotation as having immune functions.

**Fig. 2:**
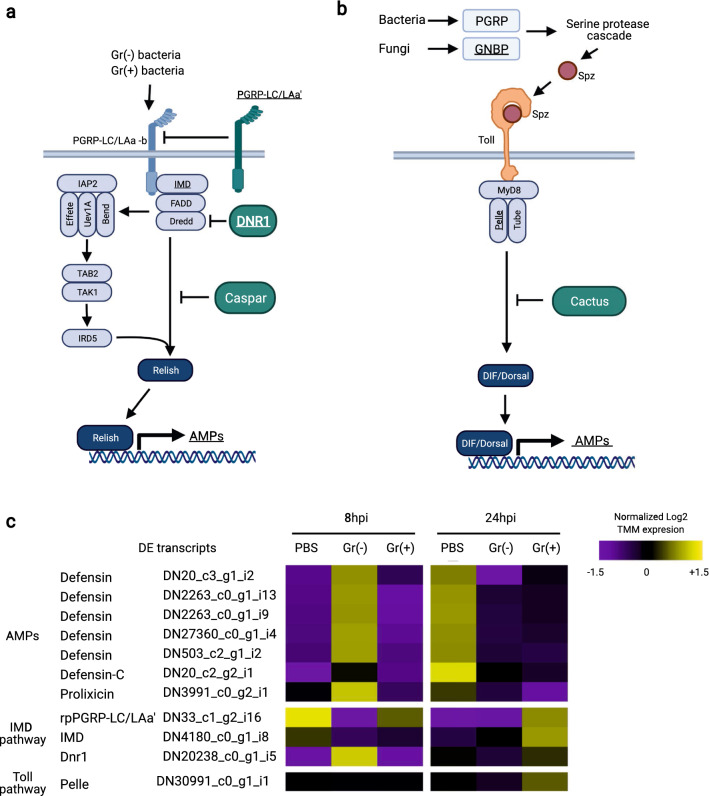
**a**–**c** DE transcripts of the immune deficiency (*IMD*) and Toll pathways of *Rhodnius prolixus* in response to bacterial injections. Diagrams represent **a** IMD and **b** Toll pathway models in *R. prolixus*. Arrows represent the activation while blunt-ended lines represent the suppression of processes or molecules. Underscored and bolded molecules are transcripts with differential expression in at least one comparison between two treatments. The molecules marked with an asterisk have not been studied in triatomines. **c** Heat maps showing DE transcripts from the IMD and Toll pathways 8 hpi and 24 hpi with PBS, Gr− or Gr+ bacteria. Accession numbers are provided for annotated genes. The color of each cell indicates expression level. Displayed transcript expression values are the log 2 trimmed mean of M values (*TMM*) average of the replicates of each treatment divided by the average TMM expression of all treatments. For raw TMM values, see Additional file 1. For other abbreviations, see Fig. 1

AMPs: Previous studies on *R. prolixus* reported a large increase in AMP transcripts in the FB after bacterial challenge [3, 26, 27, 46, 47, 71]. In contrast, for our samples, very few comparisons showed differntial expression of AMP transcripts (Fig. 2a; Additional file 1: Tables S2, S3). Changes in AMP transcript levels through time showed that defensin C (DN20_c2_g2_i1) was upregulated in the PBS treatment at 24 hpi compared with the PBS treatment at 8 hpi (log 2FC = 3.05). To account for the potential wound effects of the PBS injection, we compared AMP expression levels between the Gr- and Gr+ bacterial treatments. At the 8 hpi time point, the Gr- treatment upregulated the expression of multiple defensins and prolixicin (Fig. 2c; Additional file 1: Table S3), the latter of which is controlled by the IMD pathway (log 2FC between 1.70 and 2.28). Unexpectedly, transcripts for defensin A, defensin B, lysozyme A, and lysozyme B, usually induced by bacterial infections and controlled by the IMD pathway [3, 26, 27], were not DE (Additional file 1: Table S2). Defensin C, which is not regulated by the IMD pathway in the FB [26, 27], was not DE by the bacterial treatments.

IMD pathway: We analyzed DE changes from IMD pathway gene transcripts (Fig. 2b; Additional file 1: Table S1). At the receptor level, the PRR PGRP-LC/LAa’ (DN33_c1_g2_i16), predicted to act as a negative regulator of the IMD pathway [27], was suppressed in the Gr- bacteria treatment compared with the PBS 8 hpi and Gr+ bacteria treatments 8 hpi (log 2FC = −11.70 and −12.66). The expression of this transcript was similarly downregulated in the PBS treatment at the 24-hpi time point compared with the PBS treatment at the 8-hpi time point (log 2FC = −12.65). The downregulation of PGRP-LC/LAa’ may be related to the activation of the IMD pathway during Gr− bacteria infections 8 hpi, as this downregulation coincided with slightly higher levels of IMD pathway-regulated AMPs in the PBS samples at 24 hpi and the Gr- bacteria samples at 8 hpi (Fig. 2). At 24 hpi, PGRP-LC/LAa’ was upregulated in the Gr+ bacteria treatment compared with the PBS control treatment (log 2FC = 12.15), which coincided with a lower expression of AMP transcripts (Fig. 2). An alternative interpretation of these data is that PGRP-LC/LAa’ is induced by the PBS injection 8 hpi and by Gr+ at both time points. However, we believe that this last explanation is unlikely, as few immune gene transcripts were induced by Gr+ bacteria 8 hpi. Furthermore, many transcripts were DE at 8 hpi in the Gr− treatment, and many of these were also DE in the PBS treatment at 24 hpi. Another putative negative regulator of the IMD pathway, Dnr1 (DN20238_c0_g1_i5) [72, 73], was upregulated 8 hpi by the Gr− bacteria treatment compared with the PBS control at the same time point (log 2FC = 2.55). This change in expression should downregulate the IMD pathway; however, no changes in the AMP levels matched this prediction. It is important to note that neither PGRP-LC/LAa’ nor Dnr1 have been functionally assessed in *R. prolixus*. Finally, a putative IMD ortholog (DN4180_c0_g1_i8) was upregulated by the Gr+ bacteria treatment 24 hpi compared with the PBS control at the same time point (log 2FC = 1.71). Although the IMD pathway has been associated with responses to Gr- bacteria in most insects, some AMPs upregulated in *R. prolixus* by the IMD pathway in response to Gr- bacteria at 8 hpi are also induced by Gr+ bacteria at 24 hpi [26, 27]. The late upregulation of the IMD pathway might explain these results. However, the mechanisms by which Gr+ bacteria activate this pathway are still unknown. Other gene transcripts in the IMD pathway showed small and non-significant expression level changes.

Toll pathway: The activation of the Toll pathway in insects starts extracellularly and involves GNBP, PGRPs, and the activation of SP cascades [25]. In our transcriptome, we detected three DE GNBP transcripts (two from one gene and the the other one from another gene) (Fig. 2c; Additional file 1: Table S1) with variable DE patterns. The expression of GNBP1a (DN1774_c0_g1_i15) was reduced in the PBS treatment at 24 hpi compared with the PBS control at 8 hpi (log 2FC = − 8.13). GNBP1b (DN1774_c0_g1_i6) expression was downregulated in the Gr- bacteria 8 hpi compared with the Gr- bacteria 8 hpi treatment (log 2FC = − 1.60) and with the PBS 8 hpi control (log 2FC = −2.42). The expression of GNBP1b was upregulated in the Gr+ bacteria treatment compared with the PBS control at 24 hpi (log 2FC = 2.04). GNBP2 (DN2658_c0_g1_i16) expression was reduced in the Gr- bacteria treatments 8 hpi and PBS treatments 24 hpi compared with the PBS control at 8 hpi (log 2FC = -− 2.50 and − 2.50, respectively). Finally, expression of GNBP2 was increased in the Gr+ bacteria treatment 24 hpi compared with the PBS control 24 hpi (log 2FC = 2.62). These results suggest that the treatments with PBS 8 hpi and Gr+ bacteria 24 hpi activated the transcription of GNBPs, or the treatments with Gr- bacteria 8 hpi and PBS 24 hpi reduced the expression of GNBPs. These specific effects may be the result of the simultaneous recognition of multiple DAMPs and PAMPs in each treatment. An analysis of the transcript levels of intracellular Toll pathway genes showed small and statistically non-significant changes in the levels of expression. Only Pelle (DN30991_c0_g1_i1) was overexpressed in insects 24 hpi compared with 8 hpi when they were challenged with Gr+ bacteria (log 2FC = 1.33) (Fig. 2c). This result further suggests that the Toll pathway is activated by Gr+ bacteria 24 hpi.

**Fig. 3:**
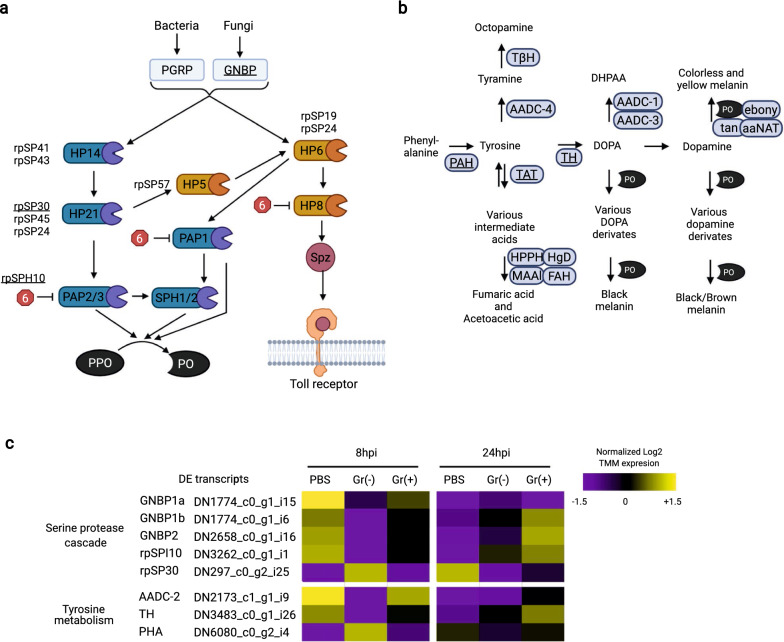
**a**, **b** Differentially expressed (DE) transcripts of the immune serine protease (SP) cascade and tyrosine metabolism pathway of *Rhodnius prolixus* in response to bacterial injection. **a** Diagram representing the immune SP cascade of* Manduca sexta* (modified from An et al. [99] and Wang et al. [34]). Putative orthologs of identified SPs and SP inhibitors (SPIs) in* R. prolixus* are listed next to the* M. sexta* proteins. SPIs are represented as red hexagons next to the SPs they inhibit. Only SPI transcripts with identified orthologs in* R. prolixus* are displayed. **b** Diagram representing a simplified tyrosine metabolism pathway in* R. prolixus* (modified from Sterkel et al. [100]). Arrows represent the activation of processes or molecules. Underscored and bolded molecules are transcripts with differential expression in at least one comparison between two treatments.** c** Heat maps showing DE molecules from the immune SP cascade and tyrosine metabolism pathways in* R. prolixus* 8 and 24 hpi with PBS, Gr−, or Gr+ bacteria. Accession numbers are provided for annotated genes. The color of each cell corresponds to expression level. Displayed transcript expression values are the log 2 TMM average of the replicates of each treatment divided by the average TMM expression of all treatments. For raw TMM values, see Additional file 1. For other abbreviations, see Figs. 1 and 2

SPs and inhibitors: SPs and SPIs are involved in the extracellular activation of the Toll and melanization pathways, and have been well studied in some holometabolous insects [5, 29–34, 74]. However, they have not been characterized in triatomines. To identify SPs and SPIs potentially involved in immune responses in *R. prolixus*, we built phylogenetic trees that included SPs and SPIs from our transcriptome (Figs. S1 and S2; Additional file 1: Tables S5, S6) and evaluated their DE patterns. We identified 66 SP and assigned a unique name to each of them; nine of these have CLIP domains commonly found in immune SPs of other insects. Based on our phylogenetic analyses, we predicted that some of these SPs are likely to be part of the cascade that activates the Toll or the melanization pathway in *R. prolixus* (Fig. 3a). Only SP rpSP30 (DN297_c0_g2_i11) was DE. This SP clusters with SPs from *M. sexta* (HP21) [75] and *T. molitor* (SAE) [5] (Additional file 1: Fig. S1), which are essential for the activation of these insects’ Toll and melanization pathways. Two other SP identified in our transcriptome [rpSP24 (DN2264_c0_g1_i7) and rpSP45 (DN402_c1_g1_i1)] also clustered with HP14 and SAE. These might be potential orthologs, but they were not DE according to any of the comparisons (Additional file 1: Fig. S1). In our transcriptome, rpSP30 was induced by Gr− bacteria 8 hpi (log 2FC = 3.22) and PBS 24 hpi (log FC = 2.48) compared with the PBS control at 8 hpi. rpSP30 was also overexpressed in the Gr- bacteria treatment 8 hpi compared with the Gr+ bacteria treatment at the same time point (log 2FC = 2.61). In a similar analysis, where we identified 14 SPI genes encoding 24 transcripts, only rpSPI10 (DN3262_c0_g1_i1/i3/i5) transcripts were DE. This SPI is similar to SPIs from *M. sexta* (serpin6) [76] and *D. melanogaster* (serpin 88Ea) [77] that inhibit the activation of their Toll and melanization pathways (Additional file 1: Fig. S2). rpSPI10 transcripts were suppressed in the Gr− bacteria treatment 8 hpi (log 2FC reduction of between − 3.55 and − 3.59) and the PBS treatment 24 hpi (log 2FC reduction of between -3.42 and -3.61) compared with the PBS control at 8 hpi. These changes in rpSP30 and rpSPI10 transcript levels suggest the activation of the SP cascade by Gr− bacteria at 8 hpi and by wounding at 24 hpi. These results are, however, unexpected, as Gr- bacteria are not detected in most insects by PRRs of the Toll pathway. Instead, Gr+ bacteria and fungi are known to activate these SP cascades in most insects. A potential explanation for these changes is that only the melanization response is activated by Gr- bacteria and wounding. This is known to be the case in other insects in response to wounding signals and by the action of bacterial SPs that activate this cascade [78].

Melanization: One of the immune responses activated by the SP cascade is the melanization process. This response involves multiple enzymes that transform tyrosine and tyrosine derivates into melanin (Fig. 3b). In our transcriptome, three of these enzymes were DE. At 8 hpi, infection with Gr− bacteria increased the expression of phenylalanine hydroxylase (DN6080_c0_g2_i4 ) (log 2FC = 2.53) and reduced the levels of tyrosine hydroxylase (log 2FC = − 2.90) and aromatic amino acid decarboxylase (AAD 2 (DN2173_c1_g1_i9) (log 2FC = 6.50) compared with the PBS control. These changes may favor the synthesis of tyrosine but lead to a reduction in the production of other substrates needed for the production of melanin. One possibility is that these expression changes counteract the activation of the SP cascade by Gr− bacteria 8 hpi. At the 24-hpi time point, both types of bacteria induce AADC-2. The levels of this transcript at 24 hpi in the Gr− bacteria treatment were 3.68 and 3.14 log 2 times higher, respectively, when compared with the PBS controls at 8 hpi and 24 hpi. Similarly, the levels of AADC-2 in the G+ bacteria treatment were 5.15 and 5.95 log 2 times higher, respectively, than in the PBS controls at 8 hpi and 24 hpi. The upregulation of AADC-2 at 24 hpi should favor the production of dopamine, which is used as a substrate for the production of melanin. These changes in AADC-2 expression levels might be a response to re-establish levels of substrates that are used in the activation of the melanization pathway 8 hpi.

## Discussion

The FB is a multipurpose organ in insects [79–81]. Some of its functions are related to nutrient storage, the synthesis and transport of biomolecules, vitellogenesis, and the detoxification of dietary derivatives. The FB is also the main immune organ in insects, producing AMPs and regulating cellular and humoral immune responses. The precise regulation of these different processes is complex, and essential, and involves dozens of pathways [80]. Transcriptomic analyses are an ideal approach to deducing how these processes are regulated, and hence have been used extensively in the study of model holometabolous insects such as *D. melanogaster* and *M. sexta* [82, 83]. However, many of these processes have not been as well studied in hemimetabolous insects. In triatomines, for instance, only a few FB transcriptomes have been created, mainly for the study of the metabolic and endocrine functions of FB tissue [44, 45, 84]. In this study, we explored the antimicrobial immune function of the FB in triatomines by characterizing the transcriptome of *R. prolixus* in response to infection with Gr- and Gr+ bacteria.

We found that the Gr- and Gr+ bacteria induced time-specific immune responses. In our assays, Gr- bacteria caused the DE of a large number of transcripts at 8 hpi, while Gr+ bacteria induced the DE of many transcripts at 24 hpi. In insects, specific immune responses are triggered by Gr- and Gr+ bacteria which are based on the recognition of unique PAMPs for each bacterial type. However, delayed or time-dependent activation of immune responses to different types of bacteria has not been reported. A slower response to Gr+ bacteria compared with Gr- bacteria in insects is not typical, as immune responses should be mounted rapidly to control pathogens. Some bacteria and fungi have evasion mechanisms that prevent their immediate detection, or they may suppress the host immune system, causing delayed immune responses [85–87]. We do not, however, believe that this can explain our results, as in previous studies on *R. prolixus*, in which we used other Gr+ and Gr- bacteria, we also found slower activation of immune pathways in infections with Gr+ compared with Gr- bacteria [26, 27]. It is also possible that triatomine immune responses that do not need fast transcriptional modulation, such as phagocytosis and melanization, are used earlier in the control of bacteria, and then transcriptionally modulated responses are activated if deemed necessary. Additional studies with heat-inactivated bacteria, different bacterial doses, and the measurement of bacterial elimination might help to elucidate whether the insect or the bacteria are causing these time-dependent responses.

The wound inflicted by the injection of our samples may have triggered immune responses similar to those mounted towards bacteria, preventing the detection of DE of AMPs. Immune responses associated with sterile wounding have been reported in *D. melanogaster* [29, 88, 89]. Injury in *D. melanogaster* releases host DAMPs that activate SP cascades, the Toll, Janus kinase-signal transducer and activator of transcription, JNK, and IMD pathways, and the production of AMPs [29, 88, 89]. The use of a common set of pathways in wound and immune responses is common to most organisms and is explained by the danger theory, according to which organisms sense “danger” signals (i.e., PAMPs and DAMPs) that activate similar responses and pathways [89, 90] and may explain the high level of AMP transcripts in our PBS-injected insects. Some of the responses seen in our samples may also be the result of systemic immune activation associated with DAMPs from the wound or the damage caused by the bacteria. Systemic immune responses in insects occur when an infected or damaged tissue increases the immune activity of non-infected or damaged tissues. For instance, a species of *Plasmodium* infecting the gut of a species of *Anopheles* induced immune responses in the FB in the absence of an infection in the hemocoel [91]. Similarly, feeding triatomines bacteria or *T. cruzi* may affect AMP transcript expression in the FB [92–94]. This systemic immune response may protect other tissues from potential pathogens [91]. Many of these systemic responses are believed to be caused by DAMPs. DAMPS in insects are small molecules that can diffuse through tissues (actin, H_2_0_2_, NO, collagen, small peptides). However, detailed studies on wound and DAMP responses have not been undertaken for triatomines [95].

Our data suggest that the PBS injection affected the expression of immune transcripts for multiple reasons. First, very few transcripts were DE between the bacteria-treated insects and the PBS-injected insects. Secondly, most enriched GO terms were common between the PBS and the Gr- bacteria treatments at 24 hpi. Thirdly, in all our samples, genes with immune functions are among the most highly expressed. This was not the case in a study of naïve *R. prolixus* by Ribeiro et al. [38], in which genes involved in metabolic and endocrine processes were among the most highly expressed genes. Fourthly, our bacterial injections contained a considerable quantity of bacteria that were not present in the PBS injections. Altogether these results indicate that the wound caused by the injection induced responses that overlap with those triggered by bacteria, and also serve to support the danger theory for triatomines. These responses may involve DAMPs and systemic immune responses.

The high number of DE transcripts seen in the comparisons through time of the PBS-injected samples is potentially a result of having only one factor responsible for these differences (i.e., time after injection), which facilitates the detection of DE transcripts. In other treatment comparisons, however, multiple factors influence the levels of expression (i.e., responses to the bacteria, responses to the wound, and also time after injection). The potential interactions of these factors and their effect on immune pathways may have increased the amount of stochasticity and biological variability, making the detection of DE transcripts in these comparisons more difficult.

A major limitation of this study is the lack of a control group of insects not exposed to bacteria (i.e., naïve insects). We used the FB transcriptome described by Ribeiro et al. [38] to compare and contrast our results. In Ribeiro et al.’s [38] study, a FB transcriptome was constructed from insects not exposed to bacteria. There are some caveats, however, regarding the comparison of these two transcriptomes. For instance, in these studies, the insects were at different developmental stages and experiencing different physiological conditions when sampled, and different sequencing methodologies were used (fifth instar insects were injected in the present study and female insects were blood-fed in Ribeiro et al. [38]). Despite these differences, contrasting functional profiles are evident between Ribeiro et al.’s [38] samples and ours. This suggests that the PBS injections activated immune responses that may overlap with some of the immune responses caused by the bacterial injections. However, the lack of a control group of naïve insects prevents us from confidently concluding that this is correct.

Despite the potential effects of the wound, we detected specific and contrasting responses to the two bacterial treatments. The comparison of the two allowed us to better account for the unexpected effects of the PBS injections, and to detect changes in the expression of other immune transcripts. In this comparison, some AMPs, regulated largely by the IMD pathway, were upregulated by Gr- bacteria. Similarly, many AMPs were among the most highly expressed transcripts in all our samples, and were potentially induced by the PBS injection. We believe this to be true, as AMP levels have been consistently reported to be strongly induced by bacterial challenge in *R. prolixus*. Defensin C had contrasting patterns of expression compared with other AMPs, suggesting it has a different regulation mode. Previous studies on *R. prolixus* indicate that defensin C has an immune function, principally in the midgut, and helps to regulate intestinal bacterial populations, which explains its considerably lower expression in the FB than the other AMPs evaluated [92–94]. Further studies on the regulation of this gene would help explain how tissue-specific antimicrobial responses are regulated in triatomines. While there is some basal constitutive expression of AMPs, most are strongly induced when pathogens are detected. Previous studies on triatomine immune responses found strong induction of AMPs in response to bacterial infections compared with insects wounded under sterile conditions [3, 26, 27, 46, 47, 71]. However, we did not detect similar increments in AMP transcript levels in our assays. These contrasting results might be explained by differences in the methods used to inoculate the bacteria [26, 27, 46, 47, 71]. In this study, we injected bacteria using a Hamilton syringe, while other studies used thin entomological pins dipped into a bacterial pellet to inoculate the insects, creating a significantly smaller wound compared with our approach [26, 27, 46, 47, 71]. Those studies also used quantitative PCR to compare the differential expression of specific genes, which is a more sensitive technique than transcriptomics for the detection of differences in transcript expression.

Our analysis of the IMD pathway revealed that only two putative negative regulators of this pathway (PGRP-LC/LAa’ and Dnr1) were DE. PGRP-LC/LAa’ was strongly downregulated in treatments with Gr- bacteria 8 hpi and PBS 24 hpi; these changes were associated with higher AMP transcript levels, supporting the purported role of PGRP-LC/LAa’ as a negative regulator. The upregulation of Dnr1 in the Gr- bacteria 8 hpi, however, was not associated with lower levels of AMP expression. In *D. melanogaster*, PGRP-LF acts as a negative regulator of the IMD pathway by blocking the bacterial recognition groove in the receptor PGRP-LC [96]. Silencing the gene that encodes this protein (as well as* Dnr1*) caused an increase in the levels of AMPs controlled by the IMD pathway in *D. melanogaster* [72, 73, 96]. This mechanism may also be used by PGRP-LC/LAa’ in *R. prolixus*. It is possible that this mechanism is present in *D. melanogaster* and triatomines through heredity from a common ancestor. An alternative explanation is that this mechanism exists in both insects due to functional convergence; the PGRP repertoire and their sequences are quite different in these two organisms, and evolutionary explanations should be conservative [97].

In most insects, the Toll pathway is typically reported to regulate responses towards Gr+ bacteria and fungi [17]. This is done by using a different set of PRRs than those used in the IMD pathway, including GNBPs. These PRRs have not been characterized in triatomines. In our analysis of the Toll pathway, we found three GNBP transcripts that may play this role, as they were DE in the Gr+ bacteria treatment at 24 hpi. In other insects, GNBPs also can induce melanization in parallel with Toll pathway activation [30, 33, 98, 99]. This occurs by the differential activation of SPs that eventually cleave Spätzle in the Toll pathway or PPO in the melanotic biochemical pathway. Melanization processes in insects are involved in developmental and immune processes [30, 66–70, 100]. In triatomines, the former function has been extensively studied [100], but the immune SP cascade prior to the cleavage of PPO has not been characterized. By comparing our transcriptome to the well-characterized melanization pathways reported for *M. sexta*, *D. melanogaster*, and *T. molitor* [32–34, 98, 99, 101–103], we were able to identify SPs and SPIs, and created a model of the SP cascade that may operate in triatomines. A model of an immune-related SP cascade in triatomines could be useful for the study of the SP cascades in other hemimetabolous insects, as these have not been studied functionally in these insects. In our transcriptome, we found that rpSP20, rpSPI10, and some genes from the tyrosine metabolic pathway were DE in the Gr- bacteria and PBS treatments. In other insects, it has been shown that damage responses activate the melanization response as a mechanism of wound repair [32, 78]. It is possible that the melanization responses in triatomines are activated by damage signals from wounds and tissue damaged by Gr- bacteria. The absence of this response in the insects injected with Gr+ bacteria is, however, puzzling. One plausible explanation is that the Gr- bacteria used in our experiments can directly cleave SPs and activate the SP cascade without the involvement of PRRs. Some Gr+ and fungi are known to have such proteases, and it has been suggested that some Gr- bacteria can also activate the SP cascade using proteases [79, 104, 105]. The previously proposed mechanisms of immune activation associated with wounding and bacterial infection remain to be confirmed experimentally.

## Conclusions

We analyzed the immune role of the FB of triatomines in response to the injection of Gr- and Gr+ bacteria. We found that immune responses to Gr- and Gr+ bacteria are time dependent and specific to each type of bacteria. Some immune pathways are activated primarily by either Gr- or Gr+ bacteria, but many responses are activated by both. Although our original aim was to study only the responses elicited by bacteria, we found that the wound created by injection also activates immune responses similar to those used against bacteria. These wound responses limited our ability to detect DE of transcripts exclusively caused by bacteria, but help to support the danger theory with respect to triatomines. Among the DE transcripts, we found novel genes from understudied pathways, including PRRs from the Toll pathway and SPs and SPIs that are part of the Toll and melanization pathways. Taken together, these results improve our understanding of triatomine immune responses. The findings presented here add to the already extensive body of knowledge on the physiology of *R. prolixus*, and when combined are useful for the study of the evolution of immune responses and gene regulation in triatomines and other hemimetabolous insects.

## Supplementary Information

**Additional file 1: Table S1.** Differentially expressed (DE) transcripts in the fat body (FB) tissue of *Rhodnius prolixus* for multiple pairwise comparisons. These values were used to create Fig. 1. Log 2 fold change values were calculated for each comparison using the data above each row as the reference value. Empty cells indicate transcripts that were not statistically DE. **Table S2.** List of *Rhodnius prolixus* immune transcripts used for differential expression analyses. Transcripts with known or predicted immune functions are listed and categorized according to their function or molecular pathway. Expression values used to create heatmaps of Figs. 2, 3 are listed at the bottom of the table. **Table S3.** DE immune transcripts in the FB of *Rhodnius prolixus.* Immune transcripts with DE are listed, log 2 fold change values were calculated using as a baseline the reference condition values. **Table S4.** Statistically significant enriched gene ontology (GO) terms from pairwise comparisons. Enriched GO terms were found for the Gr- bacteria and PBS treatments at 8 hpi and 24 hpi, respectively, when compared with the PBS treatment at 8 hpi, but not for the other comparisons.* BP* Biological process,* MF* molecular function,* CC* cellular compartment. **Figure S1.** Maximum likelihood phylogenetic tree of serine proteases (*SP*) from selected insects. Multiple SP clades containing SPs from different species were formed. *Rhodnius prolixus* SPs (blue) are distributed across the tree; some *R. prolixus* SP are clustered together with SP from *Manduca sexta* (green) that participate in the Toll and melanization pathways. Some clades including multiple species are collapsed for display purposes. Clade support is shown as percentage values of 1000 ultrafast bootstrap replicates. **Figure S2.** Maximum likelihood phylogenetic tree of SP inhibitors (*SPI*) from selected insects. Only a few SPI from *Rhodnius prolixus* (blue) are clustered together with SP from *Manduca sexta* (green) that participate in the Toll and melanization pathways. Some clades including multiple species are collapsed for display purposes. Clade support is shown as percentage values of 1000 ultrafast bootstrap replicates. **Table S5.** *Rhodnius prolixus* SPs. List of SPs identified in a *Rhodnius prolixus* FB tissue transcriptome. The closest ortholog to these SPs was identified in *Drosophila melanogaster* and *Manduca sexta* using phylogenetic analyses from Additional file 1: Figure S1. **Table S6.** *Rhodnius prolixus* SPIs. List of SPIs identified in a *R. prolixus* FB tissue transcriptome. The closest ortholog to these SPIs was identified in *Drosophila melanogaster* and *Manduca sexta* using phylogenetic analyses from Additional file 1: Figure S2. **Table S7.** *Rhodnius prolixus* top 50 most highly expressed genes in the FB. A list of highly expressed genes was generated from the six treatments used in the construction of a FB transcriptome and the data set of naïve insects from Ribeiro et al. [38]. All these genes have corresponding sequences in the de novo transcriptome. DE transcripts are highlighted (grey background) by using the information from Table S1.* Gr-* Gram-negative bacteria,* Gr+* Gram-positive bacteria,* hpi* hours post-injection. **Table S8.** List of SPs and SPIs used for the construction of the phylogenetic trees depicted in Additional file 1: Figures S1 and S2.

## Data Availability

Data supporting the findings of this work are presented in the supplementary files. The raw Illumina reads used to build the transcriptome and the final assembled transcriptome are available from the National Center for Biotechnology Information SRA under the BioProject accession number PRJNA755997.

## Abbreviations

AADC: Aromatic amino acid decarboxylase
AMP: Antimicrobial peptide
DAMP: Damage-associated molecular pattern
DE: Differentially expressed
FB: Fat body
GNBP: Gram-negative binding proteins
GO: Gene ontology
Gr-: Gram-negative
Gr+: Gram-positive
hpi: Hours post-injection
IMD: Immune deficiency
IMP: Inosine monophosphate
Log 2FC: Log 2 fold change
PAMP: Pathogen-associated molecular pattern
PBS: Phosphate-buffered saline
PGN: Peptidoglycan
PGRP: Peptidoglycan recognition proteins
PPO: Prophenoloxidase
PRR: Pattern recognition receptor
SP: Serine protease
SPI: Serine protease inhibitor
