# Supplementary material for: A fat body transcriptome analysis of the immune responses of Rhodnius prolixus to artificial infections with bacteria
Source: Parasit Vectors. 2022 Jul 29;15:269. doi: 10.1186/s13071-022-05358-9 (PMC9335980; doi:10.1186/s13071-022-05358-9)
Supplement: Supplementary file 1 — Additional file 1: Table S1. Differentially expressed (DE) transcripts in the fat body (FB) tissue of Rhodnius prolixus for multiple pairwise comparisons. These values were used to create Fig. 1. Log 2 fold change values were calculated for each comparison using the data above each row as the reference value. Empty cells indicate transcripts that were not statistically DE. Table S2. List of Rhodnius prolixus immune transcripts used for differential expression analyses. Transcripts with known or predicted immune functions are listed and categorized according to their function or molecular pathway. Expression values used to create heatmaps of Figs. 2, 3 are listed at the bottom of the table. Table S3. DE immune transcripts in the FB of Rhodnius prolixus. Immune transcripts with DE are listed, log 2 fold change values were calculated using as a baseline the reference condition values. Table S4. Statistically significant enriched gene ontology (GO) terms from pairwise comparisons. Enriched GO terms were found for the Gr- bacteria and PBS treatments at 8 hpi and 24 hpi, respectively, when compared with the PBS treatment at 8 hpi, but not for the other comparisons. BP Biological process, MF molecular function, CC cellular compartment. Figure S1. Maximum likelihood phylogenetic tree of serine proteases (SP) from selected insects. Multiple SP clades containing SPs from different species were formed. Rhodnius prolixus SPs (blue) are distributed across the tree; some R. prolixus SP are clustered together with SP from Manduca sexta (green) that participate in the Toll and melanization pathways. Some clades including multiple species are collapsed for display purposes. Clade support is shown as percentage values of 1000 ultrafast bootstrap replicates. Figure S2. Maximum likelihood phylogenetic tree of SP inhibitors (SPI) from selected insects. Only a few SPI from Rhodnius prolixus (blue) are clustered together with SP from Manduca sexta (green) that participate in the Toll and [file 13071_2022_5358_MOESM1_ESM.docx]

Table S1. Differentially expressed (DE) transcripts in the fat body tissue of *Rhodnius prolixus* for multiple pairwise comparisons. These values were used to create Fig 1. Log2 Fold change values were calculated for each comparison using the treatment on top of each columns was used as the reference treatment.

| ID | Annotation | PBS.8h Ec.8h | PBS.8h Sa.8h | Ec.8h Sa.8h | PBS.24h Ec.24h | PBS.24h Sa.24h | Ec.24h Sa.24h | Ec.8h Ec.24h | Sa.8h Sa.24h | PBS.8h PBS.24h |
| --- | --- | --- | --- | --- | --- | --- | --- | --- | --- | --- |
| DN2662_c0_g1_i12 | Activating_transcription_factor_of_chaperone | -14.2 | - | - | - | - | -15.23 | 14.13 | - | - |
| DN1944_c1_g1_i2 |  | -12.47 | - | - | - | - | - | - | - | - |
| DN297_c0_g2_i5 | rpSP30 | -12.16 | - | - | 9.9 | 7.87 | - | 10.29 | - | -11.78 |
| DN4650_c0_g2_i2 |  | -11.56 | - | - | - | - | - | - | - | - |
| DN6977_c0_g1_i2 | Protein_60A | -12.28 | -13.17 | - | - | - | - | 12.22 | - | - |
| DN8382_c0_g1_i5 |  | -11.33 | - | 4.92 | - | - | - | - | - | - |
| DN4188_c0_g1_i3 | Facilitated_trehalose_transporter_Tret1 | -12.59 | - | - | - | - | - | - | - | - |
| DN33_c1_g2_i16 | rpRPRC-LC/LAa' | 12.66 | - | -11.7 | - | -12.15 | - | - | - | 12.65 |
| DN1135_c0_g1_i2 |  | -8.84 | - | - | - | - | - | - | - | -8.58 |
| DN2148_c0_g1_i2 |  | 11.24 | 11.41 | - | - | -10.97 | - | - | -11.15 | 11.23 |
| DN2662_c0_g1_i1 | Activating_transcription_factor_of_chaperone | -11.42 | - | - | - | - | -12.32 | 11.36 | - | - |
| DN16774_c0_g1_i3 | ATP-binding_cassette_sub-family_G_member_1 | -11.84 | - | - | 3.17 | - | - | - | - | -11.92 |
| DN1019_c0_g1_i7 |  | -11.87 | - | - | - | - | - | - | - | - |
| DN105_c0_g3_i1 | Pancreatic_lipase-related_protein_2 | 6.4 | - | - | - | - | - | - | - | 5.76 |
| DN5199_c0_g1_i2 |  | 11.04 | - | - | - | - | - | - | - | 11.03 |
| DN2173_c1_g1_i4 | Aromatic-L-amino-acid_decarboxylase | 5.44 | - | - | - | - | - | - | - | 5.36 |
| DN1006_c0_g1_i11 |  | 8.71 | - | - | - | - | - | - | - | 8.39 |
| DN2173_c1_g1_i9 | Aromatic-L-amino-acid_decarboxylase (AADC-2) | 6.49 | - | - | -3.15 | -5.15 | - | -3.69 | - | 5.94 |
| DN2662_c0_g1_i4 | Activating_transcription_factor_of_chaperone | -5.1 | - | - | - | - | -6.97 | 5.76 | - | - |
| DN24004_c0_g1_i11 |  | -9.19 | - | 3.63 | - | - | - | 5.95 | - | - |
| DN2415_c5_g1_i12 | Sodium-coupled_monocarboxylate_transporter_1 | 2.94 | - | - | - | -2.65 | - | - | - | 2.33 |
| DN28726_c0_g2_i2 |  | 10.75 | - | - | - | - | - | - | - | - |
| DN715_c1_g1_i29 | SH3_domain-binding_protein_5_homolog | 3.21 | - | - | - | - | - | - | - | 2.38 |
| DN11689_c0_g1_i34 | Acidic_mammalian_chitinase | 3.31 | - | - | - | - | - | -4.33 | - | - |
| DN2673_c0_g1_i15 |  | -3.6 | - | - | - | - | - | - | - | -3.14 |
| DN2415_c5_g1_i7 |  | 3.2 | - | - | - | -2.74 | - | -1.95 | - | 2.57 |
| DN2173_c1_g1_i1 | Aromatic-L-amino-acid_decarboxylase | 12.67 | - | - | - | - | - | - | - | 12.65 |
| DN3023_c1_g1_i2 |  | 8.23 | - | - | - | - | - | - | - | 5.44 |
| DN10228_c0_g1_i8 | Hemocyte_protein-glutamine_gamma-glutamyltransferase | 3.76 | - | -3.5 | - | - | - | - | - | - |
| DN2418_c0_g3_i1 |  | -3.55 | - | 3.52 | 2.72 | 3.01 | - | 3.23 | - | -3.04 |
| DN8397_c0_g2_i5 | C-1-tetrahydrofolate_synthase_cytoplasmic | -8.6 | - | - | - | - | - | - | - | - |
| DN297_c0_g2_i25 | Serine_protease_snake/rpSP30 | -3.14 | - | 2.68 | - | - | - | - | - | -2.53 |
| DN2317_c0_g1_i2 | Mitochondrial_basic_amino_acids_transporter | -6.43 | - | - | - | - | - | - | - | -5.41 |
| DN2423_c0_g1_i27 | Putative_inorganic_phosphate_cotransporter | 5.99 | - | - | - | - | - | - | - | - |
| DN3455_c0_g1_i6 | Protein_neuralized | 2.59 | - | - | - | - | - | - | - | - |
| DN9648_c0_g1_i5 | Sestrin_homolog | -2.7 | - | 2.23 | - | - | - | - | - | - |
| DN13436_c0_g1_i2 | ATP-binding_cassette_sub-family_D_member | -3 | - | 2.51 | - | - | - | - | - | -3.12 |
| DN2415_c5_g1_i8 | Sodium-coupled_monocarboxylate_transporter_1 | 3.61 | - | - | - | -2.67 | - | -2.11 | - | 2.66 |
| DN4957_c0_g1_i6 |  | -11.38 | - | - | - | - | - | - | - | - |
| DN1682_c0_g1_i17 | Phosphatidylcholine:ceramide_cholinephosphotransferase_1 | 7.56 | - | -8.01 | - | - | - | - | - | - |
| DN659_c0_g1_i74 |  | -11.31 | - | - | - | - | - | - | - | - |
| DN674_c1_g1_i1 | Protein_extra-macrochaetae | -2.57 | - | 1.93 | - | - | - | - | - | -2.56 |
| DN3053_c2_g1_i1 | Androgen-induced_gene_1_protein | -4.15 | - | 4.09 | - | - | - | - | - | -3.53 |
| DN847_c1_g1_i16 |  | -2.75 | - | - | - | - | - | - | - | - |
| DN2514_c4_g1_i9 | Gonadotropin-releasing_hormone_receptor | -3.14 | - | 2.83 | - | - | - | - | - | -2.7 |
| DN105_c0_g3_i4 | Pancreatic_triacylglycerol_lipase | 8.86 | - | - | - | - | - | - | - | 7.63 |
| DN6398_c0_g1_i4 |  | -2.43 | - | - | - | - | - | - | - | - |
| DN20238_c0_g1_i5 | E3_ubiquitin-protein_ligase_MYLIP | -2.56 | - | - | - | - | - | - | - | - |
| DN12582_c0_g1_i1 |  | 3.11 | - | - | - | - | - | - | - | - |
| DN2317_c0_g1_i1 |  | -8.85 | - | - | - | - | - | - | - | - |
| DN515_c0_g1_i8 |  | 9.24 | - | - | - | - | - | -9.25 | - | - |
| DN1545_c0_g1_i9 | L-threonine_ammonia-lyase | -7.6 | - | 3.78 | - | - | - | - | - | - |
| DN3262_c0_g1_i1 | Serine_protease_inhibitor_88Ea/ rpSPI10a | 3.59 | - | - | - | - | - | - | - | 3.61 |
| DN3530_c0_g1_i4 |  | 3.11 | - | - | - | - | - | - | - | 3.14 |
| DN161_c4_g1_i1 |  | -2.96 | - | 2.56 | - | - | - | - | - | - |
| DN5394_c0_g1_i14 |  | -3.53 | - | 3.28 | - | - | - | - | - | - |
| DN2173_c1_g1_i3 | Aromatic-L-amino-acid_decarboxylase | 7.9 | - | - | - | - | - | - | - | - |
| DN552_c3_g1_i12 |  | -3.14 | - | 3.93 | - | - | - | - | - | - |
| DN1774_c0_g1_i3 |  | 2.91 | - | - | - | -2.31 | - | - | - | - |
| DN1172_c0_g2_i1 |  | -5.81 | - | - | - | - | - | - | - | -5.15 |
| DN4021_c0_g1_i12 |  | 2.7 | - | - | - | - | - | - | - | 2.35 |
| DN1455_c1_g1_i10 |  | -4.45 | - | - | - | - | - | - | - | -4.26 |
| DN2673_c0_g1_i14 |  | -3.82 | - | - | - | - | - | - | - | -4.12 |
| DN17418_c0_g1_i1 |  | 3.56 | - | - | - | -3.44 | - | - | - | - |
| DN2650_c9_g1_i1 | Zinc_finger_protein_395 | 2.5 | - | - | - | - | - | - | - | - |
| DN17682_c0_g1_i1 |  | 2.71 | - | - | - | - | - | - | - | 2.23 |
| DN5251_c0_g1_i5 |  | 2.81 | - | - | - | - | - | - | - | 2.46 |
| DN3262_c0_g1_i5 | Serine_protease_inhibitor_88Ea/ rpSPI10c | 3.55 | - | - | - | - | - | - | - | 3.43 |
| DN715_c1_g1_i6 | SH3_domain-binding_protein_5_homolog | 2.3 | - | - | - | - | - | - | - | 2.66 |
| DN1455_c1_g1_i19 | Nidogen | -3.36 | - | - | - | - | - | - | - | -2.97 |
| DN595_c0_g1_i20 | Diacylglycerol_O-acyltransferase_2 | -2.56 | - | 2.49 | - | - | - | - | - | -2.79 |
| DN3211_c1_g1_i1 |  | -7.4 | - | - | - | - | - | - | - | - |
| DN76_c0_g1_i12 |  | -3.31 | - | 3.31 | - | - | - | - | - | -2.8 |
| DN13964_c0_g1_i3 |  | -5.51 | - | 7.2 | - | - | - | - | - | -4.99 |
| DN7103_c1_g2_i2 | Apolipophorins | -2.51 | - | 2.35 | - | - | - | - | - | - |
| DN18545_c0_g2_i4 |  | 5.62 | - | - | - | - | - | - | - | 5.63 |
| DN1774_c0_g1_i6 | Beta-1,3-glucan-binding_protein | 2.41 | - | - | - | -2.05 | - | -1.61 | - | - |
| DN11689_c0_g1_i14 |  | 3.62 | - | - | - | - | - | - | - | 4.12 |
| DN1410_c0_g1_i25 |  | -2.87 | - | 3.34 | - | - | - | - | - | - |
| DN51_c0_g1_i3 | Protein_tyrosine_phosphatase_type_IVA_1 | 4.02 | - | - | - | - | -3.69 | - | - | - |
| DN4352_c0_g1_i3 |  | 3.64 | - | - | - | - | - | - | - | - |
| DN297_c0_g2_i22 | rpSP30 | -3.57 | - | - | - | - | - | - | - | - |
| DN4021_c0_g1_i5 |  | 2.72 | - | - | - | -3.02 | - | - | - | 2.9 |
| DN4026_c0_g2_i5 | Glycogen-binding_subunit_76A | -2.08 | - | 2.06 | - | - | - | - | - | -2.07 |
| DN6411_c0_g1_i18 | Bifunctional_purine_biosynthesis_protein_ATIC | -4.59 | - | - | - | - | - | - | - | - |
| DN5058_c0_g1_i2 | SPARC | -5.25 | - | - | - | - | - | - | - | - |
| DN297_c0_g2_i11 | Serine_protease_snake/rpSP30 | -2.97 | - | - | - | - | - | - | - | - |
| DN3024_c0_g1_i1 |  | -4.29 | - | - | - | - | - | - | - | - |
| DN260_c3_g1_i4 | Apolipophorins | -2.12 | - | 2.4 | - | - | - | - | - | -2.2 |
| DN2423_c0_g1_i18 | Putative_inorganic_phosphate_cotransporter | 7.86 | - | - | - | - | -4.42 | - | - | - |
| DN1944_c1_g1_i1 |  | 3.2 | - | - | - | - | - | - | - | - |
| DN7867_c0_g1_i2 | CD63_antigen | -7.32 | - | - | - | - | - | - | - | - |
| DN448_c0_g1_i10 |  | 3.64 | - | - | - | - | - | - | - | - |
| DN610_c3_g1_i17 | Ribose-phosphate_pyrophosphokinase_1 | -3.38 | - | - | - | - | - | - | - | -3.32 |
| DN1024_c0_g1_i5 |  | -3.04 | - | - | - | - | - | - | - | - |
| DN57_c0_g1_i28 | Xanthine_dehydrogenase | -1.78 | - | - | - | - | - | - | - | -1.67 |
| DN297_c0_g2_i21 | rpSP30 | 4.32 | - | - | - | - | - | - | - | - |
| DN30_c1_g1_i11 | RNA_exonuclease_1_homolog | 1.82 | - | - | - | - | - | - | - | - |
| DN1189_c0_g1_i18 |  | -1.51 | - | 1.2 | - | - | - | - | - | -1.42 |
| DN2418_c0_g1_i6 |  | -3.24 | - | - | - | - | - | - | - | - |
| DN4404_c0_g1_i1 | Cadherin_EGF_LAG_seven-pass_G-type_receptor_1 | -4.5 | - | 3.92 | - | - | - | - | - | - |
| DN363_c0_g1_i2 | S-adenosylmethionine_decarboxylase_proenzyme | -1.86 | - | - | - | - | - | - | - | -1.73 |
| DN14975_c0_g1_i9 | Elongation_of_very_long_chain_fatty_acids_protein_AAEL008004 | 2.72 | - | - | - | - | - | - | - | 2.98 |
| DN2658_c0_g1_i16 | Beta-1,3-glucan-binding_protein | 2.51 | - | - | - | -2.62 | - | - | - | 2.39 |
| DN1410_c0_g1_i71 | Hexokinase_type_2 | -2.84 | - | - | - | - | - | - | - | - |
| DN2971_c0_g1_i61 | Putative_phospholipase_B-like_lamina_ancestor | 2.79 | - | - | - | - | - | - | - | - |
| DN638_c5_g1_i10 | Solute_carrier_family_2,_facilitated_glucose_transporter_member_1 | 3.24 | - | - | - | - | - | - | - | - |
| DN14907_c1_g1_i3 |  | 4.41 | - | - | - | -3.38 | - | - | - | - |
| DN68_c0_g1_i29 | Gelsolin | -2.84 | - | 3.62 | - | - | - | - | -2.5 | -2.94 |
| DN6411_c0_g1_i11 | Bifunctional_purine_biosynthesis_protein_ATIC | -3.25 | - | - | - | - | - | - | - | -3.03 |
| DN659_c0_g1_i54 |  | -4.46 | - | - | - | - | - | - | - | -4.63 |
| DN847_c1_g1_i21 |  | -2.44 | - | - | - | - | - | - | - | - |
| DN19492_c0_g2_i1 | Nocturnin | -3.55 | - | 4.33 | - | - | - | - | - | - |
| DN8397_c0_g1_i3 |  | -2.78 | - | - | - | - | - | - | - | - |
| DN2714_c0_g1_i1 | Protein_FAM160B1 | 1.73 | - | - | - | - | - | - | - | 1.81 |
| DN2857_c1_g2_i2 | Timeless | -2.09 | - | - | - | - | - | - | - | -2.17 |
| DN6357_c0_g1_i7 |  | 3.01 | - | - | - | - | - | - | - | - |
| DN1162_c0_g1_i38 |  | -6.91 | - | 8.41 | - | - | - | - | - | - |
| DN76_c0_g2_i2 |  | -2.66 | - | 3.05 | - | - | - | - | - | - |
| DN686_c0_g2_i11 | Ejaculatory_bulb-specific_protein_3 | 2.26 | - | - | - | -2.19 | - | - | - | 1.92 |
| DN2903_c2_g1_i2 | Amidophosphoribosyltransferase | -3.36 | - | - | - | - | - | - | - | -3.03 |
| DN23_c3_g1_i2 |  | -4.58 | - | - | - | - | - | - | - | - |
| DN1401_c0_g2_i4 | G-protein-signaling_modulator_2 | 1.59 | - | - | - | - | - | - | - | - |
| DN1861_c0_g1_i1 | Lysosomal_alpha-mannosidase | -2.73 | - | - | - | - | - | - | - | -2.19 |
| DN2415_c5_g1_i5 |  | 3.51 | - | - | - | - | - | - | - | - |
| DN5731_c0_g1_i31 |  | 3.37 | - | - | - | -11.92 | - | - | - | 10.79 |
| DN610_c3_g1_i18 | Ribose-phosphate_pyrophosphokinase_1 | -3.34 | - | - | - | - | - | - | - | - |
| DN272_c0_g1_i13 | NAD-dependent_protein_deacylase_sirtuin-5,_mitochondrial | -1.73 | - | - | - | - | - | - | - | - |
| DN3483_c0_g1_i26 | Tyrosine hydroxlase (TH) | 2.9 | - | - | - | - | - | - | - | - |
| DN4879_c0_g2_i5 |  | -2.19 | - | 2.26 | - | - | - | - | - | -2.43 |
| DN62_c0_g1_i2 | Calcium-binding_mitochondrial_carrier_protein_SCaMC-2-A | 2.3 | - | - | - | - | - | - | - | - |
| DN191_c0_g2_i5 |  | 4.27 | - | - | - | - | - | - | - | - |
| DN3436_c0_g1_i1 | Cytochrome_P450_6a2 | -3.84 | - | 3.8 | - | - | - | - | - | - |
| DN1455_c1_g1_i17 | Nidogen | -2.27 | - | 3.85 | - | - | - | - | - | - |
| DN3868_c0_g1_i17 | Apoptosis_inhibitor_IAP | 1.56 | - | - | - | - | - | - | - | - |
| DN1861_c0_g1_i8 | Lysosomal_alpha-mannosidase | -2.44 | - | - | - | - | - | - | - | - |
| DN286_c0_g1_i5 | Hexokinase_type_2 | 2.82 | - | - | - | - | - | - | - | - |
| DN158_c0_g1_i15 |  | 1.51 | - | - | - | - | - | - | - | - |
| DN5323_c0_g1_i14 |  | -2.51 | - | - | - | - | - | - | - | - |
| DN7313_c0_g1_i2 | Arylsulfatase_B | 5.75 | - | - | - | - | - | - | - | 4.92 |
| DN328_c0_g1_i8 |  | 1.8 | - | - | - | - | - | - | - | - |
| DN6977_c0_g1_i1 | Protein_60A | 1.61 | - | - | - | - | - | - | - | - |
| DN2106_c0_g1_i22 |  | -2.77 | - | - | - | - | - | - | - | - |
| DN359_c1_g1_i19 |  | 1.54 | - | - | - | - | - | - | - | - |
| DN2489_c0_g1_i9 | Cytochrome_P450_6a2 | -5.37 | - | - | - | - | - | - | - | - |
| DN62_c0_g4_i1 |  | 1.93 | - | - | - | - | - | - | - | - |
| DN6080_c0_g2_i4 | Phenylalanine hydroxylase (PAH) | -2.53 | - | - | - | - | - | - | - | - |
| DN2395_c3_g1_i1 |  | 2.02 | - | - | - | - | - | - | - | - |
| DN260_c3_g1_i9 | Apolipophorins | -2.23 | - | - | - | - | - | - | - | -2.12 |
| DN3455_c0_g1_i1 | Protein_neuralized | 2.47 | - | - | - | - | - | - | - | - |
| DN6273_c0_g1_i8 |  | -2.65 | - | 2.35 | - | - | - | - | - | - |
| DN354_c0_g1_i1 |  | -2.82 | - | 2.69 | - | - | - | - | - | - |
| DN2626_c0_g1_i2 |  | - | 12.17 | - | - | - | - | - | - | - |
| DN301_c0_g2_i2 | ER_membrane_protein_complex_subunit_7_homolog | - | -11.63 | - | - | - | - | - | - | - |
| DN3418_c4_g1_i4 |  | - | -11.28 | - | - | - | - | - | - | - |
| DN2603_c0_g1_i12 |  | - | 11.19 | - | - | - | - | - | - | - |
| DN671_c0_g1_i10 | Echinoderm_microtubule-associated_protein-like_1 | - | 11.32 | - | - | - | - | - | - | - |
| DN6212_c1_g1_i5 |  | - | 10.73 | 11.65 | - | - | - | - | -11.69 | - |
| DN7100_c0_g1_i5 |  | - | -10.63 | - | - | - | - | - | - | - |
| DN4450_c0_g1_i21 |  | - | 8 | - | - | - | - | - | - | - |
| DN9420_c0_g2_i5 | Probable_aminopeptidase_NPEPL1 | - | -2.37 | - | - | - | - | - | - | - |
| DN1385_c4_g1_i6 |  | - | 2.68 | - | - | - | - | - | - | - |
| DN8665_c0_g1_i1 | Protein_pinocchio | - | - | -13.17 | - | - | - | - | - | - |
| DN1204_c0_g1_i40 |  | - | - | 11.65 | - | - | - | - | - | - |
| DN1423_c0_g1_i44 | Desumoylating_isopeptidase_1 | - | - | -8.51 | - | - | - | - | - | - |
| DN2817_c0_g1_i10 |  | - | - | 11.16 | - | - | - | - | - | - |
| DN2106_c0_g1_i17 |  | - | - | 11.26 | - | - | - | - | - | - |
| DN1907_c0_g2_i9 |  | - | - | -11.21 | - | 10.84 | - | - | 11.27 | - |
| DN49_c9_g1_i10 | J_domain-containing_protein | - | - | -11.58 | - | - | -14.26 | - | - | - |
| DN5498_c0_g1_i4 |  | - | - | -10.93 | - | - | - | - | - | - |
| DN138_c1_g1_i1 | Protein_NATD1 | - | - | 2.53 | - | - | - | - | - | - |
| DN324_c1_g1_i7 | DnaJ_homolog_subfamily_C_member_7 | - | - | -9.02 | - | - | - | - | - | - |
| DN503_c2_g1_i2 | Defensin | - | - | 2.1 | - | - | - | - | - | - |
| DN2423_c0_g1_i1 | Putative_inorganic_phosphate_cotransporter | - | - | -12.2 | - | - | - | - | - | - |
| DN966_c0_g1_i4 | 5'-AMP-activated_protein_kinase_subunit_gamma-1 | - | - | 2.76 | - | - | - | 2.28 | - | - |
| DN3608_c0_g1_i12 | Putative_inorganic_phosphate_cotransporter | - | - | 10.47 | - | - | - | - | - | - |
| DN613_c1_g1_i4 |  | - | - | -10.8 | - | - | - | - | 10.86 | - |
| DN978_c0_g1_i14 | Facilitated_trehalose_transporter_Tret1 | - | - | 2.71 | - | - | - | 2.1 | -2.03 | - |
| DN13756_c0_g1_i3 |  | - | - | -10.35 | - | - | - | - | - | - |
| DN633_c0_g1_i5 |  | - | - | -10.47 | - | - | - | - | - | - |
| DN1788_c0_g1_i1 |  | - | - | -11.13 | - | - | - | - | 11.21 | - |
| DN15434_c0_g2_i1 |  | - | - | 2.97 | - | - | - | - | - | - |
| DN6295_c1_g1_i1 |  | - | - | 3.17 | 3.08 | - | - | 2.94 | - | - |
| DN68_c0_g1_i2 | Gelsolin | - | - | 2.65 | - | - | - | - | - | -2.18 |
| DN68_c0_g1_i12 | Gelsolin | - | - | 2.63 | - | - | - | - | - | - |
| DN13772_c0_g1_i8 | Delta-1-pyrroline-5-carboxylate_synthase | - | - | 2.7 | - | - | - | - | - | - |
| DN15545_c0_g1_i1 |  | - | - | 2.48 | 2.49 | - | - | 2.2 | - | - |
| DN27360_c0_g1_i4 | Defensin | - | - | 2.29 | - | - | - | - | - | - |
| DN76_c0_g1_i11 |  | - | - | 2.55 | - | - | - | - | - | - |
| DN76_c0_g2_i1 |  | - | - | 1.83 | - | - | - | - | - | - |
| DN16_c3_g1_i16 | Chitinase-like_protein_EN03 | - | - | 2.86 | - | - | - | - | - | - |
| DN13324_c0_g1_i2 |  | - | - | 3.5 | 3.08 | - | - | 3.33 | - | - |
| DN16_c3_g1_i6 | Chitinase-like_protein_EN03 | - | - | 2.72 | - | - | - | - | - | - |
| DN1470_c2_g1_i1 | Protein_eiger | - | - | -4.14 | - | - | - | - | - | - |
| DN4428_c1_g1_i1 |  | - | - | -10.34 | - | - | - | - | - | - |
| DN110_c0_g1_i3 |  | - | - | 7.37 | - | - | - | - | - | - |
| DN2263_c0_g1_i13 | Defensin | - | - | 2.27 | - | - | - | - | - | - |
| DN1791_c0_g1_i10 |  | - | - | -2.86 | - | - | - | -3.01 | - | - |
| DN22347_c0_g1_i5 | Unconventional_myosin_IC | - | - | 1.56 | - | - | - | - | - | - |
| DN298_c1_g1_i4 | Transcription_factor_HNF-4_homolog | - | - | 2.56 | - | - | - | - | - | - |
| DN4715_c0_g1_i7 |  | - | - | -7.01 | - | - | - | - | - | - |
| DN2418_c0_g1_i12 | Mariner_Mos1_transposase | - | - | 2.07 | - | - | - | - | - | - |
| DN3934_c1_g2_i1 | General_odorant-binding_protein_56d | - | - | 2.4 | - | - | - | - | - | - |
| DN1178_c0_g1_i17 |  | - | - | 4.92 | - | - | - | - | - | - |
| DN3340_c0_g1_i6 |  | - | - | -10.16 | - | - | - | - | - | - |
| DN2903_c0_g1_i5 |  | - | - | 1.59 | - | - | - | - | - | - |
| DN3950_c0_g1_i29 |  | - | - | -2.67 | - | - | - | - | 5.01 | - |
| DN2263_c0_g1_i9 | Defensin | - | - | 2.23 | - | - | - | - | - | - |
| DN683_c2_g1_i5 | Lipoyltransferase_1,_mitochondrial | - | - | 2.87 | - | - | - | - | - | - |
| DN2187_c0_g2_i1 | Transferrin | - | - | 2.32 | - | - | - | - | - | - |
| DN2726_c0_g1_i44 |  | - | - | 1.39 | - | - | - | - | - | - |
| DN4864_c0_g1_i7 | Inositol_1,4,5-triphosphate_receptor_associated_1 | - | - | 2.26 | - | - | - | 1.74 | - | - |
| DN140_c0_g1_i12 |  | - | - | 1.6 | - | - | - | - | - | - |
| DN6949_c0_g2_i2 | cAMP-specific_3',5'-cyclic_phosphodiesterase | - | - | 1.64 | - | - | - | - | - | - |
| DN4733_c0_g2_i3 |  | - | - | 3.01 | - | - | - | - | - | - |
| DN6409_c0_g2_i9 | DCN1-like_protein_4 | - | - | -2.2 | - | - | - | - | - | - |
| DN10717_c0_g1_i8 | Anoctamin-1 | - | - | -2.38 | - | - | - | - | - | - |
| DN318_c0_g1_i32 |  | - | - | 1.58 | - | - | - | - | - | - |
| DN671_c0_g1_i1 | Echinoderm_microtubule-associated_protein-like_1 | - | - | 2.4 | - | - | - | - | - | - |
| DN2627_c0_g1_i4 | Aquaporin-2 | - | - | -1.3 | - | - | - | - | - | - |
| DN3948_c0_g1_i5 | Heat_shock_70_kDa_protein_cognate_4 | - | - | -2.09 | - | - | - | - | - | - |
| DN653_c1_g1_i10 | U1_small_nuclear_ribonucleoprotein_70_kDa | - | - | 1.42 | - | - | - | - | - | - |
| DN20_c3_g1_i2 | Defensin | - | - | 1.72 | - | - | - | - | - | - |
| DN2199_c0_g1_i5 |  | - | - | -1.77 | - | - | - | - | - | - |
| DN552_c3_g1_i11 |  | - | - | 2.71 | - | - | - | - | - | - |
| DN8312_c0_g1_i2 | Diacylglycerol_O-acyltransferase_1 | - | - | 1.55 | - | - | - | - | - | - |
| DN5411_c0_g1_i5 |  | - | - | -1.81 | - | - | - | - | - | - |
| DN3934_c0_g1_i3 |  | - | - | 2.98 | - | - | - | - | - | - |
| DN9084_c0_g3_i1 |  | - | - | 1.47 | - | - | - | - | - | - |
| DN106_c1_g1_i6 | Neurotrypsin/rpSP2 | - | - | 1.43 | - | - | - | - | - | - |
| DN147_c0_g1_i13 | Ninein-like_protein | - | - | 5.53 | - | - | - | - | -5.94 | - |
| DN1404_c3_g1_i1 |  | - | - | 1.92 | - | - | - | - | - | - |
| DN10341_c0_g1_i10 |  | - | - | 2.1 | - | - | - | - | - | - |
| DN20326_c0_g1_i1 |  | - | - | 3.1 | - | - | - | - | - | - |
| DN725_c2_g1_i11 |  | - | - | -1.98 | - | - | - | - | - | - |
| DN3887_c0_g1_i5 | Microsomal_triglyceride_transfer_protein_large_subunit | - | - | 2.81 | - | - | - | - | - | - |
| DN3625_c0_g1_i2 |  | - | - | 1.94 | - | - | - | - | - | - |
| DN1989_c0_g1_i25 | Mast_cell_protease_1/rpSP21 | - | - | -1.55 | - | - | - | - | - | - |
| DN10669_c0_g1_i2 | Zinc_finger_protein_Noc | - | - | 1.65 | - | - | - | - | - | - |
| DN248_c0_g2_i1 | Facilitated_trehalose_transporter_Tret1 | - | - | 2.78 | - | - | - | - | - | - |
| DN4549_c0_g1_i8 | Beta-galactosidase | - | - | -1.67 | - | - | - | - | - | - |
| DN2293_c0_g1_i2 | 2-acylglycerol_O-acyltransferase_2 | - | - | 1.9 | - | - | - | - | - | - |
| DN15295_c0_g1_i2 | Probable_aquaporin_PIP2-8 | - | - | -1.22 | - | - | - | - | - | - |
| DN459_c0_g1_i1 |  | - | - | 1.58 | - | - | - | - | - | - |
| DN19798_c0_g2_i4 | Luciferin_4-monooxygenase | - | - | 2.79 | - | - | - | - | - | - |
| DN1362_c0_g1_i6 | Constitutive_coactivator_of_PPAR-gamma-like_protein_1 | - | - | 1.54 | - | - | - | - | - | - |
| DN2978_c0_g1_i2 |  | - | - | 1.8 | - | - | - | - | - | - |
| DN7569_c0_g1_i1 |  | - | - | 1.86 | - | - | - | - | - | - |
| DN257_c0_g1_i19 | Eukaryotic_translation_initiation_factor_4_gamma_2 | - | - | -1.85 | - | - | - | - | - | - |
| DN2867_c0_g2_i1 | Rab_GTPase-binding_effector_protein_1 | - | - | 1.42 | - | - | - | - | - | - |
| DN4440_c0_g1_i1 |  | - | - | 1.56 | - | - | - | - | - | - |
| DN1421_c0_g1_i26 | Serine/threonine-protein_kinase_PRP4_homolog | - | - | 1.32 | - | - | - | - | - | - |
| DN298_c1_g1_i2 | Transcription_factor_HNF-4_homolog | - | - | 1.96 | - | - | - | - | - | - |
| DN18434_c0_g1_i1 |  | - | - | 1.68 | - | - | - | - | - | - |
| DN1664_c0_g1_i7 | Branched-chain-amino-acid_aminotransferase | - | - | 1.62 | - | - | - | - | - | - |
| DN3181_c0_g1_i3 | Luciferin_sulfotransferase | - | - | 3.01 | - | - | - | - | - | - |
| DN683_c2_g1_i3 |  | - | - | 2.37 | - | - | - | 1.9 | - | - |
| DN60_c2_g2_i5 |  | - | - | 4.2 | - | - | - | - | - | - |
| DN4476_c0_g1_i1 |  | - | - | 1.8 | - | - | - | - | - | - |
| DN3857_c0_g1_i2 | RNA-binding_protein_fusilli | - | - | 2.35 | - | - | - | - | - | - |
| DN2045_c0_g1_i31 |  | - | - | -7.35 | - | - | - | - | - | - |
| DN6626_c0_g2_i2 | Pyrroline-5-carboxylate_reductase_3 | - | - | 2.18 | - | - | - | - | - | - |
| DN1781_c0_g1_i3 |  | - | - | -1.54 | - | - | - | - | - | - |
| DN995_c1_g1_i9 | Four_and_a_half_LIM_domains_protein_2_ | - | - | 1.73 | - | - | - | - | - | - |
| DN12922_c0_g1_i9 |  | - | - | 2.24 | - | - | - | - | - | - |
| DN11960_c0_g1_i7 | Lysosomal_aspartic_protease | - | - | 1.97 | - | - | - | - | - | - |
| DN847_c4_g1_i2 |  | - | - | 2.29 | - | - | - | - | - | - |
| DN23_c2_g1_i8 |  | - | - | 1.5 | - | - | - | - | - | - |
| DN4052_c0_g2_i1 | Vitellogenin-6 | - | - | 1.67 | - | - | - | - | - | - |
| DN4168_c0_g1_i3 |  | - | - | 6.04 | - | - | - | - | - | - |
| DN4508_c0_g1_i21 |  | - | - | 1.33 | - | - | - | - | - | - |
| DN1204_c1_g2_i2 | A-kinase_anchor_protein_1,_mitochondrial | - | - | -2.14 | - | - | - | - | - | - |
| DN630_c0_g1_i7 |  | - | - | 2.02 | - | - | - | - | - | - |
| DN13772_c0_g1_i4 | Delta-1-pyrroline-5-carboxylate_synthase | - | - | 2.11 | - | - | - | - | - | - |
| DN14838_c0_g1_i1 | Lipase_3 | - | - | 1.69 | - | - | - | - | - | - |
| DN630_c0_g1_i1 |  | - | - | 2.64 | - | - | - | - | - | - |
| DN17596_c0_g1_i7 |  | - | - | 1.56 | - | - | - | - | - | - |
| DN651_c0_g1_i2 | Glutamyl_aminopeptidase | - | - | 1.23 | - | - | - | - | - | - |
| DN1270_c0_g1_i1 |  | - | - | 1.78 | - | - | - | - | - | - |
| DN1655_c3_g1_i1 | Alpha-(1,6)-fucosyltransferase | - | - | 1.2 | - | - | - | - | - | - |
| DN318_c0_g1_i24 |  | - | - | 1.65 | - | - | - | - | - | - |
| DN1401_c0_g2_i3 | G-protein-signaling_modulator_2 | - | - | 1.42 | - | - | - | - | - | - |
| DN5875_c0_g1_i1 |  | - | - | 1.49 | - | - | - | - | - | - |
| DN12690_c0_g1_i1 |  | - | - | -1.17 | - | - | - | - | - | - |
| DN1120_c0_g1_i2 |  | - | - | 1.67 | - | - | - | - | - | - |
| DN9476_c0_g1_i1 |  | - | - | -1.39 | - | - | - | - | - | - |
| DN148_c14_g1_i1 | Teneurin-m | - | - | 1.38 | - | - | - | - | - | - |
| DN28504_c0_g1_i3 |  | - | - | 1.63 | - | - | - | - | - | - |
| DN28504_c0_g1_i5 |  | - | - | 1.7 | - | - | - | - | - | - |
| DN6125_c0_g1_i6 |  | - | - | -1.46 | - | - | - | - | - | - |
| DN606_c1_g1_i18 |  | - | - | -1.13 | - | - | - | - | - | - |
| DN2537_c0_g1_i14 |  | - | - | 3.12 | - | - | - | - | - | - |
| DN261_c0_g1_i9 | AMP_deaminase_2 | - | - | 2.07 | - | - | - | - | - | - |
| DN2603_c0_g1_i20 |  | - | - | -2.44 | - | - | - | - | - | - |
| DN2539_c0_g1_i2 | Protein_NipSnap | - | - | 1.99 | - | - | - | - | - | - |
| DN3857_c0_g1_i6 |  | - | - | 2.25 | - | - | - | - | - | - |
| DN3819_c0_g1_i6 | Upstream-binding_protein_1 | - | - | 1.75 | - | - | - | - | - | - |
| DN494_c0_g1_i28 |  | - | - | -2.75 | - | - | - | -3.06 | - | - |
| DN3724_c0_g1_i4 | Spectrin_beta_chain,_non-erythrocytic_5 | - | - | 1.4 | - | - | - | - | - | - |
| DN441_c0_g2_i11 |  | - | - | 1.88 | - | - | - | - | - | - |
| DN2673_c0_g1_i11 |  | - | - | 2.36 | - | - | - | - | - | - |
| DN1838_c0_g1_i1 |  | - | - | 2.41 | - | - | - | - | - | - |
| DN550_c0_g1_i3 |  | - | - | -2.65 | - | - | - | - | - | - |
| DN18985_c0_g1_i4 |  | - | - | -2.83 | - | - | - | - | - | - |
| DN2418_c0_g1_i5 | Mariner_Mos1_transposase | - | - | 1.78 | - | - | - | - | - | - |
| DN19798_c0_g2_i2 | Luciferin_4-monooxygenase | - | - | 2.71 | - | - | - | - | - | - |
| DN459_c0_g2_i3 |  | - | - | -1.8 | - | - | - | -2.08 | - | - |
| DN461_c2_g1_i4 |  | - | - | 1.4 | - | - | - | - | - | - |
| DN726_c0_g1_i27 | UDP-glucose_4-epimerase | - | - | -1.63 | - | - | - | - | - | - |
| DN421_c0_g1_i4 |  | - | - | -1.37 | - | - | - | - | - | - |
| DN2576_c0_g1_i1 |  | - | - | -1.65 | - | - | - | - | - | - |
| DN4394_c0_g1_i36 |  | - | - | 1.91 | - | - | - | - | - | - |
| DN3558_c0_g2_i5 | Protein_phosphatase_1B | - | - | 1.18 | - | - | - | - | - | - |
| DN354_c2_g2_i3 | Dipeptidyl_peptidase_9 | - | - | -1.92 | - | - | - | - | 12.15 | - |
| DN2024_c1_g1_i25 | Calpain-A | - | - | -1.23 | - | - | - | - | - | - |
| DN611_c0_g1_i2 | Dihydropyrimidinase | - | - | -1.12 | - | - | - | - | - | - |
| DN1157_c0_g1_i3 | Aldo-keto_reductase_family_1_member_A1 | - | - | -1.26 | - | - | - | - | - | - |
| DN1078_c0_g1_i4 |  | - | - | 2.1 | - | - | - | - | - | - |
| DN5604_c0_g1_i7 |  | - | - | 1.43 | - | - | - | - | - | - |
| DN7297_c0_g1_i4 | AP-2_complex_subunit_alpha | - | - | 1.34 | - | - | - | - | - | - |
| DN1380_c0_g1_i1 |  | - | - | 1.88 | - | - | - | - | - | - |
| DN12514_c0_g1_i2 | Farnesyl_pyrophosphate_synthase | - | - | 1.25 | - | - | - | - | - | - |
| DN4045_c0_g1_i23 | Titin | - | - | 1.77 | - | - | - | - | - | - |
| DN8007_c0_g1_i7 |  | - | - | -5.01 | - | - | - | - | - | - |
| DN255_c0_g1_i17 |  | - | - | -2.32 | - | - | - | - | - | - |
| DN3271_c0_g1_i6 | Cytochrome_P450_6j1 | - | - | 1.81 | - | - | - | - | - | - |
| DN2610_c1_g1_i17 | Glycerol_kinase | - | - | 1.15 | - | - | - | - | - | - |
| DN2551_c0_g1_i10 | Ornithine_aminotransferase,_mitochondrial | - | - | -1.14 | - | - | - | - | - | - |
| DN4572_c0_g2_i4 |  | - | - | 1.56 | - | - | - | - | - | - |
| DN1263_c0_g1_i9 |  | - | - | -1.63 | - | - | - | - | - | - |
| DN2216_c0_g1_i23 |  | - | - | 1.51 | - | - | - | - | -2.13 | - |
| DN1091_c0_g1_i5 |  | - | - | 1.56 | - | - | - | - | - | - |
| DN5676_c0_g2_i1 |  | - | - | 1.38 | - | - | - | - | - | - |
| DN421_c0_g1_i2 |  | - | - | -1.42 | - | - | - | - | - | - |
| DN7180_c0_g1_i1 | Prostate-specific_antigen/rpSP59 | - | - | -1.21 | - | - | - | - | - | - |
| DN2673_c0_g1_i16 |  | - | - | 2.2 | - | - | - | - | - | - |
| DN10840_c0_g1_i20 |  | - | - | 1.83 | - | - | - | - | - | - |
| DN5130_c0_g3_i4 | Electron_transfer_flavoprotein-ubiquinone_oxidoreductase,_mitochondrial | - | - | 1.53 | - | - | - | - | - | - |
| DN9990_c0_g1_i2 | Wolframin | - | - | -1.22 | - | - | - | - | - | - |
| DN33611_c0_g1_i1 | rpSP36 | - | - | -1.41 | - | - | - | - | - | - |
| DN3108_c0_g2_i8 | Protein_artichoke | - | - | 1.49 | - | - | - | - | - | - |
| DN2189_c0_g2_i3 | Tetraspanin-9 | - | - | 2.12 | - | - | - | - | - | - |
| DN570_c4_g1_i2 |  | - | - | 1.45 | - | - | - | - | - | - |
| DN3991_c0_g2_i1 |  | - | - | 2.02 | - | - | - | - | - | - |
| DN7103_c1_g2_i1 | Apolipophorins | - | - | 2.07 | - | - | - | - | - | - |
| DN57_c0_g1_i11 |  | - | - | -2.86 | - | - | - | - | - | - |
| DN718_c0_g1_i11 |  | - | - | 1.39 | - | - | - | - | - | - |
| DN16_c3_g1_i14 | Chitinase-like_protein_EN03 | - | - | 2.67 | - | - | - | - | - | - |
| DN4580_c0_g1_i2 |  | - | - | 2.11 | - | - | - | - | - | - |
| DN81_c0_g1_i7 | Cathepsin_L | - | - | 2.25 | - | - | - | - | - | - |
| DN2059_c0_g1_i3 |  | - | - | 3.65 | - | - | - | - | - | - |
| DN878_c0_g2_i3 |  | - | - | -1.32 | - | - | - | - | - | - |
| DN168_c0_g1_i25 | Nitrophorin-2 | - | - | 2.13 | - | - | - | - | - | - |
| DN548_c0_g1_i10 | Protein_O-mannosyl-transferase_2 | - | - | 1.49 | - | - | - | - | - | - |
| DN20_c4_g1_i2 | BTB/POZ_domain-containing_protein_KCTD3 | - | - | 1.31 | - | - | - | - | - | - |
| DN9937_c0_g1_i1 |  | - | - | -1.48 | - | - | - | - | - | - |
| DN925_c3_g1_i1 | Protein-serine_O-palmitoleoyltransferase_porcupine | - | - | -1.42 | - | - | - | - | - | - |
| DN1392_c0_g1_i40 | Nicotinate_phosphoribosyltransferase | - | - | 1.89 | - | - | - | - | - | - |
| DN8511_c0_g3_i2 | Transient_receptor_potential_cation_channel_protein_painless | - | - | 1.35 | - | - | - | - | - | - |
| DN8879_c2_g1_i3 |  | - | - | -4.63 | - | - | - | - | - | - |
| DN255_c0_g1_i7 |  | - | - | -1.3 | - | - | - | - | - | - |
| DN3106_c1_g2_i2 |  | - | - | 1.65 | - | - | - | - | - | - |
| DN148_c2_g1_i5 | Histone-lysine_N-methyltransferase_SETMAR | - | - | 1.8 | - | - | - | - | - | - |
| DN166_c0_g2_i1 |  | - | - | 1.31 | - | - | - | - | - | - |
| DN287_c0_g1_i21 |  | - | - | 4.3 | - | - | - | - | - | - |
| DN283_c0_g1_i7 | Nuclear_factor_related_to_kappa-B-binding_protein | - | - | -1.15 | - | - | - | - | - | - |
| DN8558_c0_g1_i1 | Transmembrane_emp24_domain-containing_protein_bai | - | - | 1.18 | - | - | - | - | - | - |
| DN356_c1_g1_i10 |  | - | - | 2.5 | - | - | - | - | - | - |
| DN514_c0_g1_i3 | Protein_ECT2 | - | - | 1.47 | - | - | - | - | - | - |
| DN905_c11_g1_i1 |  | - | - | 1.12 | - | - | - | - | - | - |
| DN1015_c0_g1_i8 |  | - | - | 1.31 | - | - | - | - | - | - |
| DN17242_c0_g1_i16 |  | - | - | 1.65 | - | - | - | - | - | - |
| DN14098_c0_g1_i1 | Hepatocyte_nuclear_factor_4-alpha | - | - | 1.73 | - | - | - | - | - | - |
| DN11132_c1_g1_i4 | Amino_acid_transporter_AVT1A | - | - | 1.87 | - | - | - | - | - | -2 |
| DN33430_c0_g1_i1 |  | - | - | 1.35 | - | - | - | - | - | - |
| DN1372_c3_g1_i19 | Major_facilitator_superfamily_domain-containing_protein_12 | - | - | 1.4 | - | - | - | - | - | - |
| DN162_c0_g1_i4 | Cold_shock_domain-containing_protein_E1 | - | - | 1.35 | - | - | - | - | - | - |
| DN2263_c0_g1_i16 |  | - | - | 1.78 | - | - | - | - | - | - |
| DN3065_c0_g1_i1 | U-scoloptoxin(11)-Sa2a | - | - | 1.68 | - | - | - | - | - | - |
| DN106_c1_g1_i2 | Neurotrypsin/rpSP2 | - | - | 1.57 | - | - | - | - | - | - |
| DN2490_c0_g1_i4 | P3_protein | - | - | 1.27 | - | - | - | - | - | - |
| DN1569_c2_g1_i2 |  | - | - | 1.54 | - | - | - | - | - | - |
| DN70_c0_g1_i2 |  | - | - | 1.45 | - | - | - | - | - | - |
| DN348_c0_g2_i5 | Epoxide_hydrolase_4 | - | - | 1.69 | - | - | - | - | - | - |
| DN147_c0_g1_i4 |  | - | - | 1.67 | - | - | - | - | - | - |
| DN169_c7_g1_i1 |  | - | - | 3.51 | - | - | - | - | - | - |
| DN7630_c0_g1_i1 |  | - | - | -1.45 | - | - | - | - | - | - |
| DN15949_c0_g2_i8 | Vascular_endothelial_growth_factor_receptor_1 | - | - | 1.14 | - | - | - | - | - | - |
| DN2187_c0_g2_i4 | Transferrin | - | - | 1.41 | - | - | - | - | - | - |
| DN2776_c0_g1_i1 |  | - | - | 1.11 | - | - | - | - | - | - |
| DN5769_c0_g2_i8 |  | - | - | 1.8 | - | - | - | - | - | - |
| DN2268_c0_g1_i1 |  | - | - | 1.67 | - | - | - | - | - | - |
| DN2512_c0_g1_i6 | Cytosolic_carboxypeptidase_Nna1 | - | - | -1.97 | - | - | - | - | - | - |
| DN474_c1_g1_i2 | Gamma-glutamyl_hydrolase | - | - | 1.7 | - | - | - | - | - | - |
| DN222_c1_g2_i1 | Translocon-associated_protein_subunit_beta | - | - | - | 13.04 | - | - | - | - | - |
| DN1082_c4_g1_i30 |  | - | - | - | 11.53 | - | - | - | - | -11.6 |
| DN237_c1_g1_i3 |  | - | - | - | 11.25 | - | - | - | - | -2.39 |
| DN1747_c0_g1_i2 |  | - | - | - | -11.11 | - | - | - | - | - |
| DN4394_c0_g1_i22 |  | - | - | - | 11.22 | - | - | - | - | - |
| DN2121_c0_g1_i5 |  | - | - | - | -11.02 | - | - | - | - | - |
| DN443_c7_g1_i2 |  | - | - | - | -2.99 | -2.6 | - | - | - | 2.7 |
| DN1215_c1_g1_i5 |  | - | - | - | 10.89 | - | - | - | - | - |
| DN6173_c0_g1_i4 |  | - | - | - | 10.98 | - | - | 10.43 | - | - |
| DN404_c0_g1_i1 | Sorting_and_assembly_machinery_component_50_homolog_B_ | - | - | - | -2.66 | - | - | - | - | 2.24 |
| DN403_c8_g1_i6 |  | - | - | - | 10.65 | - | - | - | - | - |
| DN1726_c0_g1_i6 |  | - | - | - | 3.08 | - | - | 3.69 | - | - |
| DN1755_c1_g1_i3 | Bifunctional_glutamate/proline--tRNA_ligase | - | - | - | 2.09 | - | - | - | - | - |
| DN482_c6_g1_i1 |  | - | - | - | -10.66 | - | - | - | - | - |
| DN404_c0_g1_i4 | Sorting_and_assembly_machinery_component_50_homolog_B_ | - | - | - | -2.07 | - | - | - | - | 1.89 |
| DN5394_c0_g1_i35 |  | - | - | - | 5.46 | - | - | - | - | -10.7 |
| DN50_c0_g1_i8 |  | - | - | - | -10.32 | - | - | - | - | - |
| DN2330_c0_g1_i24 |  | - | - | - | 2.77 | - | - | - | - | - |
| DN278_c11_g1_i1 | Protein_TIS11 | - | - | - | 1.49 | - | - | - | - | - |
| DN2412_c0_g1_i15 |  | - | - | - | 3.99 | - | - | - | - | - |
| DN1497_c1_g1_i1 | Ras-related_protein_Rap-2c | - | - | - | 1.94 | - | - | - | - | - |
| DN843_c0_g1_i23 |  | - | - | - | -11.11 | - | - | - | - | - |
| DN7808_c0_g1_i16 |  | - | - | - | 1.84 | - | - | - | - | - |
| DN222_c1_g2_i2 | Translocon-associated_protein_subunit_beta | - | - | - | -1.53 | - | - | - | - | - |
| DN229_c5_g1_i1 |  | - | - | - | -4.24 | - | - | - | - | - |
| DN5303_c0_g2_i6 |  | - | - | - | -1.41 | - | - | - | - | - |
| DN11694_c0_g1_i5 | Transmembrane_7_superfamily_member_3 | - | - | - | - | -13.17 | - | - | - | 13.2 |
| DN638_c3_g1_i5 | Protein_ERGIC-53 | - | - | - | - | -11.02 | - | - | - | 8.65 |
| DN7155_c0_g1_i26 | 4-hydroxybutyrate_coenzyme_A_transferase | - | - | - | - | 12.18 | 11.99 | - | 12.95 | - |
| DN7454_c0_g1_i17 | Protein_phosphatase_1_regulatory_subunit_15A | - | - | - | - | 12.42 | - | - | - | - |
| DN7155_c0_g1_i20 | 4-hydroxybutyrate_coenzyme_A_transferase | - | - | - | - | -12.64 | - | - | - | 10.98 |
| DN1924_c0_g1_i3 |  | - | - | - | - | -9.81 | - | - | - | - |
| DN1372_c7_g1_i5 | Protein_boule | - | - | - | - | 13.23 | - | - | 13.77 | - |
| DN510_c0_g1_i2 | E3_ubiquitin-protein_ligase_MIB2 | - | - | - | - | -12 | - | - | - | 11.87 |
| DN258_c0_g1_i16 | UDP-N-acetylglucosamine--peptide_N-acetylglucosaminyltransferase_110_kDa_subunit | - | - | - | - | 11.3 | 11.77 | - | - | - |
| DN22950_c0_g1_i1 |  | - | - | - | - | 10.89 | - | - | - | - |
| DN1924_c0_g1_i5 |  | - | - | - | - | 11 | - | - | - | - |
| DN5498_c0_g1_i3 |  | - | - | - | - | 10.77 | - | - | - | - |
| DN33_c3_g1_i2 |  | - | - | - | - | -4.39 | - | - | - | - |
| DN1704_c0_g1_i1 |  | - | - | - | - | -9.03 | - | - | - | - |
| DN1215_c1_g1_i15 |  | - | - | - | - | -11.3 | - | - | - | - |
| DN683_c0_g1_i28 | Protein_split_ends | - | - | - | - | 11.04 | - | - | 11.35 | - |
| DN444_c3_g1_i2 |  | - | - | - | - | 10.46 | - | - | - | -10.66 |
| DN320_c0_g1_i1 | Facilitated_trehalose_transporter_Tret1 | - | - | - | - | -2.69 | - | - | - | - |
| DN812_c0_g1_i22 |  | - | - | - | - | 9.15 | 9.24 | - | 9.35 | - |
| DN3300_c0_g1_i30 |  | - | - | - | - | -2.49 | - | - | - | - |
| DN639_c0_g2_i13 |  | - | - | - | - | 10.74 | - | - | - | - |
| DN856_c0_g1_i21 |  | - | - | - | - | 9.13 | - | - | 9.38 | - |
| DN630_c0_g1_i3 |  | - | - | - | - | -2.72 | - | - | - | - |
| DN856_c0_g1_i13 |  | - | - | - | - | 11.06 | - | - | 11.77 | - |
| DN6297_c0_g1_i2 |  | - | - | - | - | -3.23 | - | - | - | - |
| DN2050_c0_g1_i11 | Sialin | - | - | - | - | -2.76 | - | - | - | - |
| DN2050_c0_g1_i14 | Sialin | - | - | - | - | -2.42 | - | - | -2.47 | - |
| DN3619_c0_g1_i2 | ATP_synthase_lipid-binding_protein,_mitochondrial | - | - | - | - | -2.09 | - | - | - | - |
| DN2673_c0_g1_i5 | Protein_lifeguard_1 | - | - | - | - | 3.49 | - | - | 3.69 | - |
| DN596_c0_g1_i6 | Protein_tumorous_imaginal_discs,_mitochondrial | - | - | - | - | -13.04 | - | - | - | - |
| DN2848_c4_g1_i1 |  | - | - | - | - | -2.19 | - | - | - | 2.49 |
| DN2423_c0_g1_i9 | Putative_inorganic_phosphate_cotransporter | - | - | - | - | -5.26 | -3.16 | - | - | 4.91 |
| DN120_c0_g1_i2 | Src_substrate_cortactin | - | - | - | - | 6.31 | - | - | 7.08 | - |
| DN323_c0_g1_i30 | Bifunctional_3'-phosphoadenosine_5'-phosphosulfate_synthase | - | - | - | - | -1.95 | - | - | - | - |
| DN596_c0_g1_i1 | Protein_tumorous_imaginal_discs,_mitochondrial | - | - | - | - | -2.36 | - | - | - | 1.87 |
| DN298_c4_g1_i1 |  | - | - | - | - | -2.22 | - | - | - | - |
| DN382_c0_g1_i1 | cGMP-dependent_protein_kinase,_isozyme_2_forms_cD5/T2 | - | - | - | - | -2.34 | - | - | - | - |
| DN323_c0_g1_i42 | Bifunctional_3'-phosphoadenosine_5'-phosphosulfate_synthase_2 | - | - | - | - | -1.93 | - | - | - | - |
| DN725_c0_g1_i3 |  | - | - | - | - | -2.03 | - | - | - | 2.36 |
| DN6490_c1_g1_i1 |  | - | - | - | - | -7.39 | - | - | -7.28 | - |
| DN2304_c1_g1_i48 | Sensory_neuron_membrane_protein_2 | - | - | - | - | -2.14 | - | - | - | - |
| DN3070_c0_g3_i5 | Vanin-like_protein_1 | - | - | - | - | 1.6 | 2.2 | - | - | - |
| DN2412_c0_g1_i27 |  | - | - | - | - | 4.89 | - | - | - | - |
| DN1361_c0_g1_i7 | Fatty-acid_amide_hydrolase_2 | - | - | - | - | -1.96 | - | - | -2.31 | - |
| DN2242_c0_g1_i8 | Probable_RNA-directed_DNA_polymerase_from_transposon_BS | - | - | - | - | -2.29 | -2.62 | - | - | - |
| DN12745_c0_g1_i2 | Probable_26S_proteasome_non-ATPase_regulatory_subunit_3 | - | - | - | - | -2.38 | - | - | - | - |
| DN4180_c0_g1_i8 |  | - | - | - | - | -1.71 | - | - | - | - |
| DN438_c1_g1_i1 |  | - | - | - | - | -1.64 | - | - | - | - |
| DN4082_c0_g1_i1 |  | - | - | - | - | -2.36 | - | - | - | - |
| DN1480_c0_g1_i5 |  | - | - | - | - | -3.75 | - | - | - | - |
| DN10373_c2_g1_i1 |  | - | - | - | - | - | 9.84 | - | 9.04 | - |
| DN1942_c0_g1_i6 | Cuticle_protein_19.8 | - | - | - | - | - | -12.88 | - | - | - |
| DN12537_c0_g2_i14 | Actin-5C | - | - | - | - | - | 11.45 | - | - | - |
| DN2048_c0_g1_i3 | Nucleosome_assembly_protein_1-like_1 | - | - | - | - | - | 12.7 | - | - | - |
| DN12537_c0_g2_i3 | Actin-5C | - | - | - | - | - | 11.72 | - | - | - |
| DN1942_c0_g1_i4 | Cuticle_protein_19.8 | - | - | - | - | - | -11.63 | - | -4.48 | - |
| DN1122_c0_g1_i3 | Facilitated_trehalose_transporter_Tret1 | - | - | - | - | - | 12.29 | - | 12.88 | - |
| DN4725_c0_g1_i15 | D-3-phosphoglycerate_dehydrogenase | - | - | - | - | - | 11.25 | - | 12.63 | - |
| DN1905_c0_g1_i18 |  | - | - | - | - | - | 11.33 | - | - | - |
| DN3906_c0_g1_i4 |  | - | - | - | - | - | -11.16 | - | - | - |
| DN2662_c0_g1_i10 | Activating_transcription_factor_of_chaperone | - | - | - | - | - | -12.07 | - | - | - |
| DN7113_c0_g1_i1 |  | - | - | - | - | - | 8.77 | - | - | - |
| DN2491_c0_g1_i3 |  | - | - | - | - | - | 11.08 | - | 11.36 | - |
| DN2976_c1_g1_i7 |  | - | - | - | - | - | -11.55 | - | - | - |
| DN1113_c0_g2_i6 |  | - | - | - | - | - | -11.54 | - | -11.63 | - |
| DN473_c2_g1_i19 | Disco-interacting_protein_2 | - | - | - | - | - | 11.48 | - | - | - |
| DN4788_c1_g1_i16 |  | - | - | - | - | - | 11.52 | - | 10.83 | - |
| DN379_c0_g1_i57 |  | - | - | - | - | - | 10.6 | - | - | - |
| DN513_c3_g1_i1 | PHD_finger_protein_rhinoceros | - | - | - | - | - | 11.8 | - | - | - |
| DN1048_c0_g1_i9 |  | - | - | - | - | - | 8.02 | - | - | - |
| DN2407_c0_g4_i10 |  | - | - | - | - | - | 10.8 | - | - | - |
| DN1660_c0_g1_i9 |  | - | - | - | - | - | 10.89 | - | - | - |
| DN2647_c0_g1_i13 | Phosphatidylinositol_4-kinase_alpha | - | - | - | - | - | 11.24 | - | 10.97 | - |
| DN4381_c0_g1_i3 |  | - | - | - | - | - | -8.73 | - | - | - |
| DN935_c0_g1_i11 |  | - | - | - | - | - | -10.81 | - | - | - |
| DN4617_c0_g2_i1 |  | - | - | - | - | - | 9.23 | - | - | - |
| DN1966_c0_g1_i3 |  | - | - | - | - | - | 3.86 | - | - | - |
| DN286_c0_g1_i12 |  | - | - | - | - | - | -3.12 | - | - | - |
| DN7103_c1_g1_i11 | Putative_inorganic_phosphate_cotransporter | - | - | - | - | - | -3.33 | - | - | - |
| DN2004_c1_g1_i4 |  | - | - | - | - | - | -2.19 | - | - | - |
| DN1670_c0_g1_i3 | Tubulin-specific_chaperone_cofactor_E-like_protein | - | - | - | - | - | -2.33 | - | - | - |
| DN28_c0_g1_i8 |  | - | - | - | - | - | -5.03 | - | -5.74 | - |
| DN328_c0_g1_i1 |  | - | - | - | - | - | 2.44 | - | - | - |
| DN1091_c0_g1_i11 |  | - | - | - | - | - | -2.64 | - | -2.41 | - |
| DN61_c0_g1_i2 | Cathepsin_L | - | - | - | - | - | - | -4.53 | - | - |
| DN2180_c0_g1_i5 |  | - | - | - | - | - | - | 11.62 | - | - |
| DN679_c0_g1_i7 |  | - | - | - | - | - | - | -10.84 | - | - |
| DN3035_c0_g2_i13 |  | - | - | - | - | - | - | -10.85 | - | - |
| DN1649_c0_g1_i3 |  | - | - | - | - | - | - | 10.4 | - | - |
| DN1519_c0_g1_i16 |  | - | - | - | - | - | - | 14.39 | - | - |
| DN1519_c0_g1_i5 |  | - | - | - | - | - | - | 12.11 | - | - |
| DN19_c0_g1_i20 | Protein_PRRC2B | - | - | - | - | - | - | - | -13.29 | - |
| DN40_c0_g1_i98 | Heat_shock_protein_83 | - | - | - | - | - | - | - | -12.69 | - |
| DN856_c0_g1_i19 |  | - | - | - | - | - | - | - | 11.82 | - |
| DN311_c0_g1_i7 |  | - | - | - | - | - | - | - | -10.01 | - |
| DN320_c0_g1_i6 | Facilitated_trehalose_transporter_Tret1 | - | - | - | - | - | - | - | 11.63 | - |
| DN59_c0_g1_i25 | Probable_C-mannosyltransferase_DPY19L1 | - | - | - | - | - | - | - | 11.5 | - |
| DN17090_c0_g1_i73 |  | - | - | - | - | - | - | - | -11.97 | - |
| DN515_c0_g1_i9 | Angiotensin-converting_enzyme-like_protein_Ace3 | - | - | - | - | - | - | - | 13.02 | - |
| DN7606_c0_g1_i5 |  | - | - | - | - | - | - | - | 11.33 | - |
| DN2179_c0_g2_i1 | Thioredoxin-related_transmembrane_protein_1 | - | - | - | - | - | - | - | -11.73 | - |
| DN1403_c0_g1_i1 |  | - | - | - | - | - | - | - | -11.63 | - |
| DN2484_c0_g1_i2 |  | - | - | - | - | - | - | - | 11.09 | - |
| DN1526_c0_g1_i9 |  | - | - | - | - | - | - | - | 11.43 | - |
| DN1082_c4_g1_i37 | CLIP-associating_protein | - | - | - | - | - | - | - | 11.41 | - |
| DN4721_c0_g1_i2 |  | - | - | - | - | - | - | - | 11.39 | - |
| DN7155_c0_g1_i2 |  | - | - | - | - | - | - | - | 10.64 | - |
| DN1400_c0_g1_i32 |  | - | - | - | - | - | - | - | 10.92 | - |
| DN3525_c0_g1_i4 |  | - | - | - | - | - | - | - | -11.11 | - |
| DN28_c1_g2_i2 |  | - | - | - | - | - | - | - | 10.56 | - |
| DN1403_c0_g1_i50 |  | - | - | - | - | - | - | - | 10.65 | - |
| DN28290_c0_g2_i13 |  | - | - | - | - | - | - | - | 10.38 | - |
| DN6517_c0_g1_i34 |  | - | - | - | - | - | - | - | 11.22 | - |
| DN946_c2_g2_i9 | Tetratricopeptide_repeat_protein_30A | - | - | - | - | - | - | - | 11.9 | 6.86 |
| DN1082_c4_g1_i38 | CLIP-associating_protein | - | - | - | - | - | - | - | 11.79 | - |
| DN7615_c0_g1_i15 |  | - | - | - | - | - | - | - | 7.98 | - |
| DN40_c0_g1_i118 |  | - | - | - | - | - | - | - | -12.51 | - |
| DN7155_c0_g1_i18 | Splicing_factor_45 | - | - | - | - | - | - | - | 11.84 | - |
| DN857_c0_g1_i12 |  | - | - | - | - | - | - | - | -2.77 | - |
| DN1488_c7_g1_i1 | SR-related_and_CTD-associated_factor_8 | - | - | - | - | - | - | - | 3.43 | - |
| DN354_c2_g2_i19 |  | - | - | - | - | - | - | - | -4.51 | - |
| DN16774_c0_g1_i6 | ATP-binding_cassette_sub-family_G_member_1 | - | - | - | - | - | - | - | -3.88 | - |
| DN3053_c1_g1_i2 |  | - | - | - | - | - | - | - | 3.71 | - |
| DN3623_c0_g1_i6 | Torso-like_protein | - | - | - | - | - | - | - | -2.15 | - |
| DN1153_c0_g1_i7 | ATP-dependent_Clp_protease_ATP-binding_subunit_clpX-like,_mitochondrial | - | - | - | - | - | - | - | 2.15 | - |
| DN496_c1_g1_i4 |  | - | - | - | - | - | - | - | -2.58 | - |
| DN1082_c1_g1_i2 |  | - | - | - | - | - | - | - | -11.06 | - |
| DN7879_c1_g1_i1 | Peptidyl-alpha-hydroxyglycine_alpha-amidating_lyase_1 | - | - | - | - | - | - | - | -2.04 | - |
| DN4045_c0_g1_i17 |  | - | - | - | - | - | - | - | -2.4 | - |
| DN5619_c1_g1_i5 |  | - | - | - | - | - | - | - | 3.19 | - |
| DN1908_c0_g2_i3 |  | - | - | - | - | - | - | - | -2.37 | - |
| DN354_c1_g1_i3 | Phosphatidate_phosphatase_LPIN3 | - | - | - | - | - | - | - | -2.24 | - |
| DN4113_c0_g1_i15 | Sequestosome-1 | - | - | - | - | - | - | - | -3.38 | - |
| DN1273_c0_g2_i2 |  | - | - | - | - | - | - | - | -3.07 | - |
| DN40_c0_g1_i24 | LIM_domain-containing_protein_WLIM2b | - | - | - | - | - | - | - | -4.18 | - |
| DN3551_c0_g1_i1 |  | - | - | - | - | - | - | - | -3.74 | - |
| DN16053_c1_g2_i6 |  | - | - | - | - | - | - | - | -2.25 | - |
| DN2503_c0_g1_i9 |  | - | - | - | - | - | - | - | -2.45 | - |
| DN1086_c0_g1_i2 | Angiotensin-converting_enzyme | - | - | - | - | - | - | - | -2.99 | - |
| DN598_c0_g1_i4 |  | - | - | - | - | - | - | - | -2.63 | - |
| DN11657_c0_g2_i2 | T-complex_protein_1_subunit_eta | - | - | - | - | - | - | - | -2.09 | - |
| DN4675_c1_g1_i1 |  | - | - | - | - | - | - | - | 3.58 | - |
| DN76_c0_g1_i14 |  | - | - | - | - | - | - | - | -2.73 | - |
| DN1215_c1_g1_i10 |  | - | - | - | - | - | - | - | -3.1 | - |
| DN616_c1_g1_i3 |  | - | - | - | - | - | - | - | -1.99 | - |
| DN12927_c0_g1_i1 | Muscle-specific_protein_20 | - | - | - | - | - | - | - | -2.25 | - |
| DN7810_c0_g4_i1 | Apolipoprotein_D | - | - | - | - | - | - | - | -2.36 | - |
| DN2737_c0_g1_i23 |  | - | - | - | - | - | - | - | -2.69 | - |
| DN3116_c1_g1_i15 |  | - | - | - | - | - | - | - | -1.79 | - |
| DN2846_c0_g1_i19 |  | - | - | - | - | - | - | - | -2.56 | - |
| DN297_c1_g1_i3 |  | - | - | - | - | - | - | - | -2.09 | - |
| DN6066_c0_g2_i2 |  | - | - | - | - | - | - | - | -2.18 | - |
| DN33440_c0_g1_i1 |  | - | - | - | - | - | - | - | -1.82 | - |
| DN1670_c0_g1_i6 | Tubulin-specific_chaperone_cofactor_E-like_protein | - | - | - | - | - | - | - | -2.08 | - |
| DN2846_c0_g1_i6 |  | - | - | - | - | - | - | - | -2.37 | - |
| DN320_c0_g1_i4 | Facilitated_trehalose_transporter_Tret1 | - | - | - | - | - | - | - | - | -12.52 |
| DN1774_c0_g1_i25 | GNBP | - | - | - | - | - | - | - | - | 14.33 |
| DN30991_c0_g1_i1 | Pelle | - | - | - | - | - | - | - | 1.33 | - |
| DN4239_c0_g1_i2 |  | - | - | - | - | - | - | - | - | -11.92 |
| DN1568_c0_g1_i2 |  | - | - | - | - | - | - | - | - | -11.45 |
| DN2981_c0_g1_i27 |  | - | - | - | - | - | - | - | - | 11.21 |
| DN887_c3_g1_i2 |  | - | - | - | - | - | - | - | - | 12.26 |
| DN8052_c0_g1_i1 |  | - | - | - | - | - | - | - | - | -11.5 |
| DN935_c0_g1_i8 |  | - | - | - | - | - | - | - | - | 11.06 |
| DN1653_c0_g1_i10 |  | - | - | - | - | - | - | - | - | 11.09 |
| DN30_c0_g2_i8 |  | - | - | - | - | - | - | - | - | 10.83 |
| DN18066_c0_g1_i7 |  | - | - | - | - | - | - | - | - | 10.34 |
| DN243_c0_g1_i9 | Inositol-trisphosphate_3-kinase_homolog | - | - | - | - | - | - | - | - | 5.17 |
| DN5323_c0_g1_i8 |  | - | - | - | - | - | - | - | - | -4.24 |
| DN320_c0_g1_i11 | Facilitated_trehalose_transporter_Tret1 | - | - | - | - | - | - | - | - | 2.49 |
| DN391_c1_g1_i2 |  | - | - | - | - | - | - | - | - | 5.04 |
| DN2423_c0_g1_i11 | Putative_inorganic_phosphate_cotransporter | - | - | - | - | - | - | - | - | 3.79 |
| DN1693_c0_g1_i2 | RNA-directed_DNA_polymerase_from_mobile_element_jockey | - | - | - | - | - | - | - | - | -2.01 |
| DN877_c3_g1_i1 |  | - | - | - | - | - | - | - | - | -3.54 |
| DN1006_c0_g1_i6 | Protein_spinster_homolog_1 | - | - | - | - | - | - | - | - | 4.83 |
| DN260_c3_g1_i8 | Apolipophorins | - | - | - | - | - | - | - | - | -2.34 |
| DN6_c0_g1_i3 |  | - | - | - | - | - | - | - | - | -2.68 |
| DN2173_c1_g1_i6 | Aromatic-L-amino-acid_decarboxylase | - | - | - | - | - | - | - | - | 4.9 |
| DN521_c1_g1_i6 |  | - | - | - | - | - | - | - | - | -3.95 |
| DN20_c2_g2_i1 | Defensin | - | - | - | - | - | - | - | - | -3.05 |
| DN1186_c0_g1_i35 | Lysosomal-associated_transmembrane_protein_4A | - | - | - | - | - | - | - | - | -1.46 |
| DN1774_c0_g1_i15 | GNBP | - | - | - | - | - | - | - | - | 8.13 |
| DN1032_c1_g1_i5 |  | - | - | - | - | - | - | - | - | -2.08 |
| DN11689_c0_g1_i5 | Acidic_mammalian_chitinase | - | - | - | - | - | - | - | - | 4.01 |
| DN1144_c0_g1_i20 |  | - | - | - | - | - | - | - | - | 2.71 |
| DN3300_c0_g1_i9 |  | - | - | - | - | - | - | - | - | 4.15 |
| DN3262_c0_g1_i3 | Serine_protease_inhibitor_88Ea/ rpSPI10b | - | - | - | - | - | - | - | - | 3.42 |
| DN7454_c0_g1_i28 |  | - | - | - | - | - | - | - | - | 8.43 |
| DN2903_c2_g1_i6 | Amidophosphoribosyltransferase | - | - | - | - | - | - | - | - | -2.7 |
| DN1519_c0_g1_i40 |  | - | - | - | - | - | - | - | - | -3.21 |
| DN13091_c0_g1_i3 | Chitotriosidase | - | - | - | - | - | - | - | - | 4.81 |
| DN1986_c0_g1_i9 | Sodium-dependent_nutrient_amino_acid_transporter_1 | - | - | - | - | - | - | - | - | 2.21 |
| DN2418_c0_g1_i1 |  | - | - | - | - | - | - | - | - | -5.05 |
| DN1412_c0_g3_i1 |  | - | - | - | - | - | - | - | - | -3.77 |
| DN13436_c0_g1_i5 | ATP-binding_cassette_sub-family_D_member | - | - | - | - | - | - | - | - | -1.4 |
| DN378_c0_g1_i14 |  | - | - | - | - | - | - | - | - | -2.11 |
| DN18770_c0_g1_i6 |  | - | - | - | - | - | - | - | - | -1.61 |
| DN3211_c1_g1_i2 |  | - | - | - | - | - | - | - | - | -5.71 |
| DN7352_c0_g1_i1 |  | - | - | - | - | - | - | - | - | -1.87 |
| DN4056_c0_g1_i20 | Organic_cation_transporter_protein | - | - | - | - | - | - | - | - | -2.13 |
| DN2874_c0_g1_i3 |  | - | - | - | - | - | - | - | - | -2.15 |
| DN257_c0_g1_i10 |  | - | - | - | - | - | - | - | - | 1.99 |
| DN2059_c0_g1_i5 |  | - | - | - | - | - | - | - | - | -1.93 |
| DN297_c0_g2_i27 | Serine_protease_snake/rpSP30 | - | - | - | - | - | - | - | - | -2.45 |

Table S2. List of *Rhodnius prolixus* immune transcripts used for differential expression analyses. Transcripts with known or predicted immune functions are listed and categorized according to their function or molecular pathway. Expression values used to create heatmaps of Figs 2 and 3 are listed at the end of the table. Corresponding Vector Base IDs were found for each transcript with BLASTn, sequences with <95% identity or <70% coverage are listed as unnanotated (NA).

| ID | Category | Name | Vector Base  ID | Log2 (TMM-Treatment/(AVERAGE(TMM-All-treatments) | | | | | |  |
| --- | --- | --- | --- | --- | --- | --- | --- | --- | --- | --- |
|  |  |  |  | **PBS.8** | **Ec.8** | **Sa.8** | **PBS.24** | **Ec.24** | **Sa.24** | |
| DN1239_c1_g1_i13 | Antimicrobial peptides | Lysozyme | NA | 0.28 | -1.29 | 0.97 | 0.01 | 0.26 | -2.26 | |
| DN1239_c1_g1_i18 | Antimicrobial peptides | Lysozyme | RPRC015442 | 0.10 | -0.73 | 1.03 | -0.54 | 0.29 | -1.42 | |
| DN1239_c1_g1_i21 | Antimicrobial peptides | Lysozyme | RPRC015442 | -0.20 | 0.32 | -0.80 | 0.39 | -1.05 | 0.60 | |
| DN1239_c1_g1_i24 | Antimicrobial peptides | Lysozyme | RPRC015442 | -0.77 | 0.81 | -1.19 | 0.23 | -0.16 | 0.21 | |
| DN1239_c1_g1_i29 | Antimicrobial peptides | Lysozyme | RPRC015442 | -0.76 | 0.92 | -1.52 | 0.20 | -0.19 | 0.19 | |
| DN1239_c1_g1_i32 | Antimicrobial peptides | Lysozyme | RPRC015442 | 0.09 | 0.38 | -2.09 | 0.09 | -0.67 | 0.77 | |
| DN1239_c1_g1_i35 | Antimicrobial peptides | Lysozyme | RPRC015442 | 0.91 | -0.72 | 0.53 | -0.80 | -0.58 | -0.27 | |
| DN1239_c1_g1_i37 | Antimicrobial peptides | Lysozyme | RPRC015442 | 0.92 | -0.70 | 0.51 | -0.74 | -0.53 | -0.37 | |
| DN1239_c1_g1_i40 | Antimicrobial peptides | Lysozyme | RPRC015442 | 0.12 | -0.37 | 0.73 | -0.36 | 0.30 | -1.09 | |
| DN1239_c1_g1_i43 | Antimicrobial peptides | Lysozyme | RPRC015442 | 0.17 | -0.30 | 0.76 | -1.39 | 0.52 | -0.86 | |
| DN1239_c1_g1_i44 | Antimicrobial peptides | Lysozyme | RPRC015442 | -0.01 | -0.63 | 0.35 | 1.29 | -0.74 | -4.60 | |
| DN1844_c0_g1_i1 | Antimicrobial peptides | Defensin-B | RPRC004803 | -0.03 | 0.10 | -0.32 | -0.30 | 0.02 | 0.40 | |
| DN1844_c0_g1_i11 | Antimicrobial peptides | Defensin-B | RPRC004803 | 0.09 | 0.05 | 0.00 | -0.01 | -0.02 | -0.12 | |
| DN1844_c0_g1_i2 | Antimicrobial peptides | Defensin-B | RPRC004803 | 0.17 | 0.05 | 0.11 | 0.05 | -0.02 | -0.43 | |
| DN1844_c0_g1_i3 | Antimicrobial peptides | Defensin-B | RPRC004803 | -1.57 | 0.70 | -0.72 | 0.40 | 0.70 | -1.05 | |
| DN1844_c0_g1_i6 | Antimicrobial peptides | Defensin-B | RPRC004803 | 0.17 | -0.09 | -0.61 | 0.12 | 0.51 | -0.35 | |
| DN20_c2_g2_i1 | Antimicrobial peptides | Defensin-C | RPRC012184 | -1.59 | 0.60 | 0.39 | 1.39 | -3.97 | -2.69 | |
| DN20_c3_g1_i15 | Antimicrobial peptides | Defensin-B | RPRC004803 | 0.53 | -1.40 | -0.05 | 0.47 | #NUM! | 0.87 | |
| DN20_c3_g1_i2 | Antimicrobial peptides | Defensin-B | RPRC012186 | 0.00 | 0.45 | 0.52 | 0.07 | -0.55 | -1.10 | |
| DN20_c3_g1_i3 | Antimicrobial peptides | Defensin-B | RPRC004803 | -0.07 | -0.26 | -0.07 | 0.26 | 0.48 | -0.58 | |
| DN20_c3_g1_i8 | Antimicrobial peptides | Defensin-B | RPRC004803 | 0.04 | -0.20 | 0.27 | -0.06 | 0.61 | -1.31 | |
| DN2263_c0_g1_i10 | Antimicrobial peptides | Defensin- | RPRC012186 | 0.62 | -1.05 | 0.63 | -0.22 | -0.11 | -0.63 | |
| DN2263_c0_g1_i11 | Antimicrobial peptides | Defensin | RPRC004803 | -0.51 | -0.16 | -0.70 | 0.51 | 0.22 | 0.26 | |
| DN2263_c0_g1_i13 | Antimicrobial peptides | Defensin-B | RPRC012182 | -2.09 | 0.12 | -0.42 | 0.49 | 0.06 | 0.58 | |
| DN2263_c0_g1_i2 | Antimicrobial peptides | Defensin-B | RPRC012182 | -0.54 | 0.01 | -0.62 | 0.61 | 0.18 | 0.00 | |
| DN2263_c0_g1_i20 | Antimicrobial peptides | Defensin | RPRC012186 | 0.07 | -0.85 | -0.31 | -0.03 | 0.38 | 0.39 | |
| DN2263_c0_g1_i23 | Antimicrobial peptides | Defensin | RPRC004804 | -1.67 | -1.36 | 0.52 | -0.44 | 0.44 | 0.83 | |
| DN2263_c0_g1_i4 | Antimicrobial peptides | Defensin | RPRC012186 | -0.65 | 0.07 | -0.73 | 0.76 | 0.12 | -0.10 | |
| DN2263_c0_g1_i7 | Antimicrobial peptides | Defensin | RPRC012186 | 0.03 | 0.08 | -0.20 | -0.13 | -0.30 | 0.41 | |
| DN2263_c0_g1_i9 | Antimicrobial peptides | Defensin-B | RPRC012182 | 0.08 | 0.12 | -0.17 | -0.02 | -0.21 | 0.15 | |
| DN2263_c0_g2_i1 | Antimicrobial peptides | Defensin | RPRC012177 | -1.85 | 0.07 | -0.96 | 1.14 | -0.09 | 0.01 | |
| DN2263_c0_g2_i2 | Antimicrobial peptides | Defensin | RPRC012177 | -1.82 | 0.10 | -0.99 | 1.24 | -0.02 | -0.34 | |
| DN27360_c0_g1_i4 | Antimicrobial peptides | Defensin-B | RPRC012182 | -0.53 | 0.42 | -0.63 | 0.01 | -0.23 | 0.55 | |
| DN3991_c0_g2_i1 | Antimicrobial peptides | Prolixicin | RPRC013002 | 0.34 | -0.22 | -1.86 | -0.87 | 0.25 | 0.90 | |
| DN503_c0_g1_i6 | Antimicrobial peptides | Defensin | RPRC012259 | 0.15 | -0.07 | 0.79 | -0.25 | 0.44 | -5.72 | |
| DN503_c0_g1_i7 | Antimicrobial peptides | Defensin | RPRC012177 | 0.27 | -0.07 | 0.29 | 0.42 | -0.18 | -1.33 | |
| DN503_c2_g1_i2 | Antimicrobial peptides | Defensin | RPRC012180 | -0.42 | 0.82 | -1.27 | 0.44 | 0.20 | -0.81 | |
| DN503_c2_g1_i3 | Antimicrobial peptides | Defensin | RPRC012180 | -0.63 | 0.66 | 0.93 | -0.68 | -1.49 | -0.17 | |
| DN7656_c0_g2_i1 | Antimicrobial peptides | Lysozyme | RPRC015441 | -1.18 | -0.54 | -0.10 | 0.66 | -0.10 | 0.51 | |
| DN8775_c0_g1_i10 | Antimicrobial peptides | Lysozyme | RPRC015440 | 0.01 | 0.10 | -0.06 | 0.93 | -0.59 | -1.36 | |
| DN8775_c0_g1_i2 | Antimicrobial peptides | Lysozyme | RPRC015440 | 0.59 | 0.12 | 0.08 | -0.70 | -3.11 | 0.70 | |
| DN8775_c0_g1_i9 | Antimicrobial peptides | Lysozyme | RPRC015440 | 0.47 | -0.80 | -0.77 | -0.32 | -0.21 | 0.83 | |
| DN20852_c0_g1_i1 | Autophagy related genes | Beclin | R4G472/ RPRC006439 | 0.05 | -0.15 | -0.06 | 0.21 | 0.08 | -0.17 | |
| DN20852_c0_g1_i2 | Autophagy related genes | Beclin | R4G472/ RPRC006439 | -0.36 | 0.98 | -0.64 | -0.69 | 0.18 | -0.24 | |
| DN20852_c0_g1_i4 | Autophagy related genes | Beclin | R4G472/ RPRC002353 | -0.08 | -0.31 | 0.57 | 0.42 | -0.65 | -0.34 | |
| DN474_c6_g1_i1 | Autophagy related genes | Atg13-like | T1I7M3/ RPRC012295 | -0.20 | 0.17 | -0.73 | 0.10 | -0.48 | 0.69 | |
| DN474_c6_g1_i2 | Autophagy related genes | Atg13-like | T1I7M3/ RPRC012295 | -0.13 | 0.06 | -0.84 | 0.51 | -0.14 | 0.21 | |
| DN5364_c0_g1_i2 | Autophagy related genes | Atg5-like | R4FNU1/ RPRC014466 | 1.17 | -0.47 | 0.39 | -0.60 | -1.41 | -0.55 | |
| DN5364_c0_g1_i4 | Autophagy related genes | Atg5-like | R4FNU1/ RPRC014466 | -0.29 | -0.61 | 0.82 | -0.40 | 0.62 | -1.09 | |
| DN5364_c0_g1_i5 | Autophagy related genes | Atg5-like | R4FNU1/ RPRC014466 | -0.52 | -0.38 | -2.02 | 0.42 | -0.10 | 1.01 | |
| DN5364_c0_g1_i7 | Autophagy related genes | Atg5-like | R4FNU1/ RPRC014466 | -0.02 | 0.03 | -0.26 | -0.04 | 0.48 | -0.35 | |
| DN6523_c0_g2_i1 | Autophagy related genes | Atg3-like | R4FKK4/ RPRC008742 | 0.23 | -0.16 | 0.10 | 0.04 | 0.05 | -0.34 | |
| DN9020_c0_g3_i1 | Catalases | Catalase | RPRC009934 | -0.34 | -0.08 | 0.23 | 0.19 | 0.09 | -0.19 | |
| DN9001_c0_g1_i1 | Coagulation | Hemolectin | RPRC015366 | 0.22 | -0.16 | 0.39 | -0.08 | 0.12 | -0.76 | |
| DN9001_c0_g1_i3 | Coagulation | Hemolectin | RPRC015366 | -0.09 | -0.08 | 0.18 | -0.15 | 0.20 | -0.11 | |
| DN1118_c2_g1_i1 | IMD signaling pathway | Tak1 | RPRC014191 | 1.24 | 1.13 | -0.25 | -2.26 | -1.29 | #NUM! | |
| DN1118_c2_g1_i2 | IMD signaling pathway | Tak1 | RPRC014214 | -1.05 | -0.30 | -0.15 | 0.98 | 0.42 | -1.02 | |
| DN139_c0_g2_i1 | IMD signaling pathway | DREDD | GECK01002741.1/ RPRC009850 | -1.26 | -0.15 | -0.90 | 0.70 | 0.06 | 0.56 | |
| DN139_c0_g2_i13 | IMD signaling pathway | DREDD | GECK01002741.1/RPRC009850 | -0.53 | 0.43 | -0.08 | 0.30 | -0.23 | -0.11 | |
| DN139_c0_g2_i14 | IMD signaling pathway | DREDD | GECK01002741.1/RPRC009850 | 0.20 | -0.27 | -0.05 | -0.30 | -0.11 | 0.39 | |
| DN139_c0_g2_i20 | IMD signaling pathway | DREDD | GECK01002741.1/RPRC009850 | 0.85 | -0.72 | 0.17 | -0.35 | -0.53 | -0.03 | |
| DN139_c0_g2_i23 | IMD signaling pathway | DREDD | GECK01002741.1/RPRC009850 | -0.34 | -0.99 | 0.12 | -0.78 | 1.22 | -0.49 | |
| DN139_c0_g2_i24 | IMD signaling pathway | DREDD | GECK01002741.1/RPRC009850 | -0.44 | -0.93 | 0.09 | -0.69 | 1.29 | -0.71 | |
| DN139_c0_g2_i3 | IMD signaling pathway | DREDD | GECK01002741.1/RPRC009850 | 1.24 | -2.89 | 0.67 | -5.02 | 0.14 | -0.35 | |
| DN139_c0_g2_i5 | IMD signaling pathway | DREDD | GECK01002741.1/RPRC009850 | 0.67 | -0.75 | 0.98 | -1.43 | -1.27 | 0.07 | |
| DN139_c0_g2_i8 | IMD signaling pathway | DREDD | GECK01002741.1/RPRC009850 | 0.06 | -0.03 | -0.26 | -0.16 | -0.07 | 0.37 | |
| DN1528_c0_g1_i1 | IMD signaling pathway | Uev1A | RPRC011375 | -0.06 | 0.07 | -0.38 | 0.05 | -0.06 | 0.30 | |
| DN1528_c0_g1_i3 | IMD signaling pathway | Uev1A | RPRC011375 | -0.11 | 0.20 | -0.08 | 0.34 | 0.03 | -0.53 | |
| DN1528_c0_g1_i5 | IMD signaling pathway | Uev1A | RPRC011375 | 0.25 | -0.04 | 0.23 | 0.38 | -0.17 | -1.07 | |
| DN20238_c0_g1_i2 | IMD signaling pathway | Dnr1 | RPRC011962 | 0.18 | 0.17 | 0.07 | 0.08 | -1.14 | 0.23 | |
| DN20238_c0_g1_i4 | IMD signaling pathway | Dnr1 | RPRC011962 | -0.56 | 1.13 | 0.46 | 0.40 | -2.83 | -1.74 | |
| DN20238_c0_g1_i5 | IMD signaling pathway | Dnr1 | RPRC011962 | -0.29 | 0.28 | -0.07 | 0.41 | -0.70 | 0.09 | |
| DN20238_c0_g1_i6 | IMD signaling pathway | Dnr1 | RPRC011962 | 0.18 | 0.06 | -0.28 | 0.09 | -0.01 | -0.08 | |
| DN2045_c0_g1_i11 | IMD signaling pathway | Dnr1 | NA | 0.71 | -0.91 | 0.36 | 0.39 | -0.28 | -1.26 | |
| DN3894_c0_g3_i16 | IMD signaling pathway | Relish | RPRC017358 | 0.00 | -0.14 | -0.53 | 0.05 | -0.12 | 0.53 | |
| DN3894_c0_g3_i20 | IMD signaling pathway | Relish | RPRC017358 | 0.03 | -0.08 | -0.51 | 0.16 | -0.04 | 0.32 | |
| DN3894_c0_g3_i23 | IMD signaling pathway | Relish | RPRC017358 | 0.66 | -0.40 | 0.55 | -0.13 | -0.33 | -1.03 | |
| DN3894_c0_g3_i27 | IMD signaling pathway | Relish | RPRC017358 | 0.64 | -0.09 | 0.14 | -0.21 | 0.23 | -1.44 | |
| DN3894_c0_g3_i5 | IMD signaling pathway | Relish | RPRC017358 | 0.56 | -0.63 | 1.08 | 0.08 | -1.14 | -2.02 | |
| DN3894_c0_g3_i6 | IMD signaling pathway | Relish | RPRC017358 | -0.43 | -0.20 | -0.43 | 0.03 | 0.05 | 0.67 | |
| DN3894_c0_g3_i9 | IMD signaling pathway | Relish | RPRC017358 | -0.06 | -0.22 | 0.27 | -0.03 | 0.22 | -0.25 | |
| DN4172_c0_g2_i1 | IMD signaling pathway | FADD | RPRC013858 | 0.42 | -1.12 | 0.35 | #NUM! | 0.50 | 0.60 | |
| DN4172_c0_g2_i2 | IMD signaling pathway | FADD | NA | 0.11 | 0.25 | 0.14 | -0.01 | -0.13 | -0.47 | |
| DN4172_c0_g2_i3 | IMD signaling pathway | FADD | RPRC013858 | -0.67 | 0.62 | -0.79 | 0.53 | -0.80 | 0.31 | |
| DN4180_c0_g1_i1 | IMD signaling pathway | IMD | RPRC003895 | -0.24 | -0.26 | -0.05 | 0.52 | -0.12 | 0.00 | |
| DN4180_c0_g1_i3 | IMD signaling pathway | IMD | RPRC003895 | -0.47 | 0.32 | 0.02 | 0.56 | 0.34 | -1.83 | |
| DN4180_c0_g1_i4 | IMD signaling pathway | IMD | RPRC003895 | -0.05 | 1.14 | -2.68 | -0.15 | -0.60 | 0.15 | |
| DN4180_c0_g1_i6 | IMD signaling pathway | IMD | RPRC003895 | 0.28 | 0.46 | -0.91 | -0.01 | 0.11 | -0.32 | |
| DN4180_c0_g1_i7 | IMD signaling pathway | IMD | RPRC003895 | 0.13 | -0.58 | -0.33 | -0.05 | -0.06 | 0.61 | |
| DN4180_c0_g1_i8 | IMD signaling pathway | IMD | RPRC003895 | 0.17 | -1.11 | -0.49 | 0.33 | 0.17 | 0.39 | |
| DN443_c0_g3_i1 | IMD signaling pathway | Caspar | NA | -0.37 | 0.05 | -0.63 | 0.67 | -0.19 | 0.11 | |
| DN443_c0_g3_i3 | IMD signaling pathway | Caspar | NA | 0.36 | -0.48 | -0.18 | 0.00 | -0.29 | 0.37 | |
| DN443_c0_g3_i4 | IMD signaling pathway | Caspar | NA | 0.39 | -0.76 | -0.01 | 0.16 | -0.08 | 0.06 | |
| DN443_c0_g3_i5 | IMD signaling pathway | Caspar | RPRC001459 | 0.12 | 0.06 | -0.26 | -0.30 | -0.06 | 0.34 | |
| DN443_c0_g3_i6 | IMD signaling pathway | Caspar | NA | -0.20 | 1.09 | -0.71 | 0.27 | -0.50 | -1.06 | |
| DN4913_c0_g2_i1 | IMD signaling pathway | CYLD | GECK01020525.1/RPRC01466 | 0.51 | -1.27 | 1.43 | -0.95 | -0.43 | -2.35 | |
| DN4913_c0_g2_i3 | IMD signaling pathway | CYLD | GECK01020525.1/RPRC01466 | -0.58 | 0.75 | -0.45 | 0.82 | -2.22 | -0.08 | |
| DN5857_c0_g1_i3 | IMD signaling pathway | IkkB | GECK01053880.1 | 0.69 | -1.18 | -1.14 | -0.33 | -0.08 | 0.81 | |
| DN5857_c0_g1_i6 | IMD signaling pathway | IkkB | GECK01053880.1 | -0.13 | -1.01 | -2.70 | -0.02 | -0.74 | 1.51 | |
| DN5857_c0_g1_i7 | IMD signaling pathway | IkkB | GECK01053880.1 | 0.19 | -0.47 | 0.89 | -0.48 | 0.26 | -1.44 | |
| DN5857_c0_g1_i8 | IMD signaling pathway | IkkB | GECK01053880.1 | -0.32 | 0.01 | -0.21 | 0.43 | 0.37 | -0.54 | |
| DN613_c2_g1_i1 | IMD signaling pathway | Ubc5 | RPRC005317 | 0.15 | -0.04 | 0.27 | -0.06 | 0.09 | -0.54 | |
| DN613_c2_g1_i2 | IMD signaling pathway | Ubc5 | RPRC005317 | 0.21 | -0.19 | 0.37 | -0.12 | 0.24 | -0.81 | |
| DN613_c2_g1_i3 | IMD signaling pathway | Ubc5 | RPRC005317 | -0.63 | 0.71 | -0.19 | 0.76 | -0.91 | -0.71 | |
| DN613_c2_g1_i4 | IMD signaling pathway | Ubc5 | RPRC005317 | 0.14 | 0.05 | 0.17 | 0.08 | 0.07 | -0.66 | |
| DN613_c2_g1_i5 | IMD signaling pathway | Ubc5 | RPRC005317 | 0.26 | -0.71 | -0.44 | -0.60 | -0.21 | 0.95 | |
| DN613_c2_g1_i6 | IMD signaling pathway | Ubc5 | RPRC005317 | 0.29 | -0.47 | -0.14 | -0.17 | -0.09 | 0.40 | |
| DN6671_c0_g1_i2 | IMD signaling pathway | Iap2 | RPRC007068 | -0.39 | -0.08 | 0.19 | -0.25 | 0.22 | 0.19 | |
| DN6671_c0_g1_i3 | IMD signaling pathway | Iap2 | RPRC007068 | -0.71 | 1.04 | -0.65 | -0.08 | 0.08 | -0.53 | |
| DN6671_c0_g1_i4 | IMD signaling pathway | Iap2 | RPRC007068 | 0.12 | -0.09 | 0.39 | -0.12 | 0.08 | -0.55 | |
| DN671_c0_g1_i10 | IMD signaling pathway | Iap2 | RPRC005602 | 0.15 | -0.22 | 0.24 | -0.28 | 0.57 | -0.88 | |
| DN671_c0_g1_i3 | IMD signaling pathway | Iap2 | RPRC00560 | 0.12 | -0.05 | 0.38 | 0.00 | 0.04 | -0.70 | |
| DN671_c0_g1_i6 | IMD signaling pathway | Iap2 | RPRC00560 | 0.19 | -0.11 | 0.08 | -0.04 | 0.03 | -0.19 | |
| DN671_c0_g1_i7 | IMD signaling pathway | Iap2 | RPRC00560 | -0.58 | -0.21 | -1.36 | -0.66 | 0.81 | 0.76 | |
| DN7123_c0_g1_i1 | IMD signaling pathway | Ubc13 (aka Bendless) | RPRC011790 | 0.35 | -0.40 | 0.37 | -0.29 | 0.44 | -0.99 | |
| DN1362_c2_g1_i2 | JAK/STAT signaling pathway | Shp | RPRC004483 | -0.10 | -0.14 | 0.70 | -0.25 | 0.04 | -0.59 | |
| DN1362_c2_g1_i3 | JAK/STAT signaling pathway | Shp | RPRC004483 | 0.48 | -0.28 | -0.09 | -0.34 | -0.29 | 0.30 | |
| DN1362_c2_g1_i4 | JAK/STAT signaling pathway | Shp | RPRC004483 | 0.79 | -0.46 | 0.08 | -0.41 | -0.08 | -0.34 | |
| DN3270_c0_g1_i11 | JAK/STAT signaling pathway | Peroxidasin | RPRC014176 | -1.77 | -0.35 | -0.77 | 0.70 | 0.24 | 0.61 | |
| DN3270_c0_g1_i2 | JAK/STAT signaling pathway | Peroxidasin | RPRC004965 | 0.03 | 0.06 | 0.45 | -0.11 | -0.06 | -0.56 | |
| DN3270_c0_g1_i3 | JAK/STAT signaling pathway | Peroxidasin | RPRC004965 | -0.12 | -0.27 | 0.30 | 0.11 | 0.45 | -0.80 | |
| DN3270_c0_g1_i4 | JAK/STAT signaling pathway | Peroxidasin | RPRC005333 | 0.37 | -0.54 | 1.13 | -0.51 | 0.13 | -4.72 | |
| DN3270_c0_g1_i5 | JAK/STAT signaling pathway | Peroxidasin | RPRC004965 | -0.23 | 0.30 | 0.09 | 0.02 | -0.10 | -0.15 | |
| DN3270_c0_g1_i6 | JAK/STAT signaling pathway | Peroxidasin | RPRC004965 | 0.23 | 0.25 | -0.03 | 0.26 | -0.20 | -0.76 | |
| DN3270_c0_g1_i8 | JAK/STAT signaling pathway | Peroxidasin | RPRC004965 | -0.25 | 0.84 | 0.14 | 0.41 | -2.23 | -0.47 | |
| DN4122_c0_g1_i1 | JAK/STAT signaling pathway | Stat | RPRC006502 | -0.01 | -0.01 | 0.11 | 0.19 | -0.30 | -0.03 | |
| DN4122_c0_g1_i2 | JAK/STAT signaling pathway | Stat | RPRC006502 | 0.16 | -0.22 | -0.34 | 0.01 | -0.11 | 0.39 | |
| DN4122_c0_g1_i3 | JAK/STAT signaling pathway | Stat | RPRC006502 | 0.65 | -1.07 | 1.14 | -1.00 | -0.23 | -1.33 | |
| DN4845_c0_g1_i1 | JAK/STAT signaling pathway | Cbl | RPRC010957 | -0.06 | 0.17 | -0.68 | 0.20 | -0.23 | 0.36 | |
| DN4845_c0_g1_i2 | JAK/STAT signaling pathway | Cbl | RPRC010957 | -0.42 | -0.38 | 0.07 | 0.14 | 0.36 | 0.07 | |
| DN4845_c0_g1_i3 | JAK/STAT signaling pathway | Cbl | RPRC010957 | -1.85 | 0.77 | 0.04 | 0.08 | -0.72 | 0.40 | |
| DN6081_c2_g1_i14 | JAK/STAT signaling pathway | Socs | RPRC002037 | -0.07 | -2.28 | 0.39 | 0.67 | 0.43 | -0.75 | |
| DN6081_c2_g1_i18 | JAK/STAT signaling pathway | Socs | RPRC002037 | -0.37 | -0.07 | 0.13 | 0.15 | -0.35 | 0.36 | |
| DN6081_c2_g1_i6 | JAK/STAT signaling pathway | Socs | RPRC002037 | 0.18 | 0.28 | -0.13 | 0.02 | -0.19 | -0.24 | |
| DN63_c0_g1_i10 | JAK/STAT signaling pathway | Pias | RPRC002055 | 1.10 | -0.25 | -1.68 | 0.10 | -0.71 | 0.03 | |
| DN63_c0_g1_i11 | JAK/STAT signaling pathway | Pias | RPRC002055 | 0.32 | -0.68 | -0.40 | -0.54 | -0.14 | 0.83 | |
| DN63_c0_g1_i13 | JAK/STAT signaling pathway | Pias | RPRC002055 | 0.46 | -0.04 | 0.17 | 0.24 | -0.40 | -0.78 | |
| DN63_c0_g1_i14 | JAK/STAT signaling pathway | Pias | RPRC002055 | 0.46 | -0.03 | 0.12 | 0.29 | -0.34 | -0.88 | |
| DN63_c0_g1_i15 | JAK/STAT signaling pathway | Pias | RPRC002055 | 0.22 | -0.05 | 0.25 | -0.08 | -0.01 | -0.44 | |
| DN63_c0_g1_i16 | JAK/STAT signaling pathway | Pias | RPRC002055 | 0.23 | -0.03 | 0.23 | 0.00 | 0.04 | -0.63 | |
| DN63_c0_g1_i17 | JAK/STAT signaling pathway | Pias | RPRC002055 | -0.02 | 0.12 | -0.22 | -0.06 | 0.00 | 0.15 | |
| DN63_c0_g1_i18 | JAK/STAT signaling pathway | Pias | RPRC002055 | 0.24 | -0.18 | 0.26 | -0.18 | -0.09 | -0.12 | |
| DN63_c0_g1_i2 | JAK/STAT signaling pathway | Pias | RPRC002055 | -0.77 | 0.52 | -0.34 | -0.15 | 0.28 | 0.11 | |
| DN63_c0_g1_i4 | JAK/STAT signaling pathway | Pias | RPRC002055 | -0.08 | 0.25 | -0.38 | 0.06 | 0.10 | -0.04 | |
| DN63_c0_g1_i5 | JAK/STAT signaling pathway | Pias | RPRC002055 | 0.12 | -0.08 | 0.12 | 0.02 | 0.07 | -0.27 | |
| DN63_c0_g1_i6 | JAK/STAT signaling pathway | Pias | RPRC002055 | 0.26 | 0.39 | -0.34 | -0.24 | -0.68 | 0.29 | |
| DN7956_c0_g1_i1 | JAK/STAT signaling pathway | Cis | RPRC001962 | 0.02 | 0.01 | -0.21 | 0.24 | -0.02 | -0.08 | |
| DN7956_c0_g1_i2 | JAK/STAT signaling pathway | Cis | RPRC001962 | -0.13 | -0.07 | -0.07 | 0.17 | 0.13 | -0.06 | |
| DN7956_c0_g1_i3 | JAK/STAT signaling pathway | Cis | RPRC001962 | 1.58 | -1.11 | -3.41 | -3.22 | -0.07 | 0.48 | |
| DN7956_c0_g1_i4 | JAK/STAT signaling pathway | Cis | RPRC001962 | -1.33 | 0.11 | -0.67 | 0.99 | 0.24 | -0.46 | |
| DN7956_c0_g1_i5 | JAK/STAT signaling pathway | Cis | RPRC001962 | -0.51 | -0.44 | -0.34 | 0.67 | 0.35 | -0.15 | |
| DN7956_c0_g1_i6 | JAK/STAT signaling pathway | Cis | RPRC001962 | -1.89 | -0.21 | -0.81 | 1.37 | -0.44 | -0.04 | |
| DN7956_c0_g1_i8 | JAK/STAT signaling pathway | Cis | RPRC001962 | -0.10 | -0.02 | -0.11 | 0.25 | 0.21 | -0.31 | |
| DN818_c0_g1_i18 | JAK/STAT signaling pathway | Stam | RPRC015396 | 0.12 | -0.28 | 0.48 | -0.08 | -0.32 | -0.07 | |
| DN818_c0_g1_i2 | JAK/STAT signaling pathway | Stam | RPRC015396 | 0.75 | 0.12 | 0.06 | -0.04 | -1.01 | -0.46 | |
| DN818_c0_g1_i21 | JAK/STAT signaling pathway | Stam | RPRC015396 | 0.00 | -0.43 | 0.07 | 0.15 | 0.07 | 0.07 | |
| DN818_c0_g1_i22 | JAK/STAT signaling pathway | Stam | RPRC015396 | 0.20 | -1.04 | 0.80 | -1.48 | 0.54 | -0.30 | |
| DN818_c0_g1_i23 | JAK/STAT signaling pathway | Stam | RPRC015396 | 0.00 | -0.05 | 0.02 | 0.03 | 0.01 | -0.01 | |
| DN818_c0_g1_i25 | JAK/STAT signaling pathway | Stam | RPRC015396 | 0.18 | -0.71 | -1.13 | 0.45 | 0.19 | 0.37 | |
| DN818_c0_g1_i27 | JAK/STAT signaling pathway | Stam | RPRC015396 | -0.28 | 0.12 | -0.68 | 0.64 | 0.05 | -0.20 | |
| DN818_c0_g1_i29 | JAK/STAT signaling pathway | Stam | RPRC015396 | -1.12 | #NUM! | 0.51 | -0.82 | 0.87 | 0.78 | |
| DN818_c0_g1_i30 | JAK/STAT signaling pathway | Stam | RPRC015396 | 0.03 | -0.01 | 0.05 | 0.10 | 0.06 | -0.26 | |
| DN818_c0_g1_i4 | JAK/STAT signaling pathway | Stam | RPRC015396 | -0.21 | -0.46 | 0.39 | -0.19 | 0.31 | -0.03 | |
| DN9762_c0_g1_i1 | JAK/STAT signaling pathway | Jak | RPRC012987 | 0.24 | 0.43 | -0.17 | -0.05 | -0.40 | -0.21 | |
| DN1082_c4_g1_i1 | NA | CLIP-associating protein | RPRC005737 | 0.28 | -0.06 | 0.48 | -0.45 | 0.28 | -1.04 | |
| DN1082_c4_g1_i11 | NA | CLIP-associating protein | RPRC005737 | 0.02 | -0.03 | 0.10 | 0.02 | -0.04 | -0.07 | |
| DN1082_c4_g1_i14 | NA | CLIP-associating protein | RPRC005737 | 0.12 | -1.16 | 1.01 | 0.32 | 0.28 | #NUM! | |
| DN1082_c4_g1_i21 | NA | CLIP-associating protein | RPRC005737 | 0.28 | -0.49 | 0.16 | -0.21 | 0.03 | 0.10 | |
| DN1082_c4_g1_i22 | NA | CLIP-associating protein | RPRC005737 | -0.38 | 0.45 | -0.26 | 0.23 | 0.27 | -0.61 | |
| DN1082_c4_g1_i27 | NA | CLIP-associating protein | RPRC005737 | 0.61 | -4.67 | 2.02 | -2.77 | -2.15 | #NUM! | |
| DN1082_c4_g1_i28 | NA | CLIP-associating protein | RPRC005737 | -0.18 | 0.71 | 0.97 | -0.55 | -0.26 | #NUM! | |
| DN1082_c4_g1_i31 | NA | CLIP-associating protein | RPRC005737 | 0.63 | 1.50 | -0.33 | -1.30 | -1.25 | #NUM! | |
| DN1082_c4_g1_i32 | NA | CLIP-associating protein | RPRC005737 | -0.85 | 1.04 | 0.55 | 0.21 | -0.37 | #NUM! | |
| DN1082_c4_g1_i33 | NA | CLIP-associating protein | RPRC005737 | -0.20 | -1.24 | 0.67 | 0.51 | 0.36 | -1.30 | |
| DN1082_c4_g1_i34 | NA | CLIP-associating protein | RPRC005737 | -1.81 | 0.70 | -0.61 | 1.02 | -0.60 | -0.42 | |
| DN1082_c4_g1_i35 | NA | CLIP-associating protein | RPRC005737 | 0.24 | 0.11 | -0.41 | -0.20 | -0.26 | 0.35 | |
| DN1082_c4_g1_i36 | NA | CLIP-associating protein | RPRC005737 | 0.51 | -0.34 | 0.87 | -0.18 | -0.06 | -3.12 | |
| DN1082_c4_g1_i37 | NA | CLIP-associating protein | RPRC005737 | -1.03 | -0.03 | -0.52 | 0.60 | 0.19 | 0.23 | |
| DN1082_c4_g1_i38 | NA | CLIP-associating protein | RPRC005737 | -1.26 | 0.51 | -0.87 | 0.41 | -0.75 | 0.75 | |
| DN1082_c4_g1_i39 | NA | CLIP-associating protein | RPRC005737 | 0.92 | -0.11 | 0.80 | -1.14 | 0.00 | #NUM! | |
| DN1082_c4_g1_i4 | NA | CLIP-associating protein | RPRC005737 | 0.34 | -1.86 | 1.39 | -0.48 | 0.16 | #NUM! | |
| DN1082_c4_g1_i5 | NA | CLIP-associating protein | RPRC005737 | 0.60 | 0.32 | 0.55 | -0.43 | 0.03 | #NUM! | |
| DN1082_c4_g1_i7 | NA | CLIP-associating protein | RPRC005737 | -0.37 | -0.22 | 0.16 | 0.38 | 0.63 | -1.31 | |
| DN11689_c0_g1_i11 | NA | Chitinase | RPRC011446 | 0.15 | -0.19 | -0.12 | -0.16 | -0.17 | 0.39 | |
| DN11689_c0_g1_i14 | NA | Chitinase | RPRC011446 | 0.04 | 0.22 | -0.62 | -0.25 | -0.07 | 0.45 | |
| DN11689_c0_g1_i16 | NA | Chitinase | RPRC011446 | 0.51 | -1.45 | 0.05 | -0.67 | -0.58 | 0.91 | |
| DN11689_c0_g1_i24 | NA | Chitinase | RPRC011446 | 0.10 | -0.25 | -0.35 | -0.10 | -0.02 | 0.48 | |
| DN11689_c0_g1_i34 | NA | Chitinase | RPRC011446 | 0.55 | -0.49 | -0.05 | -0.57 | -0.03 | 0.27 | |
| DN11689_c0_g1_i38 | NA | Chitinase | RPRC011446 | 0.90 | 0.16 | -0.36 | -1.32 | -1.41 | 0.55 | |
| DN11689_c0_g1_i4 | NA | Chitinase | RPRC011446 | 1.03 | -2.48 | 0.26 | -2.70 | 0.18 | 0.37 | |
| DN11689_c0_g1_i5 | NA | Chitinase | RPRC011446 | 1.31 | -2.75 | 0.24 | -3.10 | -0.44 | 0.43 | |
| DN11689_c0_g1_i8 | NA | Chitinase | RPRC011446 | 1.08 | -2.50 | 0.43 | -2.98 | 0.27 | 0.04 | |
| DN1204_c1_g2_i11 | NA | A-kinase anchor protein 1 | RPRC011024 | 1.28 | -3.26 | 0.33 | -3.09 | 0.17 | -0.06 | |
| DN1204_c1_g2_i12 | NA | A-kinase anchor protein 1 | RPRC011024 | 0.29 | -3.08 | -0.44 | -1.63 | 1.39 | -0.03 | |
| DN1204_c1_g2_i14 | NA | A-kinase anchor protein 1 | RPRC011024 | 0.89 | -2.40 | 0.38 | -6.51 | 0.62 | 0.15 | |
| DN1204_c1_g2_i15 | NA | A-kinase anchor protein 1 | RPRC011024 | 1.19 | -4.42 | 0.32 | -3.33 | 0.16 | 0.26 | |
| DN1204_c1_g2_i16 | NA | A-kinase anchor protein 1 | RPRC011024 | 1.13 | -2.03 | 0.49 | -2.78 | -0.56 | 0.42 | |
| DN1204_c1_g2_i19 | NA | A-kinase anchor protein 1 | RPRC011024 | 1.50 | -4.52 | 0.42 | -3.43 | -0.55 | 0.03 | |
| DN1204_c1_g2_i2 | NA | A-kinase anchor protein 1 | RPRC011024 | 0.48 | -0.05 | 0.06 | 0.13 | -0.13 | -0.80 | |
| DN1204_c1_g2_i20 | NA | A-kinase anchor protein 1 | RPRC011024 | 0.48 | -0.04 | 0.03 | 0.19 | -0.08 | -0.93 | |
| DN1204_c1_g2_i3 | NA | A-kinase anchor protein 1 | RPRC011024 | 0.04 | -0.14 | 0.44 | -0.15 | 0.00 | -0.33 | |
| DN1204_c1_g2_i5 | NA | A-kinase anchor protein 1 | RPRC011024 | 0.15 | 0.20 | -1.85 | -0.18 | -0.76 | 0.99 | |
| DN1204_c1_g2_i6 | NA | A-kinase anchor protein 1 | RPRC011024 | -0.07 | -0.13 | 0.81 | 0.10 | -0.06 | -1.53 | |
| DN1204_c1_g2_i8 | NA | A-kinase anchor protein 1 | RPRC011024 | 0.04 | -0.53 | 0.90 | -0.06 | 0.29 | -2.04 | |
| DN13091_c0_g1_i11 | NA | Chitotriosidase-1 | RPRC011446 | -0.11 | 0.32 | -0.43 | 0.48 | 0.27 | -1.07 | |
| DN13091_c0_g1_i14 | NA | Chitotriosidase-1 | RPRC011446 | 0.17 | -0.63 | 0.03 | -0.09 | -0.08 | 0.40 | |
| DN13091_c0_g1_i2 | NA | Chitotriosidase-1 | RPRC011446 | -0.39 | -0.56 | 0.16 | -0.32 | 0.58 | 0.20 | |
| DN1470_c2_g1_i1 | NA | eiger | RPRC011737 | -0.27 | -0.08 | -1.01 | 0.45 | -0.04 | 0.48 | |
| DN1470_c2_g1_i2 | NA | eiger | RPRC011737 | -0.14 | -0.12 | -0.75 | 0.01 | 0.15 | 0.55 | |
| DN2418_c0_g1_i10 | NA | Mariner | RPRC000586 | -0.86 | 0.60 | -1.35 | 0.86 | 0.41 | -1.32 | |
| DN2418_c0_g1_i12 | NA | Mariner | RPRC000586 | -1.12 | 0.23 | -0.52 | 1.04 | 0.17 | -1.04 | |
| DN2418_c0_g1_i17 | NA | Mariner | RPRC000586 | -1.35 | 1.14 | -1.26 | 0.07 | -0.45 | 0.27 | |
| DN2418_c0_g1_i5 | NA | Mariner | RPRC000586 | 0.29 | -1.79 | 0.24 | 1.15 | -0.27 | -1.98 | |
| DN2418_c0_g1_i8 | NA | Mariner | RPRC000586 | 0.08 | -0.33 | 0.58 | -0.32 | -0.23 | 0.00 | |
| DN2418_c0_g1_i9 | NA | Mariner | RPRC000586 | 0.19 | -0.68 | -0.25 | -0.14 | 0.11 | 0.50 | |
| DN2846_c0_g1_i10 | NA | Titin | RPRC017886 | 0.20 | -0.13 | 0.49 | 0.23 | -0.14 | -1.14 | |
| DN2846_c0_g1_i12 | NA | Titin | RPRC017887 | 0.49 | -0.01 | -0.26 | -0.16 | 0.29 | -0.61 | |
| DN2846_c0_g1_i14 | NA | Titin | RPRC017886 | 0.23 | -0.99 | 0.58 | -0.39 | 0.46 | -0.53 | |
| DN2846_c0_g1_i18 | NA | Titin | RPRC017886 | -0.05 | -0.47 | 0.72 | -0.33 | 0.16 | -0.41 | |
| DN2846_c0_g1_i6 | NA | Titin | RPRC017887 | 1.22 | -0.94 | 0.05 | 0.73 | -1.49 | -3.45 | |
| DN2846_c0_g1_i7 | NA | Titin | RPRC017886 | -0.42 | -0.75 | 0.27 | 0.86 | -0.24 | -0.35 | |
| DN3262_c0_g1_i1 | NA | Serine protease inhibitor 88Ea/rpSPI10a | RPRC002795 | -0.67 | -0.19 | -6.54 | 1.13 | -0.14 | 0.46 | |
| DN3262_c0_g1_i3 | NA | Serine protease inhibitor 88Ea/ rpSPI10a | RPRC002795 | -0.14 | 0.43 | -0.45 | 0.11 | 0.01 | -0.11 | |
| DN3262_c0_g1_i5 | NA | Serine protease inhibitor 88Ea/ rpSPI10a | RPRC002795 | -1.06 | 0.58 | -0.91 | -1.41 | 0.68 | 0.60 | |
| DN4045_c0_g1_i1 | NA | Titin | RPRC017887 | 0.95 | -2.59 | -0.06 | -2.57 | 0.20 | 0.70 | |
| DN4045_c0_g1_i12 | NA | Titin | RPRC017887 | 0.76 | -5.31 | 0.13 | -2.63 | -2.06 | 1.48 | |
| DN4045_c0_g1_i15 | NA | Titin | RPRC017887 | 1.04 | -2.47 | 0.08 | -2.31 | -0.02 | 0.60 | |
| DN4045_c0_g1_i19 | NA | Titin | RPRC017887 | -0.30 | 0.44 | -0.10 | 0.04 | -0.42 | 0.17 | |
| DN4045_c0_g1_i2 | NA | Titin | RPRC017887 | -0.73 | 0.91 | -0.31 | 0.44 | -1.43 | -0.03 | |
| DN4045_c0_g1_i21 | NA | Titin | RPRC017887 | -0.25 | 0.47 | -0.07 | 0.08 | -0.31 | -0.06 | |
| DN4045_c0_g1_i22 | NA | Titin | RPRC017887 | -1.07 | 1.05 | -0.11 | -0.12 | -2.22 | 0.48 | |
| DN4045_c0_g1_i23 | NA | Titin | RPRC017887 | 0.42 | -0.64 | 1.05 | 0.55 | -1.26 | -3.77 | |
| DN4045_c0_g1_i26 | NA | Titin | RPRC017887 | -1.23 | 0.38 | 0.12 | 0.32 | -0.24 | 0.12 | |
| DN4045_c0_g1_i30 | NA | Titin | RPRC017887 | 0.40 | 0.58 | 0.31 | -0.36 | -0.10 | -2.10 | |
| DN4045_c0_g1_i31 | NA | Titin | RPRC017887 | 0.42 | -0.50 | -3.28 | 0.64 | -0.44 | 0.65 | |
| DN4045_c0_g1_i35 | NA | Titin | RPRC017887 | -0.16 | 0.12 | -0.33 | 0.14 | -0.10 | 0.26 | |
| DN4045_c0_g1_i4 | NA | Titin | RPRC017887 | -0.46 | 0.12 | -0.58 | 0.73 | -0.12 | -0.08 | |
| DN4045_c0_g1_i8 | NA | Titin | RPRC017887 | -0.61 | 0.35 | -0.48 | 0.42 | -0.09 | 0.10 | |
| DN4045_c0_g1_i9 | NA | Titin | RPRC017887 | -0.36 | -0.14 | -0.10 | 0.52 | -0.06 | -0.02 | |
| DN4113_c0_g1_i10 | NA | Sequestosome | NA | 0.08 | -5.42 | 2.07 | -4.05 | -0.82 | -3.65 | |
| DN4113_c0_g1_i12 | NA | Sequestosome | NA | 0.91 | -1.45 | -2.57 | 0.18 | -0.39 | 0.76 | |
| DN4113_c0_g1_i14 | NA | Sequestosome | NA | -0.04 | -0.56 | 0.86 | -0.07 | 0.10 | -1.00 | |
| DN4113_c0_g1_i15 | NA | Sequestosome | NA | 0.61 | -0.28 | -0.11 | -0.48 | -0.13 | 0.13 | |
| DN4113_c0_g1_i16 | NA | Sequestosome | NA | 0.83 | -0.45 | 0.68 | -0.09 | -0.44 | -2.21 | |
| DN4113_c0_g1_i18 | NA | Sequestosome | NA | 0.08 | 0.59 | 0.24 | -0.03 | -0.56 | -0.72 | |
| DN4113_c0_g1_i19 | NA | Sequestosome | NA | 0.73 | 0.02 | -0.41 | -0.44 | 0.04 | -0.31 | |
| DN4113_c0_g1_i2 | NA | Sequestosome | NA | -0.72 | #NUM! | 0.24 | -1.38 | 0.91 | 0.96 | |
| DN4113_c0_g1_i20 | NA | Sequestosome | NA | 0.84 | -0.63 | -0.24 | -0.64 | -0.39 | 0.40 | |
| DN4113_c0_g1_i21 | NA | Sequestosome | NA | 1.09 | -1.47 | 0.08 | 0.42 | -0.53 | -1.24 | |
| DN4113_c0_g1_i3 | NA | Sequestosome | NA | 0.86 | -1.58 | -0.50 | -0.82 | -0.88 | 1.03 | |
| DN4113_c0_g1_i4 | NA | Sequestosome | NA | 0.14 | -0.01 | 0.12 | 0.12 | 0.04 | -0.50 | |
| DN4113_c0_g1_i5 | NA | Sequestosome | NA | 0.15 | 0.01 | 0.11 | 0.20 | 0.09 | -0.73 | |
| DN4113_c0_g1_i6 | NA | Sequestosome | NA | 0.61 | -1.07 | -0.45 | -0.78 | -0.51 | 0.99 | |
| DN4113_c0_g1_i8 | NA | Sequestosome | NA | 0.63 | -1.01 | -0.38 | -0.65 | -0.39 | 0.84 | |
| DN4113_c0_g1_i9 | NA | Sequestosome | NA | 0.14 | -0.25 | -0.10 | -0.16 | -0.13 | 0.40 | |
| DN13642_c0_g1_i1 | Others | HoxCaudal | RPRC000239 | -0.02 | 0.15 | -0.31 | 0.05 | -0.02 | 0.11 | |
| DN310_c1_g1_i1 | Others | Nubbin | RPRC009416 | 0.08 | 0.11 | 0.34 | -0.09 | 0.02 | -0.62 | |
| DN310_c1_g1_i3 | Others | Nubbin | RPRC009416 | -0.39 | 0.30 | -0.50 | 0.25 | 0.08 | 0.07 | |
| DN33_c0_g2_i1 | Others | Dsp1 | RPRC006379 | 0.82 | -1.66 | -0.24 | -1.57 | -0.62 | 1.06 | |
| DN33_c0_g2_i10 | Others | Dsp1 | RPRC006379 | 1.28 | -1.20 | -0.56 | -1.15 | -2.48 | 0.87 | |
| DN33_c0_g2_i11 | Others | Dsp1 | RPRC006379 | -0.08 | -1.33 | 0.33 | -0.97 | -0.03 | 0.93 | |
| DN33_c0_g2_i13 | Others | Dsp1 | RPRC006379 | 0.87 | -1.63 | -0.15 | -1.46 | -0.51 | 0.92 | |
| DN33_c0_g2_i2 | Others | Dsp1 | RPRC006379 | 0.13 | 0.65 | -0.90 | 0.42 | -0.37 | -0.55 | |
| DN33_c0_g2_i4 | Others | Dsp1 | RPRC006379 | 1.27 | -4.88 | -1.06 | -3.33 | -1.16 | 1.34 | |
| DN33_c0_g2_i5 | Others | Dsp1 | RPRC006379 | 0.71 | -2.70 | 0.68 | -2.89 | -0.21 | 0.69 | |
| DN33_c0_g2_i7 | Others | Dsp1 | RPRC006379 | -0.43 | 0.25 | -0.45 | -0.06 | -0.06 | 0.51 | |
| DN33_c0_g2_i8 | Others | Dsp1 | RPRC006379 | -6.29 | 0.89 | -0.76 | 0.47 | 0.46 | -0.35 | |
| DN3515_c1_g1_i1 | Others | dUSP36 | RPRC001267 | -1.64 | 0.87 | -1.17 | -1.47 | -0.04 | 1.05 | |
| DN5777_c0_g1_i1 | Others | SkpA | RPRC011803 | 0.26 | -0.36 | 0.49 | 0.22 | -0.35 | -0.60 | |
| DN6450_c0_g1_i1 | Others | Trabid | RPRC005572 | -1.39 | 0.49 | -0.85 | 0.79 | 0.01 | -0.10 | |
| DN6450_c0_g1_i10 | Others | Trabid | RPRC005572 | -0.67 | 0.18 | -0.66 | 0.64 | -0.06 | 0.12 | |
| DN6450_c0_g1_i11 | Others | Trabid | RPRC005572 | 0.16 | 0.04 | 0.26 | -0.16 | -0.01 | -0.38 | |
| DN6450_c0_g1_i12 | Others | Trabid | RPRC005572 | 0.77 | -0.64 | -0.14 | -1.00 | 0.59 | -0.45 | |
| DN6450_c0_g1_i2 | Others | Trabid | RPRC005572 | 0.03 | 0.16 | 0.30 | 0.03 | -0.09 | -0.58 | |
| DN6450_c0_g1_i3 | Others | Trabid | RPRC005572 | 0.27 | -0.05 | 0.20 | 0.04 | -0.23 | -0.31 | |
| DN6450_c0_g1_i4 | Others | Trabid | RPRC005572 | 0.56 | 0.08 | -0.01 | -0.10 | -0.48 | -0.28 | |
| DN6450_c0_g1_i5 | Others | Trabid | RPRC005572 | -0.08 | -0.47 | 0.63 | -0.16 | 0.70 | -1.93 | |
| DN6450_c0_g1_i6 | Others | Trabid | RPRC005572 | 0.24 | 0.14 | -0.05 | 0.44 | -1.05 | -0.13 | |
| DN6450_c0_g1_i7 | Others | Trabid | RPRC005572 | 0.16 | -0.21 | 0.26 | -0.03 | -0.02 | -0.22 | |
| DN6450_c0_g1_i8 | Others | Trabid | RPRC005572 | -0.01 | 0.10 | -0.13 | 0.19 | 0.25 | -0.53 | |
| DN6450_c0_g1_i9 | Others | Trabid | RPRC005572 | -0.28 | -1.08 | 1.44 | -0.08 | -0.57 | -1.47 | |
| DN6670_c0_g1_i2 | Others | Zfh1 | RPRC009393 | -0.16 | 0.07 | -0.53 | 0.20 | -0.32 | 0.50 | |
| DN6670_c0_g1_i3 | Others | Zfh1 | RPRC009393 | -0.13 | 0.14 | -0.78 | 0.43 | -0.39 | 0.36 | |
| DN6670_c0_g1_i4 | Others | Zfh1 | RPRC009393 | -0.50 | 0.25 | -0.92 | 0.40 | -0.17 | 0.45 | |
| DN6798_c0_g2_i1 | Others | POSH | RPRC004576 | 0.26 | -0.11 | 0.14 | 0.03 | 0.15 | -0.65 | |
| DN13934_c0_g1_i1 | Peroxidases | Peroxidase-like2 | RPRC012732 | -0.13 | 0.13 | -0.32 | 0.15 | 0.17 | -0.06 | |
| DN19960_c0_g2_i1 | Peroxidases | Peroxidase-like5 | RPRC012999 | 0.14 | -0.06 | 0.02 | -0.01 | -0.05 | -0.06 | |
| DN21538_c0_g1_i1 | Peroxidases | Thioredoxin peroxidases | RPRC012922 | 0.71 | -1.78 | -0.14 | -1.28 | -0.22 | 0.92 | |
| DN21538_c0_g1_i3 | Peroxidases | Thioredoxin peroxidases | RPRC012922 | 1.82 | -3.61 | -0.42 | -2.64 | -3.80 | 0.49 | |
| DN21538_c0_g1_i4 | Peroxidases | Thioredoxin peroxidases | RPRC012922 | 0.90 | -2.28 | -0.24 | -1.13 | -0.31 | 0.87 | |
| DN21538_c0_g1_i5 | Peroxidases | Thioredoxin peroxidases | RPRC012922 | 1.65 | -0.59 | 0.39 | -6.24 | -0.85 | -1.62 | |
| DN21538_c0_g1_i6 | Peroxidases | Thioredoxin peroxidases | RPRC012922 | 0.20 | -2.86 | -0.20 | -0.75 | 0.27 | 1.03 | |
| DN21538_c0_g1_i7 | Peroxidases | Thioredoxin peroxidases | RPRC012922 | 0.65 | -1.73 | -0.03 | -0.99 | -0.06 | 0.75 | |
| DN2263_c0_g3_i2 | Peroxidases | Haem (Heme) peroxidase | RPRC000982 | -0.10 | -0.63 | -0.39 | 0.04 | 0.21 | 0.56 | |
| DN2263_c0_g3_i7 | Peroxidases | Haem (Heme) peroxidase | RPRC000982 | -0.07 | -0.68 | -0.43 | 0.19 | 0.34 | 0.36 | |
| DN2455_c0_g1_i1 | Peroxidases | Glutathione peroxidase- CysGPx | RPRC015008 | 0.10 | -0.01 | 0.11 | 0.01 | -0.02 | -0.20 | |
| DN2455_c0_g1_i15 | Peroxidases | Glutathione peroxidase- CysGPx | RPRC015008 | 0.59 | 0.38 | 0.38 | -0.53 | -0.65 | -0.82 | |
| DN2455_c0_g1_i16 | Peroxidases | Glutathione peroxidase- CysGPx | RPRC015008 | 0.12 | -0.05 | 0.15 | 0.12 | 0.03 | -0.44 | |
| DN2455_c0_g1_i22 | Peroxidases | Glutathione peroxidase- CysGPx | RPRC015008 | 0.00 | 0.11 | -0.14 | 0.09 | 0.13 | -0.23 | |
| DN2455_c0_g1_i27 | Peroxidases | Glutathione peroxidase- CysGPx | RPRC015008 | -0.02 | 0.10 | -0.17 | -0.14 | -0.13 | 0.30 | |
| DN2455_c0_g1_i30 | Peroxidases | Glutathione peroxidase- CysGPx | RPRC015008 | -0.04 | 0.11 | -0.28 | 0.00 | -0.04 | 0.20 | |
| DN2455_c0_g1_i32 | Peroxidases | Glutathione peroxidase- CysGPx | RPRC015008 | 0.39 | -0.21 | 0.69 | -0.11 | -0.26 | -1.17 | |
| DN2695_c0_g1_i1 | Peroxidases | Haem (Heme) peroxidase | RPRC006648 | -0.74 | 0.40 | -0.55 | 0.78 | -0.33 | -0.19 | |
| DN2695_c0_g1_i2 | Peroxidases | Haem (Heme) peroxidase | RPRC006648 | 0.30 | -0.87 | 0.30 | -0.13 | -0.04 | 0.15 | |
| DN2695_c0_g1_i4 | Peroxidases | Haem (Heme) peroxidase | RPRC006648 | -0.96 | 0.81 | -1.20 | 0.82 | -0.49 | -0.29 | |
| DN2695_c0_g1_i5 | Peroxidases | Haem (Heme) peroxidase | RPRC006648 | -0.60 | 0.42 | -0.92 | -0.20 | -0.49 | 0.92 | |
| DN2695_c0_g1_i6 | Peroxidases | Haem (Heme) peroxidase | RPRC006648 | -0.53 | 0.53 | -0.81 | -0.16 | -0.40 | 0.71 | |
| DN2695_c0_g1_i9 | Peroxidases | Haem (Heme) peroxidase | RPRC006648 | -0.22 | -0.30 | -1.07 | 1.33 | -0.13 | -1.21 | |
| DN4914_c0_g1_i2 | Peroxidases | Thioredoxin peroxidases | RPRC007595 | -1.48 | 0.05 | 0.08 | 0.44 | -0.04 | 0.28 | |
| DN4914_c0_g1_i3 | Peroxidases | Thioredoxin peroxidases | RPRC007595 | -1.35 | 0.44 | -0.68 | 0.06 | -0.63 | 0.95 | |
| DN4914_c0_g1_i5 | Peroxidases | Thioredoxin peroxidases | RPRC007595 | -0.10 | 0.08 | -0.04 | 0.07 | -0.05 | 0.03 | |
| DN5350_c0_g1_i3 | Peroxidases | Haem (Heme) peroxidase | RPRC005268 | 0.14 | -0.34 | 0.94 | 0.41 | -0.34 | -3.87 | |
| DN5350_c0_g1_i8 | Peroxidases | Haem (Heme) peroxidase | RPRC005268 | -0.19 | -0.27 | -0.35 | 0.03 | 0.08 | 0.52 | |
| DN5350_c0_g3_i1 | Peroxidases | Haem (Heme) peroxidase | RPRC013394 | 0.21 | -1.15 | 0.74 | -1.91 | 0.44 | 0.14 | |
| DN604_c0_g1_i10 | Peroxidases | Peroxiredoxin 6 | RPRC015401 | 0.12 | -0.63 | 0.37 | -1.91 | 1.06 | -0.68 | |
| DN604_c0_g1_i12 | Peroxidases | Peroxiredoxin 6 | RPRC015401 | 0.51 | -0.40 | 1.15 | -0.57 | -0.85 | -1.42 | |
| DN604_c0_g1_i3 | Peroxidases | Peroxiredoxin 6 | RPRC015401 | 0.34 | -0.18 | 0.91 | -0.39 | -0.08 | -1.89 | |
| DN604_c0_g1_i7 | Peroxidases | Peroxiredoxin 6 | RPRC015401 | -0.09 | -0.07 | 0.01 | 0.22 | 0.26 | -0.44 | |
| DN604_c0_g1_i9 | Peroxidases | Peroxiredoxin 6 | RPRC015401 | -0.05 | -0.03 | 0.03 | 0.07 | -0.20 | 0.16 | |
| DN7065_c0_g1_i1 | Peroxidases | Thioredoxin peroxidases | RPRC011126 | 0.76 | -0.61 | 1.10 | 0.14 | -1.28 | #NUM! | |
| DN7065_c0_g1_i2 | Peroxidases | Thioredoxin peroxidases | RPRC011126 | 0.12 | -0.02 | 0.14 | 0.18 | 0.23 | -0.94 | |
| DN7065_c0_g1_i4 | Peroxidases | Thioredoxin peroxidases | RPRC011126 | 0.37 | -0.41 | 0.39 | -0.35 | 0.39 | -0.85 | |
| DN7150_c0_g1_i1 | Peroxidases | Peroxidase-like1 | RPRC009148 | -0.03 | 0.00 | 0.19 | -0.03 | -0.03 | -0.12 | |
| DN7150_c0_g1_i2 | Peroxidases | Peroxidase-like1 | RPRC009148 | -0.31 | 0.26 | -0.22 | 0.11 | -0.09 | 0.16 | |
| DN7150_c0_g1_i3 | Peroxidases | Peroxidase-like1 | N | 0.38 | 0.01 | -0.06 | -0.67 | -0.17 | 0.28 | |
| DN7688_c0_g1_i1 | Peroxidases | Peroxiredoxin 2540 | RPRC015401 | 0.04 | 0.00 | 0.25 | 0.05 | 0.07 | -0.51 | |
| DN7688_c0_g1_i2 | Peroxidases | Peroxiredoxin 2540 | RPRC015388 | 0.17 | -0.12 | 0.27 | -0.16 | 0.07 | -0.31 | |
| DN7810_c0_g4_i1 | Peroxidases | Peroxiredoxin 2540 | RPRC000537 | 0.18 | -0.09 | 0.25 | -0.09 | 0.12 | -0.49 | |
| DN915_c1_g3_i1 | Peroxidases | Peroxidase-like6 | RPRC005064 | 0.00 | 0.02 | 0.33 | 0.04 | 0.04 | -0.56 | |
| DN10315_c0_g1_i1 | PRR | CSP Chymotrypsinogen/rpSP1 | RPRC014822 | 0.27 | -0.07 | 0.51 | -0.51 | 0.23 | -0.89 | |
| DN1121_c0_g1_i16 | PRR | Scavenger Protein croquemort | RPRC015150 | -0.17 | 0.23 | -0.03 | 0.03 | 0.00 | -0.09 | |
| DN1121_c0_g1_i17 | PRR | Scavenger Protein croquemort | RPRC015150 | -0.35 | 0.13 | 0.00 | 0.20 | 0.21 | -0.28 | |
| DN1121_c0_g1_i2 | PRR | Scavenger Protein croquemort | RPRC015150 | 0.07 | 0.41 | -0.06 | 0.01 | -0.17 | -0.38 | |
| DN1121_c0_g1_i20 | PRR | Scavenger Protein croquemort | RPRC015150 | 0.06 | -0.50 | -0.35 | -0.39 | 0.21 | 0.63 | |
| DN1121_c0_g1_i21 | PRR | Scavenger Protein croquemort | RPRC015150 | 0.06 | -0.47 | -1.22 | 0.36 | 0.00 | 0.61 | |
| DN1121_c0_g1_i6 | PRR | Scavenger Protein croquemort | RPRC015150 | -2.39 | -0.54 | -1.60 | -0.03 | 1.50 | -0.01 | |
| DN1121_c0_g1_i9 | PRR | Scavenger Protein croquemort | RPRC015150 | 0.40 | -0.33 | -1.65 | -0.41 | -0.36 | 1.02 | |
| DN11216_c0_g1_i1 | PRR | Scavenger Receptor Class B | RPRC012472 | -0.69 | -0.12 | -0.29 | -0.55 | 0.66 | 0.47 | |
| DN11216_c0_g1_i12 | PRR | Scavenger Receptor Class B | RPRC012472 | 1.07 | -2.13 | -0.05 | 0.24 | -1.73 | 0.29 | |
| DN11216_c0_g1_i21 | PRR | Scavenger Receptor Class B | RPRC012472 | -0.08 | -0.08 | 0.70 | #NUM! | 0.54 | 0.04 | |
| DN11216_c0_g1_i23 | PRR | Scavenger Receptor Class B | RPRC012472 | 0.20 | -0.21 | -0.38 | -0.74 | 0.07 | 0.65 | |
| DN11216_c0_g1_i9 | PRR | Scavenger Receptor Class B | RPRC012472 | -0.58 | 0.59 | -0.99 | 0.35 | -0.54 | 0.45 | |
| DN11528_c0_g1_i10 | PRR | Phenoloxidase-activating factor 2/ rpSP5 | RPRC005405 | -0.71 | 0.65 | -0.99 | 0.49 | -0.43 | 0.23 | |
| DN11528_c0_g1_i11 | PRR | Phenoloxidase-activating factor 2/ rpSP5 | RPRC005405 | -0.33 | 0.30 | -1.50 | 0.82 | -2.49 | 0.74 | |
| DN11528_c0_g1_i12 | PRR | Phenoloxidase-activating factor 2/ rpSP5 | RPRC005405 | -1.45 | 1.57 | -0.72 | 0.08 | -3.69 | -0.09 | |
| DN11528_c0_g1_i14 | PRR | Phenoloxidase-activating factor 2/ rpSP5 | RPRC005405 | -0.76 | 1.35 | -2.28 | 0.57 | -1.08 | -0.53 | |
| DN11528_c0_g1_i17 | PRR | Phenoloxidase-activating factor 2/ rpSP5 | RPRC005405 | 0.20 | -1.85 | 0.19 | 0.23 | 0.84 | -1.12 | |
| DN11528_c0_g1_i19 | PRR | Phenoloxidase-activating factor 2/ rpSP5 | RPRC005405 | 0.06 | -0.25 | -0.45 | -0.30 | -0.15 | 0.74 | |
| DN11528_c0_g1_i22 | PRR | Phenoloxidase-activating factor 2/ rpSP5 | RPRC005405 | 0.36 | -1.98 | 0.15 | -0.35 | -0.25 | 0.79 | |
| DN11528_c0_g1_i6 | PRR | Phenoloxidase-activating factor 2/ rpSP5 | RPRC005405 | 0.20 | -1.59 | -0.17 | -0.17 | -0.08 | 0.85 | |
| DN11528_c0_g1_i9 | PRR | Phenoloxidase-activating factor 2/ rpSP5 | RPRC005405 | 0.21 | 0.37 | -0.22 | -0.50 | -0.49 | 0.35 | |
| DN12159_c0_g2_i1 | PRR | Scavenger Receptor Class B | RPRC010586 | -0.14 | 0.78 | -1.80 | 0.41 | -0.29 | -0.10 | |
| DN12201_c0_g1_i1 | PRR | Venom Clip-domain Serine Protease/rpSP6 | RPRC005759 | 0.27 | 0.28 | 0.84 | -1.00 | -0.47 | -0.82 | |
| DN12201_c0_g1_i2 | PRR | Venom Clip-domain Serine Protease/rp/rpSP6 | RPRC005759 | 0.05 | 0.07 | 0.22 | 0.17 | 0.17 | -1.01 | |
| DN14254_c0_g2_i2 | PRR | C-Type-lectin | RPRC006426 | 0.06 | -0.08 | 0.61 | -0.26 | -0.15 | -0.41 | |
| DN14254_c0_g2_i4 | PRR | C-Type-lectin | RPRC006426 | 0.27 | -0.31 | -0.20 | -0.22 | -0.13 | 0.43 | |
| DN1438_c0_g1_i1 | PRR | PGRP | RPRC014061 | 0.01 | -0.11 | -0.26 | 0.10 | -0.05 | 0.25 | |
| DN1438_c0_g1_i10 | PRR | PGRP | RPRC014061 | -0.18 | 0.04 | -0.55 | 0.16 | 0.12 | 0.27 | |
| DN1438_c0_g1_i11 | PRR | PGRP | RPRC014061 | -0.02 | -0.50 | -2.83 | 0.14 | 0.92 | 0.23 | |
| DN1438_c0_g1_i15 | PRR | PGRP | RPRC014061 | -0.21 | -0.25 | -0.70 | 0.42 | -0.11 | 0.50 | |
| DN1438_c0_g1_i2 | PRR | PGRP | RPRC014061 | 0.59 | 0.06 | 0.10 | 0.28 | -0.51 | -1.11 | |
| DN14442_c0_g1_i7 | PRR | Proclotting enzyme/rpSP8 | RPRC009383 | 0.06 | -0.15 | -0.15 | 0.32 | 0.10 | -0.26 | |
| DN14442_c0_g1_i8 | PRR | Proclotting enzyme/rpSP8 | RPRC009383 | 0.09 | -0.11 | 0.01 | 0.24 | 0.02 | -0.31 | |
| DN1521_c2_g1_i20 | PRR | Thio-Ester Containing Proteins | RPRC006447 | -0.29 | 0.07 | -0.60 | -0.03 | -0.04 | 0.60 | |
| DN1521_c2_g1_i21 | PRR | Thio-Ester Containing Proteins | RPRC006447 | 0.02 | -0.22 | 0.64 | -0.11 | 0.21 | -1.04 | |
| DN1521_c2_g1_i23 | PRR | Thio-Ester Containing Proteins | RPRC006447 | 0.02 | -0.22 | 0.61 | -0.05 | 0.25 | -1.15 | |
| DN1521_c2_g1_i35 | PRR | Thio-Ester Containing Proteins | RPRC006447 | -0.28 | 0.10 | -0.03 | -0.16 | -0.55 | 0.63 | |
| DN1521_c2_g1_i40 | PRR | Thio-Ester Containing Proteins | RPRC006447 | -0.18 | 0.36 | -0.35 | -0.18 | -0.18 | 0.36 | |
| DN1521_c2_g1_i41 | PRR | Thio-Ester Containing Proteins | RPRC006447 | 0.24 | -0.37 | 1.08 | -0.15 | -1.02 | -0.90 | |
| DN1532_c0_g1_i1 | PRR | Scavenger Receptor Class B | RPRC003038/ RPRC003040 | -0.04 | 0.19 | 0.03 | 0.14 | 0.03 | -0.43 | |
| DN1532_c0_g1_i11 | PRR | Scavenger Receptor Class B | RPRC003038/ RPRC003040 | -0.04 | 0.11 | -0.39 | 0.15 | 0.01 | 0.09 | |
| DN1532_c0_g1_i12 | PRR | Scavenger Receptor Class B | RPRC003038 /RPRC003040 | -0.80 | -0.47 | 1.30 | -0.47 | -0.29 | -0.53 | |
| DN1532_c0_g1_i13 | PRR | Scavenger Receptor Class B | RPRC003038 /RPRC003040 | -0.09 | 0.20 | -0.11 | -0.06 | 0.06 | -0.02 | |
| DN1532_c0_g1_i2 | PRR | Scavenger Receptor Class B | RPRC003038 /RPRC003040 | 0.05 | -0.43 | 0.70 | -0.36 | 0.46 | -1.17 | |
| DN1532_c0_g1_i3 | PRR | Scavenger Receptor Class B | RPRC003038/ RPRC003040 | -1.30 | 0.59 | -0.54 | -0.78 | 0.64 | 0.34 | |
| DN1532_c0_g1_i4 | PRR | Scavenger Receptor Class B | RPRC003038/ RPRC003040 | -0.06 | 0.34 | -0.41 | 0.17 | 0.02 | -0.18 | |
| DN1532_c0_g1_i7 | PRR | Scavenger Receptor Class B | RPRC003038/ RPRC003040 | 0.28 | -0.25 | -0.12 | 0.47 | 0.00 | -0.63 | |
| DN1532_c0_g1_i8 | PRR | Scavenger Receptor Class B | RPRC003038/ RPRC003040 | 0.76 | -0.58 | 0.86 | 0.48 | -1.20 | #NUM! | |
| DN1532_c0_g1_i9 | PRR | Scavenger Receptor Class B | RPRC003038/ RPRC003040 | 0.52 | -0.18 | 0.26 | -0.13 | -0.01 | -0.81 | |
| DN15904_c0_g1_i1 | PRR | C-Type-lectin | RPRC007450 | 0.58 | -0.42 | 0.52 | -2.44 | 0.59 | -0.67 | |
| DN20211_c0_g1_i1 | PRR | Clip-domain Serine Protease | RPRC000913 | 0.97 | -1.77 | -0.23 | 0.51 | #NUM! | 0.56 | |
| DN2051_c4_g1_i1 | PRR | Sanake-CSP | RPRC000033 | 0.72 | -0.88 | 0.31 | 0.44 | -0.23 | -1.45 | |
| DN2051_c4_g1_i2 | PRR | Sanake-CSP | RPRC000033 | 0.11 | -0.02 | -0.10 | 0.00 | 0.03 | -0.02 | |
| DN2051_c4_g1_i3 | PRR | Sanake-CSP | RPRC000033 | 0.50 | -0.15 | -0.20 | 0.51 | -0.48 | -0.58 | |
| DN23039_c0_g1_i15 | PRR | Proclotting enzyme | RPRC011066 | -1.02 | 0.79 | -0.65 | 0.68 | -0.51 | -0.26 | |
| DN23039_c0_g1_i21 | PRR | Proclotting enzyme | RPRC011066 | -0.09 | -0.32 | -0.17 | 0.13 | 0.02 | 0.35 | |
| DN23039_c0_g1_i9 | PRR | Proclotting enzyme | RPRC011066 | -0.05 | -0.58 | -0.33 | 0.15 | 0.28 | 0.31 | |
| DN2304_c1_g1_i35 | PRR | Scavenger Receptor Class B | RPRC002755 | -1.72 | 0.18 | 1.12 | 0.31 | -0.53 | -1.14 | |
| DN2304_c1_g1_i38 | PRR | Scavenger Receptor Class B | RPRC002756 | -1.66 | 0.24 | 1.08 | 0.36 | -0.48 | -1.36 | |
| DN2304_c1_g1_i48 | PRR | Scavenger Receptor Class B | RPRC002757 | -1.14 | 0.73 | -0.85 | 0.49 | -0.21 | 0.08 | |
| DN2595_c0_g1_i1 | PRR | Protein croquemort | RPRC009308 | 0.00 | 0.20 | -0.22 | -0.03 | -0.11 | 0.12 | |
| DN2595_c0_g1_i2 | PRR | Protein croquemort | RPRC009306 | 0.34 | -0.05 | 0.67 | -0.61 | 0.25 | -1.61 | |
| DN2595_c0_g1_i3 | PRR | Protein croquemort | RPRC009306 | -0.23 | -0.94 | -0.02 | 1.21 | -0.56 | -0.61 | |
| DN2595_c0_g1_i5 | PRR | Protein croquemort | RPRC009308 | -0.25 | -0.72 | -0.64 | 0.75 | 0.23 | 0.07 | |
| DN2595_c0_g1_i7 | PRR | Protein croquemort | RPRC009306 | -0.63 | 0.37 | -0.89 | 0.56 | -0.07 | 0.14 | |
| DN2658_c0_g1_i11 | PRR | GNBP | RPRC003210 | 0.09 | -0.94 | -0.35 | -0.12 | 0.47 | 0.40 | |
| DN2658_c0_g1_i12 | PRR | GNBP | RPRC003210 | 0.01 | -0.46 | -0.77 | 0.00 | 0.35 | 0.49 | |
| DN2658_c0_g1_i16 | PRR | GNBP | RPRC003210 | -0.99 | 0.82 | -1.26 | 0.81 | -0.42 | -0.30 | |
| DN2658_c0_g1_i19 | PRR | GNBP | RPRC003210 | -1.59 | 1.00 | -0.54 | 0.96 | -0.73 | -1.22 | |
| DN2658_c0_g1_i6 | PRR | GNBP | RPRC003210 | 0.00 | -2.22 | 0.84 | -1.24 | -0.06 | 0.69 | |
| DN2658_c0_g1_i8 | PRR | GNBP | RPRC003210 | -0.56 | 0.06 | -0.74 | 0.63 | 0.30 | -0.15 | |
| DN297_c0_g2_i10 | PRR | Clip-domain Serine Protease-SNAKE/rpSP30 | NA | 0.37 | -0.30 | 0.17 | -0.55 | -0.01 | 0.14 | |
| DN297_c0_g2_i11 | PRR | Clip-domain Serine Protease-SNAKE/rpSP30 | NA | 0.49 | -0.16 | 0.82 | -1.38 | 0.21 | -1.40 | |
| DN297_c0_g2_i15 | PRR | Clip-domain Serine Protease-SNAKE/rpSP30 | NANA | 0.32 | 0.25 | 0.49 | -1.70 | -1.72 | 0.63 | |
| DN297_c0_g2_i21 | PRR | Clip-domain Serine Protease-SNAKE/rpSP30 | RPRC009729 | 0.42 | -0.89 | -0.21 | -1.03 | 0.10 | 0.76 | |
| DN297_c0_g2_i22 | PRR | Clip-domain Serine Protease-SNAKE/rpSP30 | NA | -1.44 | 1.20 | -1.42 | 0.68 | -0.84 | -0.33 | |
| DN297_c0_g2_i25 | PRR | Clip-domain Serine Protease-SNAKE/rpSP30 | NA | 0.14 | -0.03 | -0.45 | 0.15 | 0.14 | -0.03 | |
| DN297_c0_g2_i26 | PRR | Clip-domain Serine Protease-SNAKE/rpSP30 | NA | -0.90 | 0.73 | -1.19 | 0.78 | -0.29 | -0.25 | |
| DN297_c0_g2_i27 | PRR | Clip-domain Serine Protease-SNAKE/rpSP30 | NA | -4.02 | -0.97 | -3.93 | 1.10 | 1.62 | -2.89 | |
| DN297_c0_g2_i3 | PRR | Clip-domain Serine Protease-SNAKE/rpSP30 | RPRC009729 | -0.65 | 0.67 | -0.95 | 0.70 | -0.38 | -0.21 | |
| DN297_c0_g2_i30 | PRR | Clip-domain Serine Protease-SNAKE/rpSP30 | RPRC009729 | 0.04 | -0.85 | 0.41 | -0.32 | 0.33 | 0.05 | |
| DN297_c0_g2_i4 | PRR | Clip-domain Serine Protease-SNAKE/rpSP30 | RPRC009729 | -0.60 | -0.18 | -1.25 | 0.58 | 0.85 | -0.46 | |
| DN3249_c0_g1_i1 | PRR | Clip-domain Serine Protease/rp35 | RPRC014770 | 0.18 | 0.24 | -0.14 | 0.15 | -0.44 | -0.10 | |
| DN3249_c0_g1_i2 | PRR | Clip-domain Serine Protease/rp35 | RPRC014770 | -0.19 | 0.27 | -0.40 | 0.05 | -0.13 | 0.27 | |
| DN3249_c0_g1_i3 | PRR | Clip-domain Serine Protease/rp35 | RPRC014770 | 0.13 | -0.43 | 0.40 | -0.70 | 0.10 | 0.21 | |
| DN3249_c0_g1_i5 | PRR | Clip-domain Serine Protease/rp35 | RPRC014770 | -0.70 | -0.72 | -0.19 | 0.45 | 0.42 | 0.25 | |
| DN3249_c0_g1_i7 | PRR | Clip-domain Serine Protease/rp35 | RPRC014770 | -0.14 | 0.28 | -0.34 | 0.17 | -0.09 | 0.02 | |
| DN33_c1_g2_i10 | PRR | PGRP | RPRC007262/RPRC017796B | -2.35 | 0.71 | -0.54 | 0.65 | 0.61 | -1.38 | |
| DN33_c1_g2_i12 | PRR | PGRP | RPRC007262/RPRC017796B | -1.27 | 0.53 | -0.47 | 0.34 | 0.31 | -0.14 | |
| DN33_c1_g2_i15 | PRR | PGRP | RPRC007262/RPRC017796A | -0.62 | 0.30 | -0.19 | 0.20 | -0.27 | 0.33 | |
| DN33_c1_g2_i17 | PRR | PGRP | RPRC007262/RPRC017796C | 0.08 | 0.39 | -0.56 | 0.05 | -0.07 | -0.05 | |
| DN33_c1_g2_i19 | PRR | PGRP | RPRC007262/RPRC017796B | -0.38 | 0.09 | -0.32 | -0.11 | -0.08 | 0.58 | |
| DN33_c1_g2_i5 | PRR | PGRP | RPRC007262/RPRC017673 | -0.92 | 0.84 | -1.08 | 0.67 | -0.57 | -0.10 | |
| DN33_c1_g2_i7 | PRR | PGRP | RPRC007262/RPRC017796B | -0.92 | 0.87 | -1.07 | 0.77 | -0.52 | -0.38 | |
| DN33_c1_g2_i8 | PRR | PGRP | RPRC007262/RPRC017796B | -0.24 | 0.19 | -1.03 | 0.13 | -0.57 | 0.82 | |
| DN3340_c0_g1_i1 | PRR | Scavenger Receptor Class B | RPRC013775 | -0.50 | 0.45 | -0.62 | 0.13 | -0.40 | 0.51 | |
| DN3340_c0_g1_i4 | PRR | Scavenger Receptor Class B | RPRC013775 | 0.00 | 0.03 | -1.20 | 0.25 | -0.63 | 0.77 | |
| DN3340_c0_g1_i5 | PRR | Scavenger Receptor Class B | RPRC013775 | -0.13 | 0.28 | -0.97 | 0.34 | -0.53 | 0.49 | |
| DN3340_c0_g1_i6 | PRR | Scavenger Receptor Class B | RPRC013775 | -0.67 | 0.47 | -0.25 | -0.02 | 0.58 | -0.59 | |
| DN3340_c0_g1_i8 | PRR | Scavenger Receptor Class B | RPRC013775 | 0.04 | 0.23 | -1.38 | 0.21 | -0.43 | 0.60 | |
| DN3340_c0_g1_i9 | PRR | Scavenger Receptor Class B | RPRC013775 | -1.46 | 0.69 | 0.31 | -0.58 | 0.64 | -0.84 | |
| DN3455_c0_g1_i1 | PRR | Scavenger Receptor Class B | RPRC007349 | 0.24 | -0.19 | -0.29 | -0.29 | 0.14 | 0.26 | |
| DN3704_c0_g2_i10 | PRR | Clip-domain Serine Protease stubble/rpSP35 | RPRC004789 | -3.08 | 1.09 | -1.95 | -1.91 | 0.29 | 1.00 | |
| DN3704_c0_g2_i11 | PRR | Clip-domain Serine Protease stubble/rpSP35 | RPRC004789 | -1.47 | 1.01 | -1.18 | 1.02 | -1.32 | -0.42 | |
| DN3704_c0_g2_i13 | PRR | Clip-domain Serine Protease stubble/rpSP35 | RPRC004789 | 0.61 | -1.33 | 0.04 | -0.86 | 0.48 | 0.13 | |
| DN3704_c0_g2_i2 | PRR | Clip-domain Serine Protease stubble/rpSP35 | RPRC004789 | -2.36 | 1.37 | #NUM! | 1.27 | -1.36 | -1.29 | |
| DN3704_c0_g2_i8 | PRR | Clip-domain Serine Protease stubble/rpSP35 | RPRC004789 | -0.95 | -0.78 | -6.56 | 1.01 | -0.38 | 1.08 | |
| DN402_c1_g1_i1 | PRR | Clip-domain Serine Protease-SNAKE/rpSP45 | RPRC010197 | 0.24 | 0.16 | -1.23 | -1.33 | -0.20 | 1.01 | |
| DN402_c1_g1_i2 | PRR | Clip-domain Serine Protease-SNAKE/rpSP45 | RPRC001155 | -0.52 | -0.07 | 0.17 | 0.56 | -0.34 | -0.06 | |
| DN402_c1_g1_i3 | PRR | Clip-domain Serine Protease-SNAKE/rpSP45 | RPRC010197 | -0.47 | -0.14 | -0.48 | 0.25 | 0.12 | 0.46 | |
| DN402_c1_g1_i4 | PRR | Clip-domain Serine Protease-SNAKE/rpSP45 | RPRC010197 | 1.02 | -2.56 | -0.03 | -2.47 | -0.11 | 0.78 | |
| DN4830_c0_g1_i1 | PRR | Clip-domain Serine Protease-Stubble/rpSP48 | RPRC002919 | -1.51 | 0.67 | 0.03 | 0.12 | -0.61 | 0.38 | |
| DN4830_c0_g1_i2 | PRR | Clip-domain Serine Protease-Stubble/rpSP48 | RPRC002919 | -0.10 | 0.23 | -0.66 | 0.18 | -0.31 | 0.40 | |
| DN4830_c0_g1_i3 | PRR | Clip-domain Serine Protease-Stubble/rpSP48 | RPRC002919 | 0.30 | 0.04 | 0.51 | -0.57 | 0.20 | -1.02 | |
| DN5076_c0_g1_i1 | PRR | C-Type-lectin | RPRC000618 | 0.02 | 0.38 | -0.57 | 0.19 | -0.03 | -0.17 | |
| DN5251_c0_g1_i5 | PRR | C-Type-lectin | NA | -0.31 | 0.68 | -0.20 | 0.02 | -0.57 | 0.04 | |
| DN5251_c0_g1_i6 | PRR | C-Type-lectin | NA | -0.02 | -0.02 | -0.05 | 0.18 | 0.04 | -0.15 | |
| DN5347_c0_g1_i1 | PRR | C-Type-lectin | RPRC009440 | -0.17 | 0.27 | -0.15 | 0.05 | 0.01 | -0.06 | |
| DN6160_c0_g1_i2 | PRR | Scavenger Receptor Class B | NA | 0.49 | -0.68 | 0.21 | -0.28 | 0.57 | -0.97 | |
| DN6160_c0_g1_i3 | PRR | Scavenger Receptor Class B | NA | 0.00 | -0.58 | #NUM! | 0.77 | 0.92 | -0.45 | |
| DN6291_c0_g1_i12 | PRR | PGRP | RPRC012777 | -1.13 | -0.29 | 0.15 | -1.46 | 0.48 | 0.89 | |
| DN6422_c1_g1_i1 | PRR | Scavenger Receptor Class B | RPRC015098 | -0.85 | 0.55 | -0.98 | 0.54 | -0.09 | 0.11 | |
| DN6422_c1_g1_i3 | PRR | Scavenger Receptor Class B | RPRC015098 | 0.42 | -0.34 | 0.91 | -1.72 | 0.12 | -0.71 | |
| DN6422_c1_g1_i4 | PRR | Scavenger Receptor Class B | NA | -0.49 | -0.14 | -0.65 | 0.48 | 0.08 | 0.37 | |
| DN6422_c1_g1_i5 | PRR | Scavenger Receptor Class B | RPRC015098 | 0.13 | 0.61 | 0.09 | -1.19 | 0.40 | -0.83 | |
| DN6835_c0_g1_i1 | PRR | Phenoloxidase-activating factor 2/rpSP58 | RPRC005400 | 0.05 | 0.05 | -0.07 | 0.00 | -0.22 | 0.16 | |
| DN6835_c0_g1_i11 | PRR | Phenoloxidase-activating factor 2/rpSP58 | RPRC005400 | -0.56 | 0.46 | -0.87 | 0.38 | -0.30 | 0.36 | |
| DN6835_c0_g1_i13 | PRR | Phenoloxidase-activating factor 2/rpSP58 | RPRC005400 | -0.96 | 0.78 | -0.88 | 0.25 | -0.38 | 0.34 | |
| DN6835_c0_g1_i14 | PRR | Phenoloxidase-activating factor 2 | NA | -0.54 | 0.47 | -0.76 | -0.19 | -0.40 | 0.77 | |
| DN6835_c0_g1_i16 | PRR | Phenoloxidase-activating factor 2/rpSP58 | RPRC005400 | -0.83 | 0.82 | -1.00 | 0.66 | -0.41 | -0.25 | |
| DN6835_c0_g1_i17 | PRR | Phenoloxidase-activating factor 2/rpSP58 | RPRC005400 | -0.86 | 0.87 | -0.94 | 0.75 | -0.45 | -0.54 | |
| DN6835_c0_g1_i3 | PRR | Phenoloxidase-activating factor 2/rpSP58 | RPRC005400 | -0.59 | 0.70 | -1.57 | 0.45 | 0.45 | -0.64 | |
| DN6835_c0_g1_i5 | PRR | Phenoloxidase-activating factor 2/rpSP58 | RPRC005400 | -0.09 | 0.06 | -0.60 | -0.54 | -0.30 | 0.90 | |
| DN693_c0_g1_i1 | PRR | Dscam | RPRC006837 | -0.02 | 0.12 | -0.52 | -0.39 | -0.21 | 0.68 | |
| DN693_c0_g1_i26 | PRR | Dscam | RPRC006837 | 0.77 | -2.02 | -0.10 | -1.63 | -1.26 | 1.24 | |
| DN693_c0_g1_i32 | PRR | Dscam | RPRC006837 | 0.26 | -1.89 | -2.11 | -3.02 | -3.23 | 2.03 | |
| DN693_c0_g1_i39 | PRR | Dscam | RPRC006837 | 0.13 | -0.20 | 0.53 | -0.11 | 0.14 | -0.84 | |
| DN693_c0_g1_i40 | PRR | Dscam | RPRC006837 | -0.33 | -0.14 | 0.43 | 0.01 | 0.36 | -0.59 | |
| DN693_c0_g1_i46 | PRR | Dscam | RPRC006837 | 0.29 | -0.24 | 0.45 | 0.11 | 0.02 | -1.07 | |
| DN693_c0_g1_i49 | PRR | Dscam | RPRC006837 | 0.45 | 0.06 | 0.37 | -0.84 | 0.25 | -0.84 | |
| DN693_c0_g1_i51 | PRR | Dscam | RPRC006837 | -0.43 | -0.34 | 0.01 | -0.22 | -1.11 | 1.10 | |
| DN693_c0_g1_i65 | PRR | Dscam | RPRC006837 | -0.32 | -0.23 | 0.10 | -0.06 | -0.97 | 0.85 | |
| DN693_c0_g1_i66 | PRR | Dscam | RPRC006837 | 0.07 | -0.02 | 0.35 | -0.13 | 0.36 | -1.06 | |
| DN89_c0_g2_i1 | PRR | Clip-domain Serine Protease/rpSP63 | RPRC013717 | 0.34 | -0.23 | 0.17 | -0.02 | -0.78 | 0.24 | |
| DN9240_c1_g2_i1 | PRR | C-Type-lectin | RPRC000478 | -0.25 | -0.13 | 0.09 | 0.07 | 0.05 | 0.14 | |
| DN9965_c0_g1_i1 | PRR | Clotting factor B/rpSP66 | RPRC009335 | -1.39 | 1.09 | -0.62 | 0.44 | -0.22 | -0.69 | |
| DN11946_c0_g1_i1 | RNA interference | Armitage | RPRC000215 | 1.01 | -3.21 | -0.12 | -1.35 | -1.04 | 1.06 | |
| DN1244_c0_g1_i11 | RNA interference | Argonaute-1 | RPRC011389/RPRC013696 | -0.19 | 0.04 | -0.54 | 0.09 | 0.00 | 0.43 | |
| DN1244_c0_g1_i13 | RNA interference | Argonaute-1 | RPRC013696 | -0.97 | 0.23 | -0.17 | 0.35 | 0.57 | -0.58 | |
| DN1244_c0_g1_i15 | RNA interference | Argonaute-1 | RPRC013696 | -0.02 | -0.07 | -0.22 | 0.20 | 0.05 | 0.03 | |
| DN1244_c0_g1_i16 | RNA interference | Argonaute-1 | RPRC011389/RPRC013696 | 1.27 | -0.70 | -2.19 | 0.46 | -0.77 | -0.34 | |
| DN1244_c0_g1_i2 | RNA interference | Argonaute-1 | RPRC013696 | -0.11 | 0.03 | -0.49 | 0.15 | 0.13 | 0.18 | |
| DN1244_c0_g1_i4 | RNA interference | Argonaute-1 | RPRC013696 | 0.72 | -0.73 | 0.56 | -4.44 | -0.79 | 0.73 | |
| DN1244_c0_g1_i8 | RNA interference | Argonaute-1 | RPRC011389/RPRC013696 | -0.47 | -0.03 | -0.23 | 0.12 | -0.11 | 0.52 | |
| DN1244_c0_g1_i9 | RNA interference | Argonaute-1 | RPRC011389/RPRC013696 | -0.32 | 0.24 | -0.62 | 0.32 | -0.03 | 0.20 | |
| DN1385_c2_g1_i2 | RNA interference | Loqs | RPRC000198 | 0.64 | -0.92 | 0.26 | -0.41 | -0.20 | 0.14 | |
| DN1385_c2_g1_i3 | RNA interference | Loqs | RPRC000198 | 0.92 | -0.62 | 0.05 | -0.83 | -0.57 | 0.24 | |
| DN14289_c0_g1_i1 | RNA interference | Drosha | RPRC013112 | 0.07 | 1.10 | 0.05 | -1.79 | 0.49 | -3.50 | |
| DN14289_c0_g1_i12 | RNA interference | Drosha | RPRC013084/RPRC013112 | 0.02 | 1.09 | 0.11 | -1.74 | 0.50 | -3.79 | |
| DN14289_c0_g1_i13 | RNA interference | Drosha | RPRC013112 | 0.22 | -0.08 | 0.28 | 0.03 | 0.05 | -0.69 | |
| DN14289_c0_g1_i17 | RNA interference | Drosha | RPRC013084/RPRC013112 | -0.17 | 0.28 | -0.29 | 0.20 | 0.14 | -0.27 | |
| DN14289_c0_g1_i3 | RNA interference | Drosha | RPRC013084/RPRC013112 | 0.47 | -0.18 | 0.28 | 0.11 | 0.01 | -1.19 | |
| DN2448_c0_g1_i1 | RNA interference | Argonaute-2 | RPRC002460 | 0.18 | -0.23 | -0.50 | 0.12 | 0.20 | 0.11 | |
| DN2448_c0_g1_i2 | RNA interference | Argonaute-2 | RPRC002460 | 0.05 | -0.93 | -0.22 | 0.08 | 0.43 | 0.24 | |
| DN2448_c0_g1_i3 | RNA interference | Argonaute-2 | RPRC002460 | 0.30 | -0.70 | -0.05 | -0.21 | 0.03 | 0.38 | |
| DN405_c0_g1_i11 | RNA interference | Dicer-2 | RPRC013872 | -0.50 | 0.28 | -0.32 | 0.16 | 0.06 | 0.15 | |
| DN405_c0_g1_i12 | RNA interference | Dicer-2 | RPRC013872 | 0.38 | 0.00 | -0.78 | 0.14 | -0.11 | 0.12 | |
| DN405_c0_g1_i17 | RNA interference | Dicer-2 | RPRC013872 | -0.40 | 0.36 | 0.01 | -0.34 | 0.12 | 0.10 | |
| DN405_c0_g1_i18 | RNA interference | Dicer-2 | RPRC013872 | 0.54 | -0.34 | -0.55 | 0.62 | -0.23 | -0.53 | |
| DN405_c0_g1_i20 | RNA interference | Dicer-2 | RPRC013872 | 0.38 | -0.14 | -1.03 | 0.83 | -0.54 | -0.26 | |
| DN405_c0_g1_i25 | RNA interference | Dicer-2 | RPRC013872 | 0.60 | 0.17 | -0.34 | -0.18 | -0.13 | -0.36 | |
| DN405_c0_g1_i34 | RNA interference | Dicer-2 | RPRC013872 | 0.46 | -0.49 | -0.39 | -0.20 | -0.48 | 0.64 | |
| DN405_c0_g1_i35 | RNA interference | Dicer-2 | RPRC013872 | -0.58 | -0.11 | -1.08 | 0.98 | 0.39 | -0.62 | |
| DN405_c0_g1_i37 | RNA interference | Dicer-2 | RPRC013872 | -0.58 | 0.00 | 0.64 | 0.05 | 0.47 | -1.49 | |
| DN405_c0_g1_i40 | RNA interference | Dicer-2 | RPRC013872 | 0.75 | -0.88 | 0.01 | -1.26 | -0.40 | 0.67 | |
| DN405_c0_g1_i49 | RNA interference | Dicer-2 | RPRC013872 | 0.50 | -0.42 | -0.44 | -0.06 | -0.59 | 0.56 | |
| DN405_c0_g1_i8 | RNA interference | Dicer-2 | RPRC013872 | 0.44 | -0.30 | -0.50 | -0.05 | -0.37 | 0.47 | |
| DN4251_c0_g1_i1 | RNA interference | Piwi | RPRC000252 | 0.31 | -0.68 | -0.20 | -1.05 | 0.44 | 0.52 | |
| DN4334_c2_g1_i1 | RNA interference | Argonaute-3 | RPRC013054 | 0.27 | -0.72 | -0.18 | -0.26 | 0.06 | 0.51 | |
| DN531_c2_g2_i2 | RNA interference | Pasha | RPRC000304 | -0.03 | 0.03 | -0.12 | 0.43 | -1.13 | 0.35 | |
| DN531_c2_g2_i3 | RNA interference | Pasha | RPRC000304 | -0.79 | 0.73 | -1.16 | 0.04 | 0.71 | -0.60 | |
| DN531_c2_g2_i5 | RNA interference | Pasha | RPRC000304 | -0.73 | 0.44 | -0.51 | 0.72 | -0.21 | -0.29 | |
| DN6001_c0_g1_i1 | RNA interference | Dicer-1 | RPRC002485 | 0.87 | -0.49 | 1.38 | -1.56 | -1.58 | -2.39 | |
| DN7853_c0_g1_i1 | RNA interference | R2d2 | RPRC000119 | 0.07 | -0.09 | 0.25 | 0.00 | 0.03 | -0.32 | |
| DN7853_c0_g1_i10 | RNA interference | R2d2 | RPRC000119 | 0.08 | -0.07 | 0.24 | 0.07 | 0.08 | -0.52 | |
| DN7853_c0_g1_i12 | RNA interference | R2d2 | RPRC000119 | -0.02 | -0.20 | -0.28 | -0.50 | -0.17 | 0.78 | |
| DN7853_c0_g1_i20 | RNA interference | R2d2 | RPRC000119 | -0.94 | -0.18 | 0.31 | 0.81 | -1.05 | 0.16 | |
| DN7853_c0_g1_i23 | RNA interference | R2d2 | RPRC000119 | 1.62 | -0.42 | -1.05 | 0.29 | -1.92 | -2.21 | |
| DN7853_c0_g1_i3 | RNA interference | R2d2 | RPRC000119 | -0.10 | 0.50 | -0.94 | 0.09 | -0.14 | 0.21 | |
| DN7853_c0_g1_i6 | RNA interference | R2d2 | RPRC000119 | -0.04 | -0.30 | -0.38 | -0.52 | -0.08 | 0.85 | |
| DN7853_c0_g1_i9 | RNA interference | R2d2 | RPRC000119 | -0.01 | -0.02 | -0.21 | 0.15 | -0.08 | 0.15 | |
| DN16570_c0_g2_i1 | Toll signaling pathway | Tube | RPRC005871 | 0.57 | 0.00 | 0.08 | 0.12 | -0.25 | -0.90 | |
| DN16570_c0_g2_i5 | Toll signaling pathway | Tube | RPRC005871 | -0.25 | 0.13 | -0.75 | 0.29 | -0.33 | 0.54 | |
| DN16570_c0_g2_i6 | Toll signaling pathway | Tube | RPRC005871 | -0.54 | -3.31 | 1.01 | 0.83 | -4.03 | 0.44 | |
| DN16570_c0_g2_i9 | Toll signaling pathway | Tube | RPRC005871 | -0.21 | 0.21 | -0.78 | 0.40 | -0.23 | 0.30 | |
| DN1774_c0_g1_i12 | Toll signaling pathway | GNBP | RPRC01176/ RPRC011769 | -0.11 | 0.13 | -0.60 | -0.08 | -0.26 | 0.62 | |
| DN1774_c0_g1_i14 | Toll signaling pathway | GNBP | RPRC01176/ RPRC011769 | -0.03 | -0.72 | -0.50 | 0.04 | -0.61 | 1.02 | |
| DN1774_c0_g1_i15 | Toll signaling pathway | GNBP | RPRC01176/ RPRC011769 | -0.59 | -0.11 | -1.12 | -0.40 | -0.41 | 1.29 | |
| DN1774_c0_g1_i4 | Toll signaling pathway | GNBP | RPRC01176/ RPRC011769 | -0.03 | 0.05 | -0.42 | 0.25 | -0.29 | 0.30 | |
| DN1774_c0_g1_i6 | Toll signaling pathway | GNBP | RPRC01176/ RPRC011769 | 0.45 | 0.72 | -0.54 | -0.40 | -0.39 | -0.36 | |
| DN1774_c0_g1_i7 | Toll signaling pathway | GNBP | RPRC01176/ RPRC011769 | -0.03 | 0.23 | -0.56 | 0.07 | -0.28 | 0.38 | |
| DN30991_c0_g1_i1 | Toll signaling pathway | Pelle | RPRC005685 | -0.03 | 0.17 | 0.15 | -0.02 | -0.30 | 0.49 | |
| DN3547_c0_g1_i1 | Toll signaling pathway | Toll | RPRC009262 | -1.78 | 1.17 | -1.45 | 0.18 | -0.08 | 0.03 | |
| DN356_c0_g1_i1 | Toll signaling pathway | Dorsal | ABU96698/RPRC003790 | -1.86 | 1.21 | -1.43 | 1.01 | -1.03 | -0.89 | |
| DN356_c0_g1_i10 | Toll signaling pathway | Dorsal | ABU96698/RPRC003790 | 1.00 | -3.32 | -0.07 | -0.93 | 0.45 | 0.08 | |
| DN356_c0_g1_i3 | Toll signaling pathway | Dorsal | ABU96698/RPRC003790 | -2.15 | 1.37 | -1.86 | 0.06 | 0.00 | -0.20 | |
| DN356_c0_g1_i6 | Toll signaling pathway | Dorsal | ABU96698/RPRC003790 | -1.93 | 1.18 | -1.30 | 0.62 | -0.50 | -0.29 | |
| DN4905_c0_g1_i11 | Toll signaling pathway | Persephone/rpSP49 | RPRC010532 | -0.06 | 0.46 | -1.14 | 0.01 | -0.66 | 0.65 | |
| DN4905_c0_g1_i5 | Toll signaling pathway | Persephone/rpSP49 | RPRC010532 | -1.28 | 0.25 | -0.65 | 0.67 | -0.17 | 0.36 | |
| DN4905_c0_g1_i6 | Toll signaling pathway | Persephone/rpSP49 | RPRC010532 | -0.22 | 0.25 | -0.83 | 0.31 | -0.41 | 0.49 | |
| DN4905_c0_g1_i7 | Toll signaling pathway | Persephone/rpSP49 | RPRC010532 | -0.03 | 0.22 | -0.39 | -0.17 | -0.25 | 0.45 | |
| DN5625_c0_g1_i1 | Toll signaling pathway | Myd88 | RPRC009785 | -0.66 | -0.91 | -1.80 | -0.17 | 0.80 | 0.94 | |
| DN8630_c4_g1_i3 | Toll signaling pathway | Spz | RPRC002634 | -0.51 | -0.19 | -0.45 | 0.65 | -0.55 | 0.53 | |
| DN8630_c4_g1_i4 | Toll signaling pathway | Spz | RPRC002634 | 0.16 | 0.29 | -0.10 | 0.42 | -0.64 | -0.41 | |
| DN9926_c0_g1_i1 | Toll signaling pathway | Cactus | RPRC017349 | 0.29 | -0.18 | 0.46 | -0.05 | 0.18 | -1.25 | |
| DN9926_c0_g1_i10 | Toll signaling pathway | Cactus | RPRC017349 | 0.11 | -0.20 | 0.05 | 0.03 | 0.73 | -1.59 | |
| DN9926_c0_g1_i11 | Toll signaling pathway | Cactus | RPRC017349 | 0.12 | 0.10 | 0.00 | 0.01 | 0.48 | -1.17 | |
| DN9926_c0_g1_i18 | Toll signaling pathway | Cactus | RPRC017349 | -0.30 | -0.20 | 0.85 | 0.30 | 0.12 | -2.28 | |
| DN9926_c0_g1_i2 | Toll signaling pathway | Cactus | RPRC017349 | 0.71 | -0.93 | -0.23 | 0.63 | -0.45 | -0.51 | |
| DN9926_c0_g1_i21 | Toll signaling pathway | Cactus | RPRC017349 | 0.17 | 0.06 | 0.20 | 0.19 | 0.13 | -1.16 | |
| DN9926_c0_g1_i22 | Toll signaling pathway | Cactus | RPRC017349 | 0.45 | 0.13 | 0.37 | -0.57 | 0.59 | -3.89 | |
| DN9926_c0_g1_i23 | Toll signaling pathway | Cactus | RPRC017349 | -1.07 | 0.73 | -0.77 | 0.14 | -0.07 | 0.31 | |
| DN9926_c0_g1_i5 | Toll signaling pathway | Cactus | RPRC017349 | -0.83 | 0.65 | -0.65 | 0.13 | 0.04 | 0.14 | |
| DN9926_c0_g1_i9 | Toll signaling pathway | Cactus | RPRC017349 | -2.19 | 0.98 | -0.64 | 0.55 | -0.11 | -0.36 | |
| DN33_c1_g2_i16 | IMD signaling pathway | PGRP | rpRPRC-LC/LAa'/RPRC017796/RPRC017673 | 1.33 |  | 0.51 |  | -1.37 | 0.75 | |
| DN18198_c0_g1_i1 | Wound | DUOX | RPRC004883 | 0.15 | -0.22 | 0.05 | 0.25 | -0.10 | -0.19 | |

Table S3. Differentially expressed (DE) immune transcripts in the fat body of *Rhodnius prolixus.* Immune transcripts with DE are listed, Log2 fold change values were calculated using as a baseline the reference condition values

| ID | | Name | | Reference condition | | Contrasting condition | | Log2FC | | FDR p-value | |
| --- | --- | --- | --- | --- | --- | --- | --- | --- | --- | --- | --- |
| DN11689_c0_g1_i14 | Chitinase | | PBS-8h | | Ec-8h | | 3.62 | | 7.11E-03 | |  |
| DN11689_c0_g1_i34 | Chitinase | | Ec-8h | | Ec-24h | | -4.33 | | 2.40E-03 | |  |
| DN11689_c0_g1_i5 | Chitinase | | PBS-8h | | PBS-24h | | 4.01 | | 1.19E-02 | |  |
| DN1204_c1_g2_i2 | A-kinase anchor protein 1 | | Ec-8h | | Sa-8h | | -2.14 | | 1.92E-02 | |  |
| DN13091_c0_g1_i3 | Chitotriosidase-1 | | PBS-8h | | PBS-24h | | 4.81 | | 2.34E-02 | |  |
| DN1470_c2_g1_i1 | Eiger | | Ec-8h | | Sa-8h | | -4.14 | | 2.17E-04 | |  |
| DN1774_c0_g1_i15 | GNBP | | PBS-8h | | PBS-24h | | 8.13 | | 8.09E-03 | |  |
| DN1774_c0_g1_i3 | GNBP | | PBS-8h | | Ec-8h | | 2.91 | | 3.02E-03 | |  |
| DN1774_c0_g1_i3 | GNBP | | PBS-24h | | Sa-24h | | -2.31 | | 1.42E-02 | |  |
| DN1774_c0_g1_i6 | GNBP | | PBS-8h | | Ec-8h | | 2.41 | | 7.07E-03 | |  |
| DN1774_c0_g1_i6 | GNBP | | Ec-8h | | Ec-24h | | -1.61 | | 2.78E-02 | |  |
| DN1774_c0_g1_i6 | GNBP | | PBS-24h | | Sa-24h | | -2.05 | | 3.53E-02 | |  |
| DN20_c2_g2_i1 | Defensin | | PBS-8h | | PBS-24h | | -3.05 | | 7.42E-03 | |  |
| DN20_c3_g1_i2 | Defensin | | Ec-8h | | Sa-8h | | 1.72 | | 4.37E-03 | |  |
| DN20238_c0_g1_i5 | Dnr1 | | PBS-8h | | Ec-8h | | -2.56 | | 1.79E-03 | |  |
| DN2263_c0_g1_i13 | Defensin | | Ec-8h | | Sa-8h | | 2.27 | | 3.86E-04 | |  |
| DN2263_c0_g1_i16 | Defensin | | Ec-8h | | Sa-8h | | 1.78 | | 4.67E-02 | |  |
| DN2263_c0_g1_i9 | Defensin | | Ec-8h | | Sa-8h | | 2.23 | | 1.90E-03 | |  |
| DN2304_c1_g1_i48 | Scavenger Receptor Class B | | PBS-24h | | Sa-24h | | -2.14 | | 2.31E-02 | |  |
| DN2418_c0_g1_i1 | Mariner | | PBS-8h | | PBS-24h | | -5.05 | | 2.40E-02 | |  |
| DN2418_c0_g1_i12 | Mariner | | Ec-8h | | Sa-8h | | 2.07 | | 1.19E-03 | |  |
| DN2418_c0_g1_i5 | Mariner | | Ec-8h | | Sa-8h | | 1.78 | | 2.55E-02 | |  |
| DN2418_c0_g1_i6 | Mariner | | PBS-8h | | Ec-8h | | -3.24 | | 1.56E-02 | |  |
| DN2658_c0_g1_i16 | GNBP | | PBS-24h | | Sa-24h | | -2.62 | | 5.72E-03 | |  |
| DN2658_c0_g1_i16 | GNBP | | PBS-8h | | PBS-24h | | 2.39 | | 9.01E-03 | |  |
| DN2658_c0_g1_i16 | GNBP | | PBS-8h | | Ec-8h | | 2.51 | | 1.67E-02 | |  |
| DN2846_c0_g1_i19 | Titin | | Sa-8h | | Sa-24h | | -2.56 | | 3.99E-02 | |  |
| DN2846_c0_g1_i6 | Titin | | Sa-8h | | Sa-24h | | -2.37 | | 4.93E-02 | |  |
| DN297_c0_g2_i11 | Clip-SP/rpSP30 | | PBS-8h | | Ec-8h | | -2.97 | | 9.75E-03 | |  |
| DN297_c0_g2_i21 | Clip-SP/ rpSP30 | | PBS-8h | | Ec-8h | | 4.32 | | 1.46E-02 | |  |
| DN297_c0_g2_i22 | Clip-SP/ rpSP30 | | PBS-8h | | Ec-8h | | -3.57 | | 8.29E-03 | |  |
| DN297_c0_g2_i25 | Clip-SP/ rpSP30 | | PBS-8h | | Ec-8h | | -3.14 | | 1.62E-04 | |  |
| DN297_c0_g2_i25 | Clip-SP/ rpSP30 | | PBS-8h | | PBS-24h | | -2.53 | | 1.10E-03 | |  |
| DN297_c0_g2_i25 | Clip-SPrpSP30 | | Ec-8h | | Sa-8h | | 2.68 | | 2.46E-02 | |  |
| DN297_c0_g2_i27 | Clip-SP/rpSP30 | | PBS-8h | | PBS-24h | | -2.45 | | 4.99E-02 | |  |
| DN3262_c0_g1_i1 | rpSPI10a | | PBS-8h | | PBS-24h | | 3.61 | | 6.06E-04 | |  |
| DN3262_c0_g1_i1 | rpSPI10a | | PBS-8h | | Ec-8h | | 3.59 | | 2.52E-03 | |  |
| DN3262_c0_g1_i3 | rpSPI10b | | PBS-8h | | PBS-24h | | 3.42 | | 1.60E-02 | |  |
| DN3262_c0_g1_i5 | rpSPI10c | | PBS-8h | | PBS-24h | | 3.43 | | 2.08E-03 | |  |
| DN3262_c0_g1_i5 | rpSPI10c | | PBS-8h | | Ec-8h | | 3.55 | | 4.42E-03 | |  |
| DN3340_c0_g1_i6 | Scavenger Receptor Class B | | Ec-8h | | Sa-8h | | -10.16 | | 1.43E-03 | |  |
| DN3455_c0_g1_i1 |  | | PBS-8h | | Ec-8h | | 2.47 | | 4.77E-02 | |  |
| DN3991_c0_g2_i1 | Prolixicin | | Ec-8h | | Sa-8h | | 2.02 | | 3.29E-02 | |  |
| DN4045_c0_g1_i17 | Titin | | Sa-8h | | Sa-24h | | -2.40 | | 1.59E-02 | |  |
| DN4045_c0_g1_i23 | Titin | | Ec-8h | | Sa-8h | | 1.77 | | 3.01E-02 | |  |
| DN4113_c0_g1_i15 | Sequestosome | | Sa-8h | | Sa-24h | | -3.38 | | 1.67E-02 | |  |
| DN4180_c0_g1_i8 | IMD | | PBS-24h | | Sa-24h | | -1.71 | | 3.45E-02 | |  |
| DN5251_c0_g1_i5 | C-Type-lectin | | PBS-8h | | Ec-8h | | 2.81 | | 4.38E-03 | |  |
| DN5251_c0_g1_i5 | C-Type-lectin | | PBS-8h | | PBS-24h | | 2.46 | | 8.59E-03 | |  |
| DN7810_c0_g4_i1 | Peroxiredoxin | | Sa-8h | | Sa-24h | | -2.36 | | 3.78E-02 | |  |
| DN33_c1_g2_i16 | PGRP-LC/LAa' | | Ec-8h | | Sa-8h | | -11.70 | | 3.86E-04 | |  |
| DN33_c1_g2_i16 | PGRP-LC/LAa' | | PBS-24h | | Sa-24h | | -12.15 | | 4.57E-18 | |  |
| DN33_c1_g2_i16 | PGRP-LC/LAa' | | PBS-8h | | Ec-8h | | 12.66 | | 2.41E-10 | |  |
| DN33_c1_g2_i16 | PGRP-LC/LAa' | | PBS-8h | | PBS-24h | | 12.65 | | 1.84E-10 | |  |

Table S4. Statistically significant enriched Gene Ontology (GO) terms from pairwise comparisons. Enriched GO terms were found for the Gr- bacteria 8hpi and PBS 24hpi treatments when compared with the PBS 8hpi treatment, but not for other comparisons. BP: biological process, MF molecular function, CC: cellular compartment.

| Treatment | Expression change | GO_ID | GO name | Category | FDR |
| --- | --- | --- | --- | --- | --- |
| Gr- 8hpi | Decrease | GO:1903053 | Regulation of extracellular matrix organization | BP. | 4.64E-02 |
| Gr- 8hpi | Decrease | GO:0085029 | Extracellular matrix assembly | BP | 4.54E-02 |
| Gr- 8hpi | Decrease | GO:0015227 | Acyl carnitine transmembrane transporter activity | MF | 4.43E-02 |
| Gr- 8hpi | Decrease | GO:0003360 | Brainstem development | BP | 4.43E-02 |
| Gr- 8hpi | Decrease | GO:0008063 | Toll signaling pathway | BP | 4.40E-02 |
| Gr- 8hpi | Decrease | GO:0042632 | Cholesterol homeostasis | BP | 4.30E-02 |
| Gr- 8hpi | Decrease | GO:0055092 | Sterol homeostasis | BP | 4.30E-02 |
| Gr- 8hpi | Decrease | GO:0046390 | Ribose phosphate biosynthetic process | BP | 3.97E-02 |
| Gr- 8hpi | Decrease | GO:0009112 | Nucleobase metabolic process | BP | 3.93E-02 |
| Gr- 8hpi | Decrease | GO:0005604 | Basement membrane | CC | 3.84E-02 |
| Gr- 8hpi | Decrease | GO:0071711 | Basement membrane organization | BP | 3.81E-02 |
| Gr- 8hpi | Decrease | GO:0015819 | Lysine transport | BP | 3.77E-02 |
| Gr- 8hpi | Decrease | GO:0046112 | Nucleobase biosynthetic process | BP | 3.37E-02 |
| Gr- 8hpi | Decrease | GO:0009950 | Dorsal/ventral axis specification | BP | 3.37E-02 |
| Gr- 8hpi | Decrease | GO:0006844 | Acyl carnitine transport | BP | 3.36E-02 |
| Gr- 8hpi | Decrease | GO:0046654 | Tetrahydrofolate biosynthetic process | BP | 3.36E-02 |
| Gr- 8hpi | Decrease | GO:0009259 | Ribonucleotide metabolic process | BP | 3.32E-02 |
| Gr- 8hpi | Decrease | GO:0034383 | Low-density lipoprotein particle clearance | BP | 3.18E-02 |
| Gr- 8hpi | Decrease | GO:0089709 | L-histidine transmembrane transport | BP | 3.01E-02 |
| Gr- 8hpi | Decrease | GO:1902024 | L-histidine transport | BP | 3.01E-02 |
| Gr- 8hpi | Decrease | GO:1990575 | Mitochondrial L-ornithine transmembrane transport | BP | 3.01E-02 |
| Gr- 8hpi | Decrease | GO:0015817 | Histidine transport | BP | 3.01E-02 |
| Gr- 8hpi | Decrease | GO:0046100 | Hypoxanthine metabolic process | BP | 3.01E-02 |
| Gr- 8hpi | Decrease | GO:0034418 | Urate biosynthetic process | BP | 3.01E-02 |
| Gr- 8hpi | Decrease | GO:0009150 | Purine ribonucleotide metabolic process | BP | 2.91E-02 |
| Gr- 8hpi | Decrease | GO:0004329 | Formate-tetrahydrofolate ligase activity | MF | 2.80E-02 |
| Gr- 8hpi | Decrease | GO:0004643 | EC 2.1.2.3 | MF | 2.80E-02 |
| Gr- 8hpi | Decrease | GO:0003937 | IMP cyclohydrolase activity | MF | 2.80E-02 |
| Gr- 8hpi | Decrease | GO:0002189 | Ribose phosphate diphosphokinase complex | CC | 2.41E-02 |
| Gr- 8hpi | Decrease | GO:0004749 | Ribose phosphate diphosphokinase activity | MF | 2.41E-02 |
| Gr- 8hpi | Decrease | GO:0046391 | 5-phosphoribose 1-diphosphate metabolic process | BP | 2.41E-02 |
| Gr- 8hpi | Decrease | GO:0006015 | 5-phosphoribose 1-diphosphate biosynthetic process | BP | 2.41E-02 |
| Gr- 8hpi | Decrease | GO:0046452 | Dihydrofolate metabolic process | BP | 2.07E-02 |
| Gr- 8hpi | Decrease | GO:0008592 | Regulation of Toll signaling pathway | BP | 1.95E-02 |
| Gr- 8hpi | Decrease | GO:0097006 | Regulation of plasma lipoprotein particle levels | BP | 1.94E-02 |
| Gr- 8hpi | Decrease | GO:0070831 | Basement membrane assembly | BP | 1.81E-02 |
| Gr- 8hpi | Decrease | GO:0006144 | Purine nucleobase metabolic process | BP | 1.37E-02 |
| Gr- 8hpi | Decrease | GO:0046040 | IMP metabolic process | BP | 1.32E-02 |
| Gr- 8hpi | Decrease | GO:0006188 | IMP biosynthetic process | BP | 1.32E-02 |
| Gr- 8hpi | Decrease | GO:0019693 | Ribose phosphate metabolic process | BP | 1.29E-02 |
| Gr- 8hpi | Decrease | GO:0009113 | Purine nucleobase biosynthetic process | BP | 1.29E-02 |
| Gr- 8hpi | Decrease | GO:0009124 | Nucleoside monophosphate biosynthetic process | BP | 1.20E-02 |
| Gr- 8hpi | Decrease | GO:0031638 | Zymogen activation | BP | 1.20E-02 |
| Gr- 8hpi | Decrease | GO:1901657 | Glycosyl compound metabolic process | BP | 1.20E-02 |
| Gr- 8hpi | Decrease | GO:0016814 | Hydrolase activity acting on carbon-nitrogen | MF | 1.10E-02 |
| Gr- 8hpi | Decrease | GO:0009156 | Ribonucleoside monophosphate biosynthetic process | BP | 1.10E-02 |
| Gr- 8hpi | Decrease | GO:0046101 | Hypoxanthine biosynthetic process | BP | 1.10E-02 |
| Gr- 8hpi | Decrease | GO:0006164 | Purine nucleotide biosynthetic process | BP | 1.10E-02 |
| Gr- 8hpi | Decrease | GO:1901293 | Nucleoside phosphate biosynthetic process | BP | 1.05E-02 |
| Gr- 8hpi | Decrease | GO:0009165 | Nucleotide biosynthetic process | BP | 1.01E-02 |
| Gr- 8hpi | Decrease | GO:0042558 | Pteridine-containing compound metabolic process | BP | 1.00E-02 |
| Gr- 8hpi | Decrease | GO:0043436 | Oxoacid metabolic process | BP | 9.62E-03 |
| Gr- 8hpi | Decrease | GO:0005576 | Extracellular region | CC | 9.62E-03 |
| Gr- 8hpi | Decrease | GO:0005292 | High-affinity lysine transmembrane transporter activity | MF | 9.32E-03 |
| Gr- 8hpi | Decrease | GO:0005287 | High-affinity basic amino acid transmembrane transporter activity | MF | 9.32E-03 |
| Gr- 8hpi | Decrease | GO:0005289 | High-affinity arginine transmembrane transporter activity | MF | 9.32E-03 |
| Gr- 8hpi | Decrease | GO:1903401 | L-lysine transmembrane transport | BP | 9.32E-03 |
| Gr- 8hpi | Decrease | GO:1902022 | L-lysine transport | BP | 9.32E-03 |
| Gr- 8hpi | Decrease | GO:0010172 | Embryonic body morphogenesis | BP | 8.72E-03 |
| Gr- 8hpi | Decrease | GO:0006189 | '*De novo*' IMP biosynthetic process | BP | 8.27E-03 |
| Gr- 8hpi | Decrease | GO:0030246 | Carbohydrate binding | MF | 7.44E-03 |
| Gr- 8hpi | Decrease | GO:0006760 | Folic acid-containing compound metabolic process | BP | 7.44E-03 |
| Gr- 8hpi | Decrease | GO:0019752 | Carboxylic acid metabolic process | BP | 7.44E-03 |
| Gr- 8hpi | Decrease | GO:0110011 | Regulation of basement membrane organization | BP | 7.44E-03 |
| Gr- 8hpi | Decrease | GO:0009127 | Purine nucleoside monophosphate biosynthetic process | BP | 7.44E-03 |
| Gr- 8hpi | Decrease | GO:0009168 | Purine ribonucleoside monophosphate biosynthetic process | BP | 7.44E-03 |
| Gr- 8hpi | Decrease | GO:0031100 | Animal organ regeneration | BP | 7.44E-03 |
| Gr- 8hpi | Decrease | GO:0045752 | Positive regulation of Toll signaling pathway | BP | 7.44E-03 |
| Gr- 8hpi | Decrease | GO:0006013 | Mannose metabolic process | BP | 7.44E-03 |
| Gr- 8hpi | Decrease | GO:0006163 | Purine nucleotide metabolic process | BP | 6.64E-03 |
| Gr- 8hpi | Decrease | GO:0009116 | Nucleoside metabolic process | BP | 6.62E-03 |
| Gr- 8hpi | Decrease | GO:0019637 | Organophosphate metabolic process | BP | 6.18E-03 |
| Gr- 8hpi | Decrease | GO:2001197 | Basement membrane assembly. Embryonic body morphogenesis | BP | 5.98E-03 |
| Gr- 8hpi | Decrease | GO:0072522 | Purine-containing compound biosynthetic process | BP | 4.42E-03 |
| Gr- 8hpi | Decrease | GO:0009123 | Nucleoside monophosphate metabolic process | BP | 3.72E-03 |
| Gr- 8hpi | Decrease | GO:0009161 | Ribonucleoside monophosphate metabolic process | BP | 3.65E-03 |
| Gr- 8hpi | Decrease | GO:0006753 | Nucleoside phosphate metabolic process | BP | 3.58E-03 |
| Gr- 8hpi | Decrease | GO:0009117 | Nucleotide metabolic process | BP | 3.58E-03 |
| Gr- 8hpi | Decrease | GO:0009126 | Purine nucleoside monophosphate metabolic process | BP | 3.02E-03 |
| Gr- 8hpi | Decrease | GO:0009167 | Purine ribonucleoside monophosphate metabolic process | BP | 3.02E-03 |
| Gr- 8hpi | Decrease | GO:0044283 | Small molecule biosynthetic process | BP | 3.02E-03 |
| Gr- 8hpi | Decrease | GO:0046653 | Tetrahydrofolate metabolic process | BP | 3.00E-03 |
| Gr- 8hpi | Decrease | GO:0006082 | Organic acid metabolic process | BP | 3.00E-03 |
| Gr- 8hpi | Decrease | GO:0072521 | Purine-containing compound metabolic process | BP | 1.75E-03 |
| Gr- 8hpi | Decrease | GO:0019238 | Cyclohydrolase activity | MF | 1.74E-03 |
| Gr- 8hpi | Decrease | GO:0055086 | Nucleobase-containing small molecule metabolic process | BP | 1.74E-03 |
| Gr- 8hpi | Decrease | GO:0044281 | Small molecule metabolic process | BP | 2.45E-04 |
| Gr- 8hpi | Increase | GO:0042427 | Serotonin biosynthetic process | BP | 4.79E-02 |
| Gr- 8hpi | Increase | GO:0036468 | L-dopa decarboxylase activity | MF | 4.79E-02 |
| Gr- 8hpi | Increase | GO:0098700 | Neurotransmitter loading into synaptic vesicle | BP | 4.79E-02 |
| Gr- 8hpi | Increase | GO:1901162 | Primary amino compound biosynthetic process | BP | 4.79E-02 |
| Gr- 8hpi | Increase | GO:0009308 | Amine metabolic process | BP | 4.79E-02 |
| Gr- 8hpi | Increase | GO:0044106 | Cellular amine metabolic process | BP | 4.79E-02 |
| Gr- 8hpi | Increase | GO:0048737 | Imaginal disc-derived appendage development | BP | 4.16E-02 |
| Gr- 8hpi | Increase | GO:0007552 | Metamorphosis | BP | 4.16E-02 |
| Gr- 8hpi | Increase | GO:0005576 | Extracellular region | CC | 4.01E-02 |
| Gr- 8hpi | Increase | GO:0035114 | Imaginal disc-derived appendage morphogenesis | BP | 4.01E-02 |
| Gr- 8hpi | Increase | GO:0002683 | Negative regulation of immune system process | BP | 3.75E-02 |
| Gr- 8hpi | Increase | GO:0006576 | Cellular biogenic amine metabolic process | BP | 2.97E-02 |
| Gr- 8hpi | Increase | GO:0016831 | Carboxy-lyase activity | MF | 2.65E-02 |
| Gr- 8hpi | Increase | GO:0052314 | Phytoalexin metabolic process | BP | 2.65E-02 |
| Gr- 8hpi | Increase | GO:0033076 | Isoquinoline alkaloid metabolic process | BP | 2.65E-02 |
| Gr- 8hpi | Increase | GO:0046684 | Response to pyrethroid | BP | 2.65E-02 |
| Gr- 8hpi | Increase | GO:0007444 | Imaginal disc development | BP | 2.45E-02 |
| Gr- 8hpi | Increase | GO:0042416 | Dopamine biosynthetic process | BP | 8.88E-03 |
| Gr- 8hpi | Increase | GO:1901617 | Organic hydroxy compound biosynthetic process | BP | 4.64E-03 |
| Gr- 8hpi | Increase | GO:0006584 | Catecholamine metabolic process | BP | 3.75E-04 |
| Gr- 8hpi | Increase | GO:0009712 | Catechol-containing compound metabolic process | BP | 3.75E-04 |
| Gr- 8hpi | Increase | GO:0018958 | Phenol-containing compound metabolic process | BP | 2.79E-04 |
| Gr- 8hpi | Increase | GO:0042401 | Cellular biogenic amine biosynthetic process | BP | 2.39E-04 |
| Gr- 8hpi | Increase | GO:0009309 | Amine biosynthetic process | BP | 2.39E-04 |
| Gr- 8hpi | Increase | GO:0004058 | Aromatic-L-amino-acid decarboxylase activity | MF | 1.48E-05 |
| Gr- 8hpi | Increase | GO:0042423 | Catecholamine biosynthetic process | BP | 7.34E-06 |
| Gr- 8hpi | Increase | GO:0009713 | Catechol-containing compound biosynthetic process | BP | 7.34E-06 |
| Gr- 8hpi | Increase | GO:0046189 | Phenol-containing compound biosynthetic process | BP | 6.71E-07 |
| PBS 24hpi | Decrease | GO:0005319 | Lipid transporter activity | MF | 4.23E-02 |
| PBS 24hpi | Decrease | GO:0004044 | Amidophosphoribosyltransferase activity | MF | 4.23E-02 |
| PBS 24hpi | Decrease | GO:0046040 | IMP metabolic process | BP | 4.23E-02 |
| PBS 24hpi | Decrease | GO:0009113 | Purine nucleobase biosynthetic process | BP | 4.23E-02 |
| PBS 24hpi | Decrease | GO:0006144 | Purine nucleobase metabolic process | BP | 4.23E-02 |
| PBS 24hpi | Decrease | GO:0006189 | '*De novo*' IMP biosynthetic process | BP | 4.23E-02 |
| PBS 24hpi | Decrease | GO:0006188 | IMP biosynthetic process | BP | 4.23E-02 |
| PBS 24hpi | Increase | GO:0042438 | Melanin biosynthetic process | BP | 4.03E-02 |
| PBS 24hpi | Increase | GO:0031347 | Regulation of defense response | BP | 3.04E-02 |
| PBS 24hpi | Increase | GO:1901615 | Organic hydroxy compound metabolic process | BP | 2.93E-02 |
| PBS 24hpi | Increase | GO:0045087 | Innate immune response | BP | 2.93E-02 |
| PBS 24hpi | Increase | GO:0045751 | Negative regulation of Toll signaling pathway | BP | 2.64E-02 |
| PBS 24hpi | Increase | GO:0009607 | Response to biotic stimulus | BP | 2.33E-02 |
| PBS 24hpi | Increase | GO:0016831 | Carboxy-lyase activity | MF | 2.12E-02 |
| PBS 24hpi | Increase | GO:0051707 | Response to other organism | BP | 2.12E-02 |
| PBS 24hpi | Increase | GO:0002832 | Negative regulation of response to biotic stimulus | BP | 2.12E-02 |
| PBS 24hpi | Increase | GO:0045824 | Negative regulation of innate immune response | BP | 2.12E-02 |
| PBS 24hpi | Increase | GO:0043207 | Response to external biotic stimulus | BP | 2.12E-02 |
| PBS 24hpi | Increase | GO:0050776 | Regulation of immune response | BP | 1.34E-02 |
| PBS 24hpi | Increase | GO:0006584 | Catecholamine metabolic process | BP | 1.19E-02 |
| PBS 24hpi | Increase | GO:0009712 | Catechol-containing compound metabolic process | BP | 1.19E-02 |
| PBS 24hpi | Increase | GO:0050777 | Negative regulation of immune response | BP | 9.31E-03 |
| PBS 24hpi | Increase | GO:0042401 | Cellular biogenic amine biosynthetic process | BP | 7.61E-03 |
| PBS 24hpi | Increase | GO:0002831 | Regulation of response to biotic stimulus | BP | 7.61E-03 |
| PBS 24hpi | Increase | GO:0009309 | Amine biosynthetic process | BP | 7.61E-03 |
| PBS 24hpi | Increase | GO:0045088 | Regulation of innate immune response | BP | 6.54E-03 |
| PBS 24hpi | Increase | GO:0002752 | Cell surface pattern recognition receptor signaling pathway | BP | 3.74E-03 |
| PBS 24hpi | Increase | GO:0048526 | Imaginal disc-derived wing expansion | BP | 1.47E-03 |
| PBS 24hpi | Increase | GO:1901617 | Organic hydroxy compound biosynthetic process | BP | 6.95E-04 |
| PBS 24hpi | Increase | GO:0042423 | Catecholamine biosynthetic process | BP | 6.68E-04 |
| PBS 24hpi | Increase | GO:0018958 | Phenol-containing compound metabolic process | BP | 6.68E-04 |
| PBS 24hpi | Increase | GO:0009713 | Catechol-containing compound biosynthetic process | BP | 6.68E-04 |
| PBS 24hpi | Increase | GO:0004058 | Aromatic-L-amino-acid decarboxylase activity | MF | 3.50E-05 |
| PBS 24hpi | Increase | GO:0046189 | Phenol-containing compound biosynthetic process | BP | 9.06E-07 |


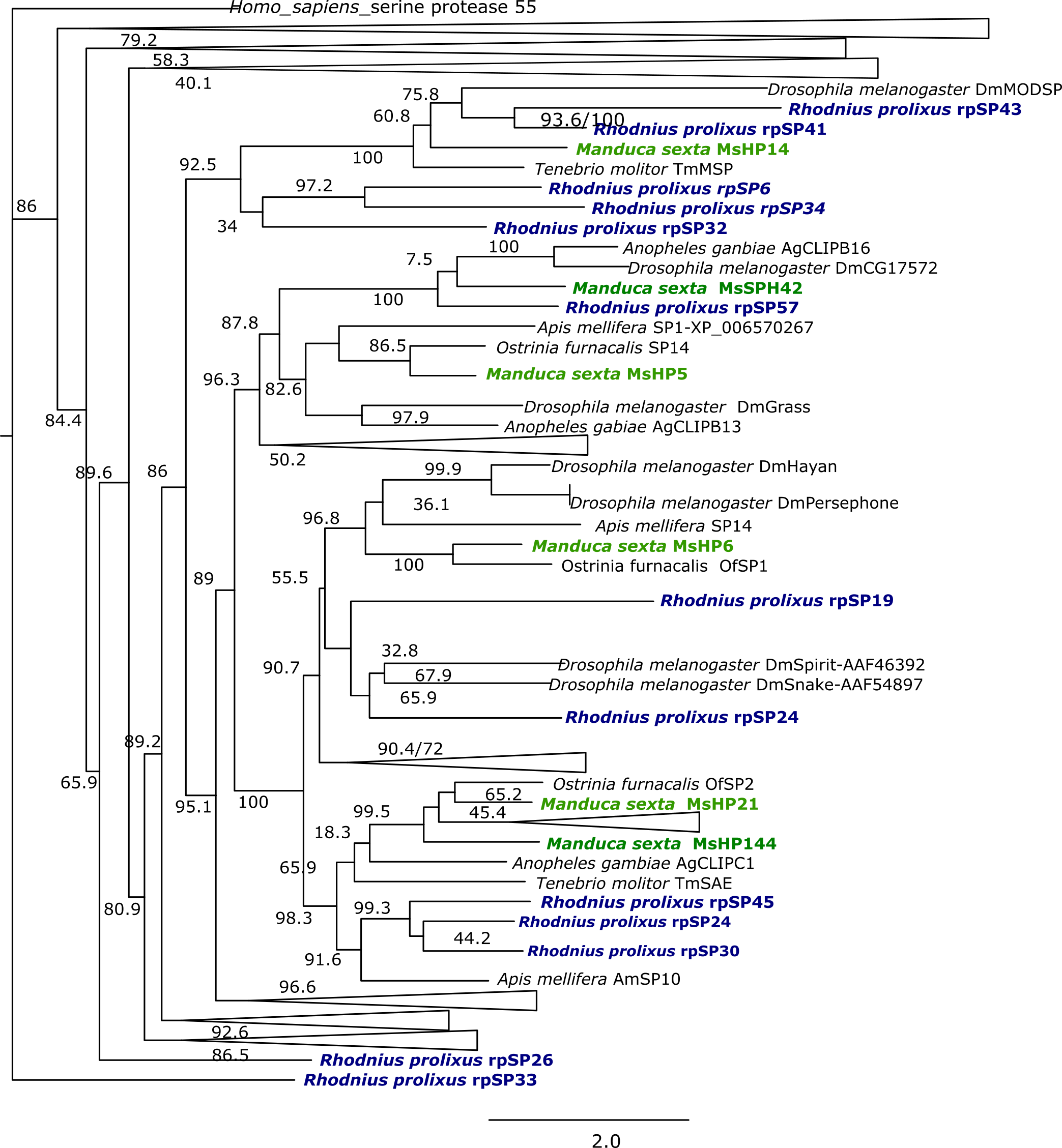


Fig S1. Maximum likelihood phylogenetic tree of serine proteases (SP) from selected insects. Multiple SP clades containing SPs from different species are formed. *Rhodnius prolixus* SPs (colored in blue) are distributed across the tree; some *R. prolixus* SP are clustered together with SP from *Manduca sexta* (colored in green) that participate in the Toll and melanization pathways. Some clades containing multiple species are collapsed for display purposes. Clade support is shown as percentage values of 1000 ultrafast bootstrap replicates.


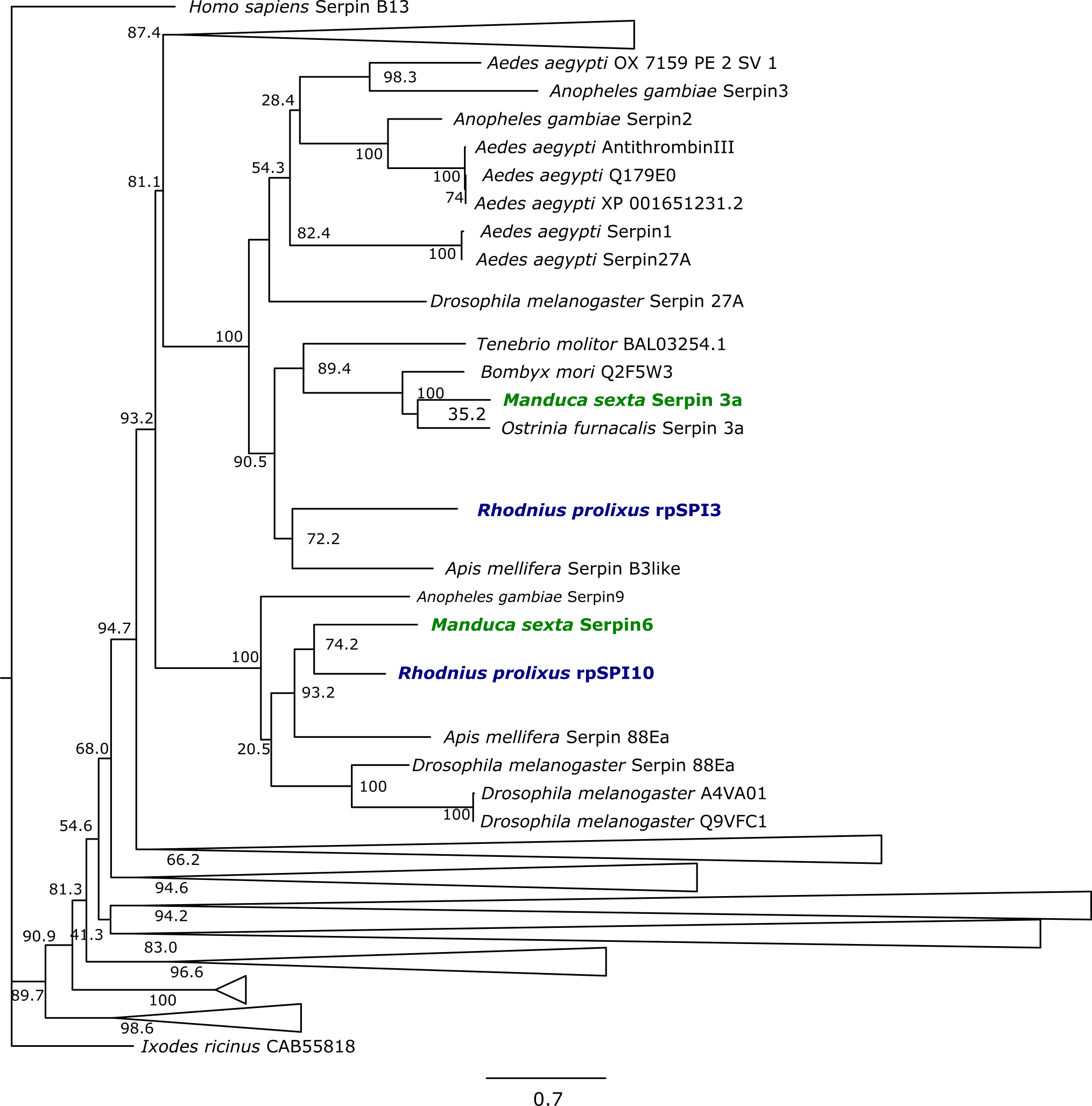


Fig S2. Maximum likelihood phylogenetic tree of serine protease inhibitors (SPI) from selected insects. Only a few SPI from *Rhodnius prolixus* (colored in blue) are clustered together with SP from *Manduca sexta* (colored in green) that participate in the Toll and melanization pathways. Some clades containing multiple species are collapsed for display purposes Clade support is shown as percentage values of 1000 ultrafast bootstrap replicates.

Table S5. *Rhodnius prolixus* serine proteases (SPs). List of SPs identified in a *R. prolixus* fat body tissue transcriptome. The closest ortholog to these SPs was identified in *Drosophila melanogaster* and *Manduca sexta* using phylogenetic analyses from Figure S1.

| Name | Vector base ID | Transcriptome ID | Closest ortholog in *Drosophila*  *melanogaster* | Closest  ortholog in *Manduca*  *sexta* |
| --- | --- | --- | --- | --- |
| rpSP1 | RPRC014822 | DN10315_c0_g1_i1 |  |  |
| rpSP2 | RPRC000150 | DN106_c1_g1_i2-6 |  |  |
| rpSP3 | RPRC002329 RPRC014497 | DN1083_c0_g1_i20 |  |  |
| rpSP4 | RPRC008099 | DN10836_c0_g1_i9 | CG8213 | SP142 |
| rpSP5-CLIP | RPRC005405 RPRC005420 | DN11528_c0_g1_i6-22 | CG6639 | SPH1/2 |
| rpSP6 | RPRC005759 | DN12201_c0_g1_i1-2 | ModSP | HP14 |
| rpSP7 | RPRC000075 | DN1227_c0_g1_i4 |  |  |
| rpSP8-CLIP | RPRC009383 | DN14442_c0_g1_i7-9 | CG7432 | SP52 |
| rpSP9-CLIP | RPRC003090 | DN1612_c0_g2_i44 | CG1299 | HP17 |
| rpSP10 | RPRC009294 | DN16221_c0_g1_i61 |  |  |
| rpSP11 | RPRC015053 | DN1626_c0_g1_i1 |  |  |
| rpSP12 | RPRC002410 | DN16689_c0_g1_i1 |  |  |
| rpSP13 | RPRC010335 RPRC008401 | DN17342_c0_g2_i1 |  |  |
| rpSP14 | RPRC010336 | DN1750_c0_g1_i7 |  |  |
| rpSP15 | RPRC014012 RPRC000890 | DN17651_c0_g2_i4 | Stubble | SP132/19 |
| rpSP16 | RPRC000107 | DN18201_c0_g1_i1 |  |  |
| rpSP17 | RPRC004608 | DN18297_c0_g1_i1 |  |  |
| rpSP18 | RPRC012541 | DN1874_c0_g1_i3 |  |  |
| rpSP19-CLIP | RPRC005654 | DN19140_c0_g2_i7 | Hayan,  Spirit,  Snake,  Persephone | HP6 |
| rpSP20 | RPRC000049 | DN1932_c0_g1_i14 |  |  |
| rpSP21 | RPRC015053 | DN1989_c0_g1_i17-25 |  |  |
| rpSP22 | RPRC000610 | DN21223_c0_g1_i1 |  |  |
| rpSP23 | RPRC012649 | DN21511_c0_g1_i12 |  |  |
| rpSP24 | RPRC005358 | DN2264_c0_g1_i7 | Hayan,  Spirit,  Snake,  Persephone | HP6 |
| rpSP25-CLIP | RPRC009415 | DN2394_c0_g1_i3 | Masquerade | SPH53 |
| rpSP26 | RPRC003330 | DN24225_c0_g3_i1 |  |  |
| rpSP27 | RPRC010285 | DN2516_c0_g1_i3 |  |  |
| rpSP28 | RPRC000969 | DN29541_c0_g1_i1 |  |  |
| rpSP29 | RPRC006064 | DN29590_c0_g1_i1 |  |  |
| rpSP30-CLIP |  | DN297_c0_g2_i5-11 |  | HP21/18a/33/144 |
| rpSP31 | RPRC000816 | DN3021_c0_g1_i10 | 13318 |  |
| rpSP32 | RPRC001984 | DN3022_c0_g1_i9 | ModSP | HP14 |
| rpSP33 | RPRC004248 | DN3138_c0_g2_i1 |  |  |
| rpSP34 | RPRC007378 RPRC007381 | DN3142_c0_g1_i3 | ModSP | HP14 |
| rpSP35 | RPRC0073810 | DN3249_c0_g1_i1-7 | CG9372 | SPH1a/b |
| rpSP36 | RPRC004609 | DN33611_c0_g1_i1 |  |  |
| rpSP37 | RPRC015052 | DN33895_c0_g1_i1 |  |  |
| rpSP38 | RPRC013166 | DN36765_c0_g1_i1 |  |  |
| rpSP39 | RPRC004789 | DN3704_c0_g2_i5-10 |  | SP143 |
| rpSP40 | RPRC015054 | DN381_c0_g1_i36 |  |  |
| rpSP41 | RPRC000151 | DN3852_c1_g1_i3 | ModSP | HP14 |
| rpSP42 | RPRC004606 | DN3855_c0_g1_i14 |  |  |
| rpSP43 | RPRC000096 RPRC012201 | DN394_c0_g1_i14 | ModSP | HP14 |
| rpSP44 | RPRC015323 | DN3955_c0_g1_i2 |  |  |
| rpSP45 | RPRC010197 | DN402_c1_g1_i1-4 |  | HP21  18a  33  144 |
| rpSP46 | RPRC004609 | DN4492_c0_g1_i2 |  |  |
| rpSP47 | RPRC010306 | DN4570_c0_g1_i9 |  |  |
| rpSP48 | RPRC002919 | DN4830_c0_g1_i1-3 | CG14744 | SP131 |
| rpSP49 | RPRC010532 | DN4905_c0_g1_i5-11 |  | HP21  18a  33  144 |
| rpSP50 | RPRC012670 | DN4922_c0_g1_i1 |  |  |
| rpSP51 | RPRC002114 | DN4985_c0_g1_i3 |  |  |
| rpSP52 | RPRC002921 | DN5103_c0_g1_i6 | CG11066 |  |
| rpSP53 | RPRC005463 | DN5194_c0_g2_i2 | CG8172 | SP131 |
| rpSP54 |  | DN5264_c0_g1_i2 |  |  |
| rpSP55 | RPRC000973 | DN5514_c1_g1_i1 | CG13744 | SP140 |
| rpSP56 | RPRC001546 | DN5764_c0_g2_i1 |  |  |
| rpSP57-CLIP | RPRC012765 RPRC012767 | DN6179_c0_g2_i3 | Grass | SPH42/5 |
| rpSP58-CLIP | RPRC005400 | DN6835_c0_g1_i1-17 |  |  |
| rpSP59 | RPRC004954 | DN7180_c0_g1_i1 |  |  |
| rpSP60 | RPRC010335 | DN72_c0_g1_i7 |  |  |
| rpSP61 | RPRC014262 | DN7426_c0_g1_i1 |  |  |
| rpSP62 | RPRC001240 | DN778_c2_g1_i2 |  | SP89 |
| rpSP63-CLIP | RPRC013717 | DN89_c0_g2_i1 | CG31728 |  |
| rpSP64 |  | DN911_c0_g1_i1 |  |  |
| rpSP65 | RPRC001546 | DN977_c0_g1_i3 |  |  |
| rpSP66 | RPRC009335 | DN9965_c0_g1_i1 |  | SP3a |

Table S6. *Rhodnius prolixus* serine protease inhibitors (SPIs). List of SPIs identified in a *R. prolixus* fat body tissue transcriptome. The closest ortholog to these SPIs was identified in *Drosophila melanogaster* and *Manduca sexta* using phylogenetic analyses from Figure S2.

| Name | Vector base ID | Transcriptome ID | Closest ortholog in *Drosophila*  *melanogaster* | Closest  ortholog in *Manduca*  *sexta* |
| --- | --- | --- | --- | --- |
| rpSPI1 | RPRC000857 | DN11549_c0_g3_i5-1 | A1Z6I3 |  |
| rpSPI2 | RPRC000857 | DN11549_c0_g3_i7-2 | A1Z6I3 |  |
| rpSPI3 | RPRC012557 | DN1287_c1_g1_i13 | 27A | 3A |
| rpSPI4a |  | DN1287_c1_g1_i15-12 |  | 12 |
| rpSPI5 |  | DN1287_c1_g1_i4-2-14 |  | 2 |
| rpSPI4b |  | DN1287_c1_g1_i1-5 |  | 12 |
| rpSPI7a | RPRC012557 | DN1287_c1_g1_i5-2-11 | 27A | 3A |
| rpSPI7b | RPRC012557 | DN1287_c1_g1_i8-4 | 27A | 3A |
| rpSPI7c | RPRC012557 | DN1287_c1_g1_i9-1 | 27A | 3A |
| rpSPI4c |  | DN1287_c1_g1_i9-10 |  | 12 |
| rpSPI9a | RPRC002993 | DN1793_c0_g1_i1-69 | 85F | 7,5A,4b,4A, 9 |
| rpSPI9b | RPRC002993 | DN1793_c0_g1_i19-65 | 85F | 7,5A,4b,4A, 9 |
| rpSPI9c | RPRC002993 | DN1793_c0_g1_i2-22 | 85F | 7,5A,4b,4A, 9 |
| rpSPI9d | RPRC002993 p | DN1793_c0_g1_i32 | 85F | 7,5A,4b,4A, 9 |
| rpSPI9e | RPRC002993 | DN1793_c0_g1_i6 | 85F | 7,5A,4b,4A, 9 |
| rpSPI9f | RPRC002993 | DN1793_c0_g1_i68 | 85F | 7,5A,4b,4A, 9 |
| rpSPI10a | RPRC002795 | DN3262_c0_g1_i1 | 88E | 6 |
| rpSPI10b | RPRC002795 | DN3262_c0_g1_i3 | 88E | 6 |
| rpSPI10c | RPRC002795 | DN3262_c0_g1_i5 | 88E | 6 |
| rpSPI11a | RPRC004932 | DN3755_c0_g1_i2 | 28D | 13 |
| rpSPI11b | RPRC004932 | DN3755_c0_g1_i3a | 28D | 13 |
| rpSPI12 |  | DN3755_c0_g1_i3b |  |  |
| rpSPI13a | RPRC014952 | DN5163_c0_g1_i11 |  |  |
| rpSPI13b | RPRC014952 | DN5163_c0_g1_i2 |  |  |

Table S7. *Rhodnius prolixus* top 50 most highly expressed genes in the fat body. A list of highly expressed genes was generated from the 6 treatments used in the construction of a fat body transcriptome and the data set of naïve insects from Ribeiro et al 2014. All these genes have corresponding sequences in the *de novo* transcriptome. DE transcripts are highlighted in a grey background using the information from Table S1. Gr-: Gram-negative bacteria; Gr+: Gram-positive bacteria; hpi: hours post-injection.. Gr-: Gram-negative bacteria; Gr+: Gram-positive bacteria; hpi: hours post-injection.

| Gene ID | Gene name | Corresponding transcripts from the *de novo* transcriptome | Top 50 gene rank | | | | | | |
| --- | --- | --- | --- | --- | --- | --- | --- | --- | --- |
|  |  |  | **PBS 8hpi** | **Gr- 8hpi** | **Gr+ 8hpi** | **PBS 24hpi** | **Gr- 24hpi** | **Gr+ 24hpi** | **Naïve** |
| RPRC013002 | Prolixicin | DN3991_c0_g1 | 1 | 2 | 3 | 2 | 3 | 2 | - |
| RPRC010050 | Transferrin 1 | DN2187_c0_g2 | 2 | 1 | 1 | 1 | 2 | 1 | 13 |
| RPRC004803 | Defensin B | DN2263_c0_g1 | 3 | 5 | 2 | 4 | 1 | 4 | - |
| RPRC004408 | Histone-lysine_N-methyltransferase  SETMAR | DN148_c2_g1 | 4 | 3 | 5 | 5 | 5 | 3 | 2 |
| RPRC012379 | Unknown | DN585_c2_g1 | 5 | 4 | 4 | 3 | 4 | 6 | - |
| XLOC_007922 | Unknown | DN5109_c2_g1 | 6 | 6 | 6 | 15 | 12 | 7 | - |
| RPRC012186 | Defensin | DN2263_c0_g1 | 7 | 15 | 7 | 11 | 7 | 8 | - |
| RPRC006406 | Unknown | DN2586_c0_g1 | 8 | 13 | 12 | 16 | 13 | 5 | - |
| RPRC006873 | Unknown | DN6490_c0_g1, DN8267_c1_g1, DN27099_c0_g1, DN26995_c0_g1,  DN6490_c6_g1 | 9 | 26 | 9 | 18 | 8 | 11 | - |
| RPRC006743 | Unknown | DN5731_c0_g1 | 10 | 11 | 10 | 9 | 11 | 10 | 8 |
| RPRC006874 | Unknown | DN27204_c0_g1, DN6490_c0_g1 | 11 | 10 | 8 | 7 | 6 | 9 | 6 |
| RPRC012185 | Defensin A J | DN2263_c0_g1 | 12 | 19 | 11 | 14 | 10 | 12 | 48 |
| RPRC007160 | Unknown | DN76_c0_g2 | 13 | 18 | 17 | 19 | 17 | 13 | 3 |
| RPRC007034 | Tyrosine hydroxlase | DN3483_c0_g1 | 14 | - | 26 | - | 31 | 30 | - |
| RPRC015440 | Lysozyme | DN8775_c0_g1 | 15 | 12 | 13 | 8 | 9 | 18 | 10 |
| XLOC_016565 | Unknown | DN318_c0_g1 | 16 | 16 | 15 | 17 | 15 | 16 | - |
| RPRC012182 | Defensin | DN2263_c0_g1 | 17 | 8 | 14 | 10 | 14 | 17 | 7 |
| RPRC005884 | Aromatic-L-amino acid decarboxylase (AADC-2) | DN2173_c1_g1 | 18 | - | 32 | - | - | 51 | - |
| RPRC015041 | Elongation factor 1-alpha | DN2498_c0_g1 | 19 | 24 | 18 | 23 | 20 | 24 | - |
| XLOC_015883 | Pancreatic_lipase-related_protein_2 | DN105_c0_g3 | 20 | - | 33 | - | - | - | - |
| XLOC_002292 | Unknown | DN3211_c1_g1 | 21 | 7 | 16 | 6 | 16 | 19 | - |
| RPRC017359 | Polyadenylate-binding protein 1 | DN1497_c1_g1 | 22 | 27 | 20 | 25 | 24 | 29 | - |
| RPRC013481 | Unknown | DN1978_c0_g2 | 23 | 29 | 21 | 39 | 22 | - | - |
| RPRC003338 | Chitinase | DN16_c3_g1 | 24 | 9 | 19 | 12 | 18 | 14 | - |
| RPRC004310 | Heat shock protein cognate4n | DN3948_c0_g1 | 25 | 43 | 22 | 32 | 27 | 34 | - |
| RPRC007142 | Unknown | DN27393_c0_g1 | 26 | 21 | 23 | 31 | 23 | 15 | - |
| RPRC001419 | Unknown | DN441_c0_g2 | 27 | 20 | 29 | 21 | 25 | 26 | - |
| RPRC003090 | Rpsp9 | DN1612_c0_g2 | 28 | - | 55 | - | - | 35 | - |
| RPRC005400 | Phenoloxidase-activating factor 2 | DN6835_c0_g1/rpSP58 | 29 | 52 | 40 | 42 | 37 | 31 | - |
| RPRC002795 | Serpin | DN3262_c0_g1 | 30 | - | 54 | - | 54 | 55 | - |
| RPRC006872 | Glycine-rich cuticle protein | DN1519_c0_g1 | 31 | 17 | 24 | 26 | 34 | 21 | 33 |
| RPRC014133 | Unknown | DN443_c3_g1 | 32 | 32 | 35 | 55 | 26 | 20 | - |
| RPRC003547 | Unknown | DN1704_c0_g1 | 33 | 22 | 25 | 20 | 19 | 28 | - |
| XLOC_016372 | Gelsolin | DN68_c0_g1 | 34 | 14 | 30 | 13 | 21 | 22 | - |
| RPRC012274 | Uncharacterized protein | DN610_c1_g1 | 35 | 28 | 52 | 34 | 44 | 27 | - |
| RPRC004591 | Unknown | DN660_c0_g1 | 36 | - | 51 | - | 39 | 38 | - |
| RPRC000173 | Cuticle_protein_19.8 | DN1942_c0_g1 | 37 | 33 | 45 | 46 | 32 | 23 | - |
| RPRC010142 | Unknown | DN45_c1_g1 | 38 | - | - | - | - | - | - |
| RPRC004932 | Serpin/rpSPI11/12 | DN3755_c0_g1 | 39 | - | - | - | - | - | - |
| RPRC009807 | Unknown | DN2645_c0_g1 | 40 | - | - | - | - | - | - |
| XLOC_010962 | Unknown | DN49_c0_g1 | 41 | - | 53 | - | 40 | 50 | - |
| RPRC015103 | Uncharacterized protein | DN1001_c0_g1 | 42 | 23 | 36 | 24 | 29 | 25 | - |
| RPRC009366 | Unknown | DN7697_c0_g1 | 43 | 45 | 38 | 48 | 30 | - | - |
| RPRC009337 | Unknown | DN915_c1_g1 | 44 | 42 | 46 | 33 | 38 | 52 | - |
| RPRC010216 | Activating transcription factor 4 | DN2662_c0_g1 | 45 | 44 | 49 | 41 | 36 | 43 | - |
| RPRC014809 | Uncharacterized protein | DN3162_c0_g1 | 46 | - | 27 | - | 48 | - | - |
| RPRC009300 | Uncharacterized protein | DN1497_c1_g1 | 47 | 38 | 39 | 36 | 42 | 54 | - |
| RPRC001993 | Unknown | DN147_c2_g1 | 48 | 41 | 48 | 38 | 47 | 45 | - |
| RPRC009600 | Mitochondrial adp/atp carrier protein | DN10419_c0_g2 | 49 | 40 | 44 | 43 | 43 | 36 | - |
| RPRC007612 | Uncharacterized protein | DN640_c0_g2 | 50 | 35 | - | 29 | 46 | 32 | 15 |
| RPRC002089 | Unknown | DN2173_c0_g1 | 51 | - | 28 | - | 49 | - | - |
| RPRC003210 | GNBP | DN2658_c0_g1 | 52 | - | - | - | - | - | - |
| RPRC005937 | Unknown | DN364_c4_g1 | 53 | 36 | - | 37 | 53 | 33 | - |
| RPRC005966 | Uncharacterized protein | DN125_c1_g2 | 54 | - | 41 | 47 | 52 | - | - |
| RPRC003646 | Unknown | DN1257_c0_g1 | 55 | 39 | 47 | 35 | 33 | 40 | - |
| RPRC000603 | Nitrophorin-2 | DN168_c0_g1 | - | 25 | - | 22 | 28 | 46 | - |
| RPRC002452 | Major_facilitator_superfamily_domain-containing_protein_12 | DN1372_c3_g1 | - | 30 | - | 45 | - | 41 | - |
| RPRC000038 | Uncharacterized protein | DN1215_c1_g1 | - | 31 | - | 27 | - | 49 | - |
| RPRC011836 | Unknown | DN7282_c0_g2 | - | 34 | - | 28 | 41 | 42 | - |
| RPRC009367 | Unknown | DN1726_c0_g1 | - | 37 | - | 30 | - | - | 36 |
| RPRC005741 | Cysteine dioxygenase | DN6173_c0_g1 | - | 46 | - | 44 | - | 48 | 12 |
| RPRC004463 | Apolipoprotein d | DN4428_c0_g1 | - | 47 | - | 40 | - | - | - |
| RPRC009368 | Unknown | DN1954_c1_g1 | - | 48 | - | - | 50 | - | - |
| RPRC009875 | Actin-5C | DN12537_c0_g2 | - | 49 | 50 | 49 | 35 | - | - |
| RPRC000798 | Unknown | DN1747_c0_g3 | - | 50 | - | - | - | - | - |
| RPRC006828 | Unknown | DN683_c2_g1 | - | 51 | - | - | - | - | - |
| RPRC005736 | Bifunctional_purine_biosynthesis_protein_ATIC | DN6411_c0_g1 | - | 53 | - | - | - | - | - |
| RPRC003327 | 60S ribosomal protein LP0 | DN3256_c0_g1 | - | 54 | - | - | - | - | - |
| RPRC012162 | 60S ribosomal protein L4 | DN7825_c0_g2 | - | 55 | - | 53 | - | - | - |
| RPRC015199 | Unknown | DN10332_c0_g1 | - | - | 31 | - | - | - | - |
| RPRC011694 | Tubulin beta chain | DN1643_c1_g7 | - | - | 34 | - | - | - | - |
| RPRC010252 | Hsp70 protein | DN357_c3_g1 | - | - | 37 | - | 45 | 47 | - |
| RPRC003574 | Cytosol aminopeptidase | DN6614_c0_g1 | - | - | 42 | - | - | - | - |
| RPRC012247 | Unknown | DN416_c0_g1 | - | - | 43 | 52 | 55 | - | - |
| XLOC_001513 | Unknown | DN17641_c0_g1, DN260_c3_g1 | - | - | - | 50 | - | - | - |
| RPRC002646 | Unknown | DN1215_c1_g1 | - | - | - | 51 | - | - | - |
| RPRC013730 | Serine carboxypeptidase | DN30028_c0_g1 | - | - | - | 54 | - | - | - |
| RPRC011446 | Chitinase | DN11689_c0_g1, DN13091_c0_g1 | - | - | - | - | 51 | - | - |
| XLOC_004619 | SPARC | DN5058_c0_g1 | - | - | - | - | - | 37 | - |
| RPRC009414 | Cytochrome P450 (CYP44A) | DN3666_c0_g1 | - | - | - | - | - | 39 | - |
| RPRC014424 | Putative_inorganic_phosphate_cotransporter | DN7103_c1_g1 | - | - | - | - | - | 44 | - |
| RPRC006413 | Unknown | DN4699_c0_g1 | - | - | - | - | - | 53 | - |
| RPRC012101 | S-adenosylmethionine synthase | DN9104_c0_g1 | - | - | - | - | - | - | 1 |
| RPRC013511 | Vitellogenin lipoprotein | DN13466_c0_g1 | - | - | - | - | - | - | 4 |
| RPRC010951 | Unknown | DN4496_c0_g2 | - | - | - | - | - | - | 5 |
| RPRC000518 | Visgun mucin | DN1009_c3_g1 | - | - | - | - | - | - | 9 |
| RPRC004899 | Unknown | DN1994_c0_g1 | - | - | - | - | - | - | 11 |
| RPRC008183 | Unknown |  | - | - | - | - | - | - | 14 |
| RPRC006822 | Unknown | DN4644_c0_g1 | - | - | - | - | - | - | 16 |
| RPRC006658 | Unknown | DN6735_c0_g2 | - | - | - | - | - | - | 17 |
| RPRC013044 | Uncharacterized protein | DN1117_c3_g1 | - | - | - | - | - | - | 18 |
| RPRC014202 | Uncharacterized protein | DN1579_c0_g1 | - | - | - | - | - | - | 19 |
| RPRC007328 | Ribosomal protein p1 | DN18365_c0_g1 | - | - | - | - | - | - | 20 |
| RPRC014785 | Uncharacterized protein | DN707_c1_g1 | - | - | - | - | - | - | 21 |
| RPRC013497 | Uncharacterized protein | DN2937_c2_g1 | - | - | - | - | - | - | 22 |
| RPRC014587 | 60S ribosomal protein L36 | DN4972_c0_g1 | - | - | - | - | - | - | 23 |
| RPRC012177 | Defensin | DN503_c0_g1 | - | - | - | - | - | - | 24 |
| RPRC011918 | Uncharacterized protein | DN2558_c0_g1 | - | - | - | - | - | - | 25 |
| RPRC003739 | Unknown | DN8_c1_g2 | - | - | - | - | - | - | 26 |
| RPRC001811 | Nucleoside diphosphate kinase | DN7721_c0_g1 | - | - | - | - | - | - | 27 |
| RPRC010253 | Peritrophin | DN29_c0_g2 | - | - | - | - | - | - | 28 |
| RPRC009979 | Unknown | DN1745_c0_g1 | - | - | - | - | - | - | 29 |
| RPRC010146 | Unknown | DN11562_c0_g1 | - | - | - | - | - | - | 30 |
| RPRC002520 | 60S ribosomal protein L44 | DN9346_c0_g1 | - | - | - | - | - | - | 31 |
| RPRC014784 | Unknown | DN707_c1_g1 | - | - | - | - | - | - | 32 |
| RPRC007498 | Unknown | DN2817_c0_g1 | - | - | - | - | - | - | 34 |
| RPRC003799 | 40S ribosomal protein S30 | DN9465_c0_g1 | - | - | - | - | - | - | 35 |
| RPRC012448 | Uncharacterized protein | DN14215_c0_g1 | - | - | - | - | - | - | 37 |
| RPRC003032 | Unknown | DN4957_c0_g1 | - | - | - | - | - | - | 38 |
| RPRC007825 | Unknown | DN6619_c0_g1 | - | - | - | - | - | - | 39 |
| RPRC010192 | 60s ribosomal protein l26 | DN18411_c0_g1 | - | - | - | - | - | - | 40 |
| RPRC009914 | Unknown | DN4196_c0_g3 | - | - | - | - | - | - | 41 |
| RPRC005040 | Unknown | DN32646_c0_g1 | - | - | - | - | - | - | 42 |
| RPRC011123 | Splicing factor 3B subunit 5 | DN6564_c0_g1 | - | - | - | - | - | - | 43 |
| RPRC006865 | Small proline-rich protein | DN34861_c0_g1 | - | - | - | - | - | - | 44 |
| RPRC011797 | Unknown | DN6091_c0_g2 | - | - | - | - | - | - | 45 |
| RPRC000560 | Unknown | DN592_c3_g1 | - | - | - | - | - | - | 46 |
| RPRC003892 | Kazal-type serine protease inhibitor | DN15576_c0_g1 | - | - | - | - | - | - | 47 |
| RPRC008312 | Mitochondrial f1f0-atp synthase | DN4016_c0_g1 | - | - | - | - | - | - | 49 |

| **Table S8.** List of serine proteases (SPs) and serine protease inhibitors (SPIs) used for the construction of the phylogenetic trees depicted in Figures S1 and S2.. | | | | | | | |
| --- | --- | --- | --- | --- | --- | --- | --- |
| Serine proteases | | | | **Serine protease inhibitor** | | | |
| AgCLIPA10-XP_308802 | BmCLIP15-XP_004931455 | MsHP17a-Msex006845 | Aa-A0A0N8ERZ9 | | Bm-C0J8I1 | Dm-Q8MM39 |  |
| AgCLIPA3-EAA03300 | BmCLIP16-XP_004930740 | MsHP18a-Msex015895 | Aa-A0A0P6ITH6 | | Bm-C7ASM3 | Dm-Q8MPN5 |  |
| AgCLIPA4-XP_552464 | BmCLIP17-NP_001040462 | MsHP1a-Msex004508 | Aa-A0A0P6IYZ7 | | Bm-D2KQN9 | Dm-Q8MPN7 |  |
| AgCLIPA5-XP_320729 | BmCLIP1-NP_001036832 | MsHP21-Msex012584 | Aa-A0A0P6J4P6 | | Bm-H9IXK0 | Dm-Q8MQZ7 |  |
| AgCLIPA7-XP_320723 | BmCLIP2-NP_001036844 | MsHP28-Msex014495 | Aa-A0A1S4F2N4 | | Bm-H9J5D6 | Dm-Q8MSJ3 |  |
| AgCLIPA8-XP_311445 | BmCLIP3-NP_001040415 | MsHP5-Msex015451 | Aa-A0A1S4F3Y8 | | Bm-H9J5E5 | Dm-Q8MSJ4 |  |
| AgCLIPB10-XP_312744 | BmCLIP4-XP_012545493 | MsHP6-Msex000211 | Aa-A0A1S4F5E8 | | Bm-H9J5E6 | Dm-Q8SYY7 |  |
| AgCLIPB13-XP_314336 | BmCLIP5-XP_004933269 | MsHP8-Msex015451 | Aa-A0A1S4FHH9 | | Bm-H9JDX3 | Dm-Q8SZF4 |  |
| AgCLIPB14-XP_309876 | BmCLIP6-XP_012545524 | MsPAP1-Msex006497 | Aa-A0A1S4G0S7 | | Bm-H9JH33 | Dm-Q8T0M5 |  |
| AgCLIPB15-XP_318957 | BmCLIP7-XP_004926962 | MsPAP2-Msex011544 | Aa-A0A1S4G0X1 | | Bm-H9JH34 | Dm-Q8T3Z2 |  |
| AgCLIPB16-XP_320055 | BmCLIP8-XP_012548716 | MsPAP3-Msex011542 | Aa-J9HGR9 | | Bm-H9JKA2 | Dm-Q9U1I5 |  |
| AgCLIPB1-XP_307756 | BmCLIP9-XP_012551404 | MsSP131-Msex003029 | Aa-J9HT18 | | Bm-H9JL12 | Dm-Q9U1I6 |  |
| AgCLIPB2-XP_312956 | BmSP95-XP_012549295 | MsSP132-Msex003150 | Aa-J9HTU0 | | Bm-H9JL13 | Dm-Q9U1I7 |  |
| AgCLIPB3-XP_307750 | DmCG11066-AAF57320 | MsSP140-Msex004585 | Aa-O76292 | | Bm-H9JL15 | Dm-Q9U5W7 |  |
| AgCLIPB4-XP_307755 | DmCG1299-AAF47847 | MsSP142-Msex004592 | Aa-Q0IEW3 | | Bm-H9JWD5 | Dm-Q9U5W8 |  |
| AgCLIPB5-XP_313032 | DmCG13318-AAF54286 | MsSP143-Msex004604 | Aa-Q16H69 | | Bm-P22922 | Dm-Q9V3N1 |  |
| AgCLIPB8-XP_312743 | DmCG13744-AAF59005 | MsSP144-Msex011912 | Aa-Q16HB8 | | Bm-Q03383 | Dm-Q9V3Z2 |  |
| AgCLIPB9-XP_003436374 | DmCG15002-AAF47850 | MsSP30-Msex014494 | Aa-Q16P31 | | Bm-Q2F5W3 | Dm-Q9VA48 |  |
| AgCLIPC1-XP_552698 | DmCG17572-AAG22440 | MsSP33-Msex015895 | Aa-Q16S06 | | Bm-Q6Q2D5 | Dm-Q9VFC1 |  |
| AgCLIPC2-XP_313588 | DmCG31728-AAF53273 | MsSP52-Msex006835 | Aa-Q179D8 | | Bm-Q6Q2D6 | Dm-Q9VFC2 |  |
| AgCLIPC3-XP_313589 | DmCG6639-AAF53614 | MsSP60-Msex010785 | Aa-Q179E0 | | Bm-Q967V9 | Dm-Q9VH46 |  |
| AgCLIPD1-XP_312523 | DmCG7432-AAF55692 | MsSPH1a | Aa-Q17EW5 | | Bm-Q9BLL3 | Dm-Q9VII7 |  |
| AgCLIPD2-XP_317284 | DmCG8172-AAF59006 | MsSPH2-Msex013411 | Aa-Q17EW6 | | Bm-Q9NGS0 | Dm-Q9VL44 |  |
| AgCLIPD3-XP_321698 | DmCG8213-AAF59009 | MsSPH42-Msex000566 | Aa-Q17G63 | | Dm-A0A0B4K6Q4 | Dm-Q9VLQ7 |  |
| AgCLIPD4-XP_312102 | DmCG8586-AAF59059 | MsSPH53-Msex006849 | Aa-Q17HD5 | | Of-A0A1L7B974 | Dm-Q9VLU4 |  |
| AgCLIPD7-XP_319747 | DmCG9372-AAF49135 | OfSP10-Unigene941 | Aa-Q17HE1 | | Of-A0A1L7B975 | Dm-Q9VLV3 |  |
| AmSP10-XP_001120043 | DmCG9737-AAF57029 | OfSP12-Unigene3077 | Aa-Q17HE2 | | Of-V5L0E2 | Dm-Q9VLZ8 |  |
| AmSP14-XP_001121032 | DmEaster-AAF55170 | OfSP13-Unigene17162 | Aa-Q1HQG8 | | Dm-A0A126GUP6 | Dm-Q9VPH9 |  |
| AmSP1-XP_006570267 | DmGrass-AAF56675 | OfSP14-Unigene21915 | Aa-Q1HRV7 | | Dm-A1Z6I3 | Dm-Q9VVW1 |  |
| AmSP21-XP_006567247 | DmHayan-AAF48845 | OfSP17-Unigene21394 | Aa- **XP_001648011.** | | Dm-A1Z6R3 | Dm-Q9VWB4 |  |
| AmSP25-XP_006559396 | DmMODSP | OfSP1-CL399.Contig2 | Aa- **AAC31158.1** | | Dm-A1Z6R4 | Dm-X2JAJ8 |  |
| AmSP26-XP_006564973 | DmMP1-AAF52151 | OfSP2-Unigene18713 | Ag-A0A1S4GHQ9 | | Dm-A1Z6V5 | Dm- **NP_724512.1** |  |
| AmSP2-XM_006570203 | DmPersephone | OfSP37-CL5321.Contig1 | Ag-C6K043 | | Dm-A4V9T2 | Dm- **NP_652024.1** |  |
| AmSP33-XP_006559393 | DmSnake-AAF54897 | OfSP3-CL5234.Contig1 | Ag-C6K176 | | Dm-A4V9T3 | Dm- **CAB63098.1** |  |
| AmSP3-XP_006560620 | DmSp7-AAF54143 | OfSP4-Unigene13481 | Ag-F5HL11 | | Dm-A4V9T4 | Hs-Q9UIV8 |  |
| AmSP6-XP_006557876 | DmSPE-AAF56160 | OfSP5-CL1110.Contig5 | Ag-Q005M1 | | Dm-A4V9T5 | Ms-A0A3B6UET4 |  |
| AmSP7-XP_625051 | DmSpirit-AAF46392 | OfSP7-CL2452.Contig1 | Ag-Q005M2 | | Dm-A4VA01 | Ms-A0A3G1VCH1 |  |
| AmSPH41-XP_006563756 | DmStubble-AAF55277 | OfSP8-CL4641.Contig3 | Ag-Q005M5 | | Dm-A8JUP7 | Ms-A0A3G1VCH2 |  |
| AmSPH55-XP_001120817 | HsSP55-Q6UWB4 | OfSPH8-Unigene10046 | Ag-Q005M8 | | Dm-B3DMT4 | Ms-A0A3G1VCH4 |  |
| BmCLIP10-XP_012551892 | MsGP33-Msex016420 | OfSPH9-CL945.Contig1 | Ag-Q005N0 | | Dm-B9EQZ7 | Ms-E1B2D5 |  |
| BmCLIP11-NP_001037053 | MsHP12-Msex011546 | TmMSP-B1B5K3 | Ag-Q005N1 | | Dm-C0PVD9 | Ms-O02377 |  |
| BmCLIP12-XP_004927620 | MsHP14-Q69BL0 | TmSAE-B1B5K0 | Ag-Q5TX45 | | Dm-C5WLL8 | Ms-Q25491 |  |
| BmCLIP14-XP_004925813 | MsHP15-Msex014468 | TmSPE-B1B5K1 | Ag-Q7QBF4 | | Dm-F3YDI3 | Ms-Q6Q2D7 |  |
|  |  | TmSPH1-Q8I6J9 | Ag-Q7QJW6 | | Dm-H8F4V2 | Ms-Q7JQ67 |  |
|  |  |  | Ag-Q9NFT6 | | Dm-M9NEE3 | Ms-Q7JQ70 |  |
|  |  |  | Ag- **ABJ52806.1** | | Dm-Q0E8C8 | Ms-Q867T1 |  |
|  |  |  | Ag-**ABJ52801.1** | | Dm-Q0E8V6 | Ms- **ADM86478.1** |  |
|  |  |  | Ag- **XP_314159.2** | | Dm-Q3HKQ3 | Ms- **AAS68507.1** |  |
|  |  |  | Am- **XP_006562425.1** | | Dm-Q3HKQ7 | Ms- **AAC47341.1** |  |
|  |  |  | Am- **XP_003249882.1** | | Dm-Q4V3G2 | Ms- **AAV91026.1** |  |
|  |  |  | Ama- **ABS87358.1** | | Dm-Q4V3Q3 | Ms- **AAS68503.1** |  |
|  |  |  | Bm-B6DZ41 | | Dm-Q4V3R8 | Ir- **CAB55818.2** |  |
|  |  |  | Bm-C0J8G0 | | Dm-Q4V4T7 | Ir- **ABI94056.2** |  |
|  |  |  | Bm-C0J8G1 | | Dm-Q4V4W3 | Tm- BAL03254.1 |  |
|  |  |  | Bm-C0J8G2 | | Dm-Q4V6K6 | Tm- BAI59108.1 |  |
|  |  |  | Bm-C0J8G3 | | Dm-Q4V6M2 | Tm- BAI59107.1 |  |
|  |  |  | Bm-C0J8G8 | | Dm-Q6NP39 | Lb- ACQ83466.1 |  |
|  |  |  | Bm-C0J8G9 | | Dm-Q6V6U1 | **Rm-** **AHC98654.1** |  |
|  |  |  | Bm-C0J8H2 | | Dm-Q7JV69 | Aba- XP_001122067.2 |  |
|  |  |  | Bm-C0J8H4 | | Dm-Q7JWX3 | Aa- XP_001651231.2 |  |
|  |  |  | Bm-C0J8H6 | | Dm-Q7K1P6 | Am- XP_016772722.1 |  |
|  |  |  | Bm-C0J8H7 | | Dm-Q7K508 |  |  |
|  |  |  | Bm-C0J8H8 | | Dm-Q7K8Y3 |  |  |
|  |  |  | Bm-C0J8H9 | | Dm-Q7K8Y5 |  |  |
|  |  |  | Bm- **ACT36272.1** | | Dm-Q7KA66 |  |  |
|  |  |  | Bm- **ACG61181.1** | | Dm-Q7YTY6 |  |  |
